# Global transcriptome analysis of murine embryonic stem cell-derived cardiomyocytes

**DOI:** 10.1186/gb-2007-8-4-r56

**Published:** 2007-04-11

**Authors:** Michael Xavier Doss, Johannes Winkler, Shuhua Chen, Rita Hippler-Altenburg, Isaia Sotiriadou, Marcel Halbach, Kurt Pfannkuche, Huamin Liang, Herbert Schulz, Oliver Hummel, Norbert Hübner, Ruth Rottscheidt, Jürgen Hescheler, Agapios Sachinidis

**Affiliations:** 1 Center of Physiology and Pathophysiology, Institute of Neurophysiology, University of Cologne, Robert Koch Str., 50931 Cologne, Germany; 2 Max-Delbrueck-Center for Molecular Medicine - MDC, Robert-Rössle Str., 13092 Berlin, Germany; 3 Institute for Genetics, Department of Evolutionary Genetics, University of Cologne, Zülpicher Str., 50674 Cologne, Germany

## Abstract

**Background:**

Characterization of gene expression signatures for cardiomyocytes derived from embryonic stem cells will help to define their early biologic processes.

**Results:**

A transgenic α-myosin heavy chain (MHC) embryonic stem cell lineage was generated, exhibiting puromycin resistance and expressing enhanced green fluorescent protein (EGFP) under the control of the α-MHC promoter. A puromycin-resistant, EGFP-positive, α-MHC-positive cardiomyocyte population was isolated with over 92% purity. RNA was isolated after electrophysiological characterization of the cardiomyocytes. Comprehensive transcriptome analysis of α-MHC-positive cardiomyocytes in comparison with undifferentiated α-MHC embryonic stem cells and the control population from 15-day-old embryoid bodies led to identification of 884 upregulated probe sets and 951 downregulated probe sets in α-MHC-positive cardiomyocytes. A subset of upregulated genes encodes cytoskeletal and voltage-dependent channel proteins, and proteins that participate in aerobic energy metabolism. Interestingly, mitosis, apoptosis, and Wnt signaling-associated genes were downregulated in the cardiomyocytes. In contrast, annotations for genes upregulated in the α-MHC-positive cardiomyocytes are enriched for the following Gene Ontology (GO) categories: enzyme-linked receptor protein signaling pathway (GO:0007167), protein kinase activity (GO:0004672), negative regulation of Wnt receptor signaling pathway (GO:0030178), and regulation of cell size (O:0008361). They were also enriched for the Biocarta p38 mitogen-activated protein kinase signaling pathway and Kyoto Encyclopedia of Genes and Genomes (KEGG) calcium signaling pathway.

**Conclusion:**

The specific pattern of gene expression in the cardiomyocytes derived from embryonic stem cells reflects the biologic, physiologic, and functional processes that take place in mature cardiomyocytes. Identification of cardiomyocyte-specific gene expression patterns and signaling pathways will contribute toward elucidating their roles in intact cardiac function.

## Abstract 2

Microarray analysis reveals that the specific pattern of gene expression in cardiomyocytes derived from embryonic stem cells reflects the biological, physiological and functional processes occurring in mature cardiomyocytes.

## Background

Heart failure caused by loss of functional cardiomyocytes represents one of the most common cardiovascular diseases. Elucidation of the genetic networks and intracellular mechanisms that underlie cardiomyocyte development from ES cells is a prerequisite for future cell replacement therapies in heart failure [1,2]. Recently, genetic strategies for differentiation of stem cells and nonmuscle cells through expression of developmental control genes that specify cardiac cell identity have been favoured in cell replacement therapies to regenerate heart muscle tissue [3]. However, a prerequisite for these strategies is identification and an understanding of cardiac cell-specific biologic, physiologic, and molecular processes. To this end, signaling pathways and gene signatures characteristic of cardiomyocytes must be deciphered in order to characterize the cardiomyocytes derived from embryonic stem (ES) cells.

Mouse ES cells can proliferate indefinitely without senescence *in vitro *in their undifferentiated state in the presence of leukemia inhibitory factor or on a layer of mitotically inactivated mouse embryonic fibroblasts (MEFs) [4]. ES cells can be genetically manipulated with reporter and selection markers to identify and select cardiomyocytes from differentiating ES cells [5-8]. Most often, protocols to enrich cardiomyocytes from transgenic cardiac cell lines were optimized for ES cell lines such as the D3 cell line cultivated on MEFs. It is well known that several, as yet uncharacterized factors from MEFs have an influence on the differentiation processes of ES cells, necessitating the use of MEF-free ES cells in differentiation studies [9]. Recently, we clearly demonstrated that the first contact with MEFs contaminates ES cells even if they are subsequently cultivated in the absence of MEFs, and the gene expression profile of MEFs interferes with those of ES cells and embryoid bodies (EBs). Even 9-day-old EBs are still contaminated by MEFs, and MEF-specific gene expression is still detectable [9]. Therefore, consistent gene expression and developmental studies on ES cells require MEF-free ES cells.

Although MEF-dependent, ES cell derived cardiomyocytes have been well characterized electrophysiologically [5-8], the cardiac-specific gene signatures and signaling cascades had not until now been characterized in detail. Even though several attempts have been made, a comprehensive transcriptome analysis of MEF-free murine ES cell derived pure cardiomyocytes is not yet available.

We recently reported an optimized CGR8 ES cell model that permits consistent gene expression and facilitates studies of the early embryonic development [9]. In order to identify all signal transduction pathways and biologic processes in cardiomyocytes, we generated a transgenic cardiomyocyte-specific cell line from CGR8 mouse ES cells and isolated pure cardiomyocytes. Thereafter, large-scale expression studies were performed using Affymetrix expression microarrays covering all known transcripts. Here we report, for the first time, a transcriptome analysis of pure cardiomyocyte preparations from MEF-free ES cells.

Similar to findings mature cardiomyocytes, we demonstrate that cardiomyocytes derived from ES cells strongly express classic genes that are required to accomplish their physiologic function. Interestingly, the genes required for cell proliferation and apoptotic processes are significantly downregulated in ES-derived cardiomyocytes. We may conclude that the identification of 'gene signatures' and signal transduction pathways that are specifically expressed in the α-myosin heavy chain (MHC)-positive cell population will significantly contribute to an understanding of cardiomyocyte-specific physiologic processes.

## Results and discussion

### Isolation of highly purified α-MHC^+ ^cardiomyocytes from the transgenic α-MHC embryonic stem cell line

**Figure 1:**
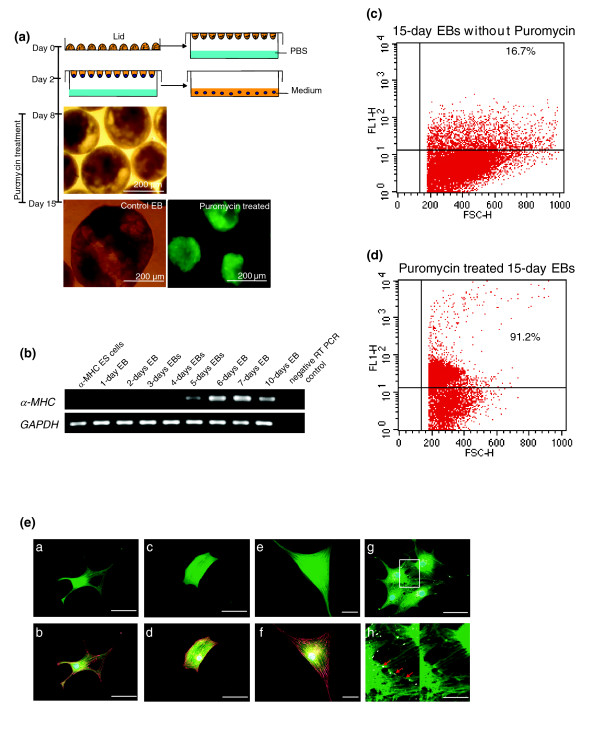
Enrichment of α-MHC^+ ^cells isolated from the α-MHC^+ ^ES cell lineage after puromycin treatment. **(a) **Progressive purification of α-myosin heavy chain (MHC)^+ ^cardiac cell aggregates after treatment of the 8-day-old embryoid bodies (EBs) with 4 μg/ml puromycin for 7 days. Puromycin containing medium was refreshed every second day. **(b) **Reverse transcription (RT)-polymerase chain reaction (PCR) analysis of α-MHC expression during EB differentiation (for RT-PCR conditions and primers, see Additional data file 3). **(c,d) **Cells from 15-day-old EBs and 15-day-old puromycin purified α-MHC^+ ^aggregates were dissociated by trypsinization and the purity of the α-MHC^+ ^cells in the 15-day-old EBs (panel c) and in the 15-day-old α-MHC^+ ^aggregates (panel d) was examined by fluorescence-activate cell sorting analysis. **(e) **Characterization of ES cell derived cardiomyocytes by immunocytochemistry. α-MHC^+ ^cardiomyocytes were dissociated with collagenase B and plated on fibronectin coated coverslips. **(e) **Enhanced green fluorescent protein (EGFP) expression of single α-MHC^+ ^cells with different morphologies (subpanels a, c, and e). Detection of α-cardiac actinin (subpanels b, d, and f) and connexion-43 (subpanels g and h) was performed using anti-cardiac actinin (1:400) and anti-connexin-43 (1:400). Secondary detection was performed with anti-mouse-IgG_1_-AlexaFluor555 and anti-rabbit-Ig-AlexaFluor647. Hoechst dye was used to stain nuclei. Bars in panel e (subpanels a to f) are 50 μm; bar in panel e (subpanel g) is 20 μm; and bar in panel (subpanel h) is 7.5 μm.

We first generated cardiomyocytes with high purity from a transgenic α-MHC ES cell line. When EBs were formed using the conventional hanging drop method (Figure 1a) during the course of differentiation, the EGFP fluorescence increased significantly after 7 days and the EGFP-expressing cells were first detectable microscopically within the EBs. After 24 hours, the 8-day-old EBs were treated with 4 μg/ml puromycin for a further 7 days. During puromycin treatment the non-puromycin-resistant cells died, and beating clusters of puromycin-resistant 15-day-old EGFP-expressing α-MHC^+ ^cells were progressively enriched (Figure 1a and Additional data files 1 and 2).

Reverse transcription (RT)-polymerase chain reaction (PCR) analysis indicated maximal expression of the α-MHC^+ ^gene in the 7-day-old EBs (Figure 1b; for RT-PCR conditions and primers, see Additional data file 3). The purity of the cardiomyocytes in the 15-day-old untreated EBs (hereafter referred to as 'control EBs') and in the 15-day-old α-MHC^+ ^cardiomyocyte EBs was determined by fluorescence-activated cell sorting analysis after dissociation of the cells with trypsin and calculated to be 16.7% (Figure 1c) and 91.2% (Figure 1d), respectively.

The ES cell derived cardiomyocytes exhibited a multi-angular (Figure 1e subpanels a and b), more rectangular (Figure 1e, subpanels c and d), and a triangular morphology (Figure 1e, subpanels e and f). Detection of cardiac α-actinin by immunocytochemistry (Figure 1e, subpanels b, d and f) clearly indicated the Z-disc specific protein and the characteristic striations of sarcomeric structures of the cardiac cells. The gap junction protein connexin-43 is highly expressed in heart and was detected by immunocytochemistry (Figure 1e, subpanels g and h). Connexin-43 is distributed in the cytosol and in the outer membranes in the cell border regions (Figure 1e, subpanels g and h).

### Electrophysiological characterization of the cardiomyocytes

**Figure 2:**
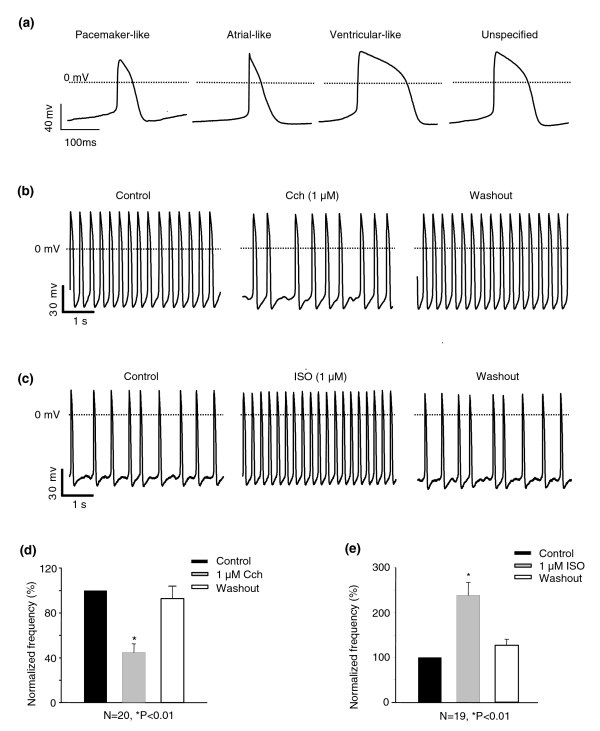
Electrophysiological characterisation of α-MHC^+ ^cells. **(a) **Characteristic cardiac action potential (APs) of puromycin purified α-myosin heavy chain (MHC)^+ ^cells. Most APs had a typical cardiac AP morphology but could not be further specified. Only few APs exhibited typical features of pacemaker-like, atrial-like, or ventricular-like APs. The minimal diastolic potential was -60.2 ± 1.1 mV. The maximal upstroke velocity was 22.9 ± 2.2 V/s. APD_90_, APD_50 _and APD_20 _(AP duration from maximum to 90%, 50% and 20% repolarization) were 96.4 ± 4.2 ms, 71.1 ± 3.9 ms, and 41.3 ± 2.6 ms, respectively. Representative recordings showing the effect of **(b) **carbachol (1 μmol/l) and **(c) **isoproterenol (1 μmol/l) on the spontaneous AP frequency. Statistical analysis of the effects of **(d) **carbachol (1 μmol/l) and **(e) **isoproterenol (1 μmol/l) on the spontaneous AP frequency. Carbachol caused a decrease whereas isoproterenol increased the spontaneous AP frequency.

Functional characterization of the α-MHC^+ ^cardiac cells was performed by measuring their typical spontaneous action potentials (APs). Spontaneous APs were measured in single α-MHC^+ ^cardiomyocytes (*n *= 32) as well as in multicellular α-MHC^+ ^aggregates (*n *= 24). All APs exhibited parameters characteristic of cardiac APs. The minimal diastolic potential was -60.2 ± 1.1 mV; membrane potentials normally showed a diastolic depolarization, leading to a spontaneous AP frequency of 125.9 ± 8.0 min. The maximal upstroke velocity was 22.9 ± 2.2 V/s, pointing to a contribution of voltage-activated sodium currents, which was confirmed by voltage clamp measurements (data not shown). APD_90_, APD_50 _and APD_20 _(AP duration from maximum to 90%, 50% and 20% repolarization) were 96.4 ± 4.2 ms, 71.1 ± 3.9 ms and 41.3 ± 2.6 ms, respectively. APs exhibited a variety of morphologies, including pacemaker-like, atrial-like, and ventricular-like APs (Figure 2). In most cases, however, morphologic properties did not match any type of specific differentiation. These unspecified APs mostly possessed a plateau phase, but had a much shorter APD_90 _than ventricular APs, which are characterized by a long APD_90 _of about 200 ms [7].

To characterize the hormonal regulation of α-MHC cardiomyocytes, carbachol (an agonist of m-cholinoceptors) and isoproterenol (an agonist of β_1 _adrenoceptors) were applied (Figure 2b,c). Carbachol at 1 μmol/l decreased the AP frequency significantly, to 44.8 ± 7.5% of control values (*n *= 20; the frequency under control conditions was determined for each recording and set to 100; Figure 2d). Isoproterenol at 1 μmol/l evoked a significant increase in frequency to 238.58 ± 23.7% of control values (*n *= 19; Figure 2e).

Intracellular recordings of spontaneous APs revealed typical cardiac AP parameters and morphologies, confirming the cardiac differentiation and functionality of puromycin-selected α-MHC^+ ^cells. Muscarinic and adrenergic regulation of the AP frequency, which is estabished for ES cell derived cardiomyocytes [10] as well as for native murine cardiomyocytes at early developmental stages [11], further supports a physiologic cardiac differentiation of α-MHC cells. As described previously [12,13], APs at the intermediate developmental stage exhibited diastolic depolarizations and diverse shapes. APs with a distinct plateau phase were frequent but considered to be unspecific rather than ventricular-like in the majority of cases, because the APD_90 _was much shorter than reported for early-stage murine ventricular cardiomyocytes as well as for murine ES cell derived ventricular-like cardiomyocytes [7]. Because most APs had unspecific morphologic properties, a general classification into pacemaker-like, atrial-like, and ventricular-like APs could not be done, which accords well with previous findings from intermediate-stage ES cell derived cardiomyocytes [12,13]. Only few APs exhibited typical morphologic features of the respective differentiation types.

It was recently reported that, in ES cell derived cardiomyocytes expressing green fluorescent protein under control of the α-MHC promotor, green fluorescence is restricted to pacemaker-like and atrial-like cells [7]. Because we found puromycin-purified α-MHC^+ ^cardiomyocytes with a ventricular-like AP morphology in few cases, our data suggest that α-MHC expression is not completely absent in ES cell derived ventricular-like cardiomyocytes. This apparent discrepancy might arise from the complex stage-dependent expression pattern described for α-MHC in murine EBs [14] and murine embryonic ventricles [15], because a different developmental stage of ES cell derived cardiomyocytes was investigated in the present study (15-day-old cardiomyocytes) as compared with that in the study conducted by Kolossov and coworkers [7] (9-day-old to 11-day-old cardiomyocytes).

### Validation of the microarray data by quantitative real-time PCR and semiquantitative RT-PCR analyses

**Figure 3:**
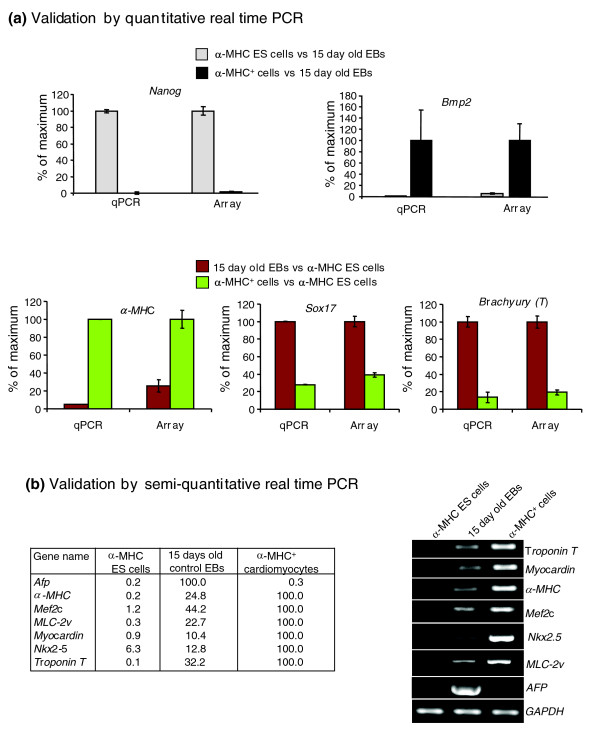
Validation of Affymetrix data by quantitative real-time PCR and semi-quantitative PCR analyses. **(a) **Validation of Affymetrix data by quantitative real-time polymerase chain reaction (PCR) analyses. The fold change was calculated by using the following formula: fold-change = 2−(ΔCtgene1−ΔCtgene2)
 MathType@MTEF@5@5@+=feaafiart1ev1aaatCvAUfeBSjuyZL2yd9gzLbvyNv2Caerbhv2BYDwAHbqedmvETj2BSbqee0evGueE0jxyaibaiKI8=vI8tuQ8FMI8Gi=hEeeu0xXdbba9frFj0=OqFfea0dXdd9vqai=hGuQ8kuc9pgc9s8qqaq=dirpe0xb9q8qiLsFr0=vr0=vr0dc8meaabaqaciGacaGaaeqabaqadeqadaaakeaacaaIYaWaaWbaaSqabeaacqGHsislcaGGOaGaeuiLdqKaae4qamaaBaaameaacaqG0baabeaaliaabEgacaqGLbGaaeOBaiaabwgacaaIXaGaeyOeI0IaeuiLdqKaae4qamaaBaaameaacaqG0baabeaaliaabEgacaqGLbGaaeOBaiaabwgacaaIYaGaaiykaaaaaaa@46CC@. ΔC_t _of the gene in the sample in which it is expressed lowest is taken as ΔC_t _gene2 to calculate the fold change using the above formula. The resulting fold change is expressed as percentage of the maximum fold change (= 100%) for that particular gene in every assay. Values are expressed as mean ± standard deviation (*n *= 3; technical replicates). **(b) **Additional validation of Affymetrix data by semi-quantitative reverse transcription (RT)-PCR analyses. Randomly chosen candidate genes to validate Affymetrix data by semi-quantitiative RT-PCR analyses and their relative expression values expressed as percentage of maximum expression for every gene, as obtained from Affymetrix profiling, are given in the table.

RNA from α-MHC ES cells, 15-day old α-MHC^+ ^cells, and control EBs was used as a template for hybridizations to Affymetrix MG 430 v2.0 oligonucleotide microarrays (RNA was obtained from three independent experiments) (Affymetrix UK Ltd., **High Wycombe, UK**). Raw expression data were RMA normalized [16]. We verified the Affymetrix data by examining the expression levels of five randomly chosen representative genes (*Nanog*, *T Brachyury*, *Bmp2*, *Sox17*, and α-*MHC*) applying the quantitative real-time PCR (qPCR) method (Figure 3a). Additionally, expression levels of randomly chosen genes, such as *Troponin T*, *Myocardin*, α-*MHC*, *Mef2C*, *Nkx2.5*, *MLC-2v*, and *AFP *were verified by semiquantitative RT-PCR analysis (Figure 3b). As indicated, the expression levels of the late cardiomyocyte markers α-MHC and MLC2v and the early cardiac marker Nkx2.5 were higher in the 15-day-old α-MHC^+ ^cardiomyocytes as compared with cells in the 15-day-control EBs. Not surprisingly, expression of α-fetoprotein (a marker of cell types of endodermal origin, such as liver cells) was absent in the cardiomyocyte clusters but not in the 15-day-old control EBs. As indicated in Figure 3 panels a and b, results from the Affymetrix analyses clearly correspond to the results obtained from the qPCR and semiquantitative RT-PCR analyses, respectively. Note that RNA used in Figure 3a for qPCR validation was isolated from set of experiments other than that used in Figure 3b.

### Selected Gene Ontology Biologic Process annotations of genes differentially expressed in α-MHC^+ ^cardiomyocytes

Pair-wise comparisons between experimental conditions were performed on RMA-normalized data using Student's *t*-test (unpaired, assuming unequal variance). In order to identify transcripts with an α-MHC^+ ^cell specific expression pattern, a three-condition comparative analysis of the α-MHC^+ ^cells versus control EBs and versus α-MHC ES cells was made (intersection of genes differentially expressed between undifferentiated α-MHC ES cells and α-MHC^+ ^EBs, as well as differentially expressed between 15-day-old control EBs and α-MHC^+ ^EBs at *t*-test *P *< 0.01, fold change >2).

**Figure 4:**
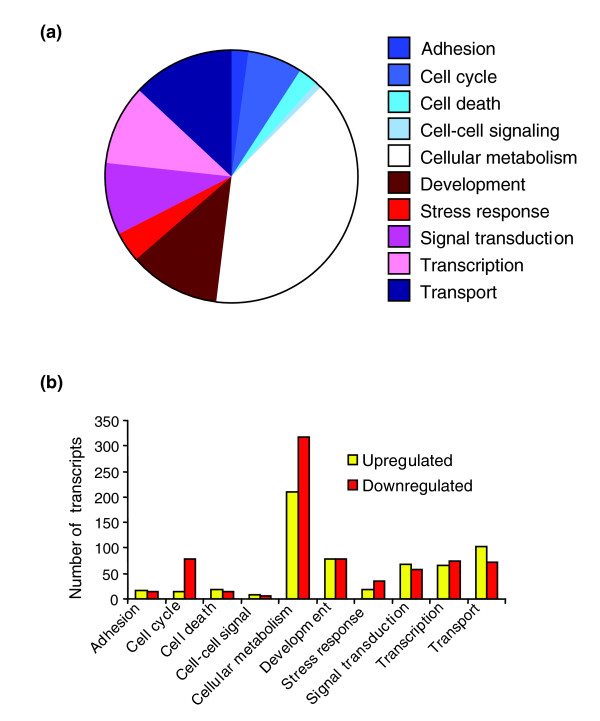
Selected GO annotations of genes differentially expressed in α-MHC^+ ^cells. Shown are selected Gene Ontology (GO) annotations (biologic process [BP]) of genes that are differentially expressed in α-MHC^+ ^cells as compared with the 15-day-old embryoid bodies (EBs) and compared with the α-MHC embryonic stem (ES) cells. A total of 1,845 probe set IDs, which were differentially expressed in α-MHC^+ ^cells, were converted to Genbank accessions and redundancies were removed. SOURCE was used to obtain GO BP annotations. Genesis was used to visualize and identify GO BP categories of interest and extract corresponding lists of transcripts. **(a) **The pie chart shows the distribution of these annotations. **(b) **The bar chart shows the number of genes in the categories adhesion, cell cycle, cell death, cell-cell signalling, cellular metabolism, development, stress response, signal transduction, transcription, and transport separately for upregulated and downregulated transcripts.

Analysis of the differentially expressed genes in the α-MHC^+ ^cells in comparison with the control EBs and undifferentiated α-MHC ES cells resulted in identification of 1,845 differentially expressed probe sets for the α-MHC^+ ^cardiomyocytes. Affymetrix probe set IDs were then converted to Genbank accessions and redundancies were removed (1,573 unique transcripts). SOURCE [17] was used to obtain Gene Ontology (GO) annotations for the category 'biologic process'. The Genesis GO browser (version 1.7.0) [18,19] was used to identify transcripts of interest belonging to the biologic process categories adhesion, cell cycle, cell death, cell-cell signaling, cellular metabolism, development, stress response, signal transduction, transcription, and transport. For these categories, 1,346 annotations were established for 823 transcripts. The pie chart (Figure 4a) shows the distribution of these annotations. The bar chart (Figure 4b) shows the number of genes in the categories separately for upregulated and downregulated transcripts. Most strikingly, transcripts in the category cell cycle are almost exclusively downregulated.

### Gene Ontology enrichment analysis of the genes upregulated in α-MHC^+ ^cardiomyocytes

To identify GO categories and Kyoto Encyclopedia of Genes and Genomes (KEGG) pathways specifically enriched among transcripts upregulated in α-MHC^+ ^cells, we identified 884 probe sets that are upregulated at least twofold (*t*-test *P *< 0.01) in the α-MHC^+ ^cardiomyocytes as compared with the control EBs (consisting of various somatic cells, including an α-MHC^+ ^subpopulation) and compared with the undifferentiated α-MHC ES cells.

**Figure 5:**
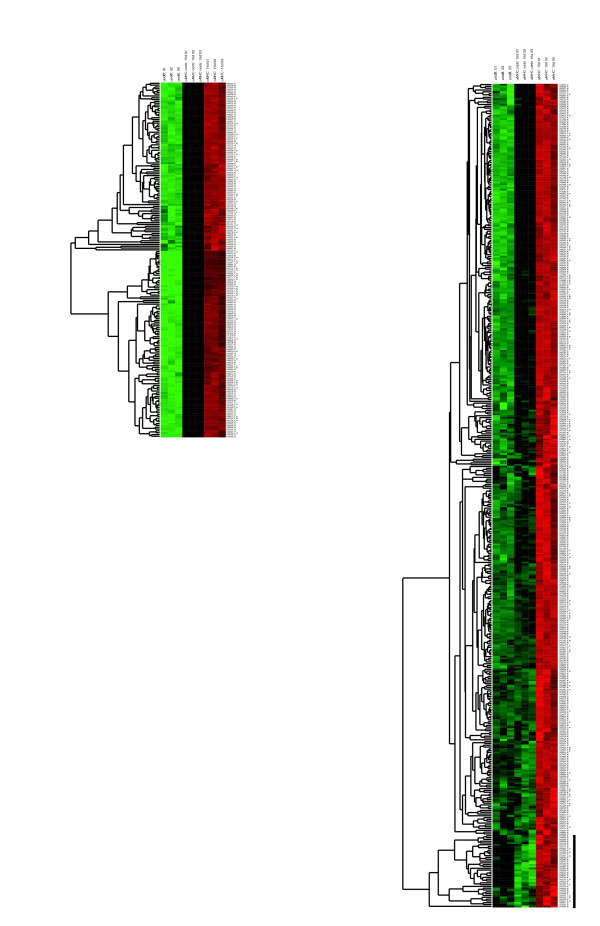
Hierarchical clustering of probe sets identified as upregulated in α-MHC^+ ^cells. Shown is a visualization of the hierarchical clustering of probe sets identified as upregulated in the α-myosin heavy chain (MHC)^+ ^cells with an expression level at least twofold higher than in 15-day-old EBs and in α-MHC embryonic stem cells. Each probe set is represented by a single row of colored boxes; each array is represented by a single column. Rectangles corresponding to intermediately expressed probe sets are colored black, upregulated probe sets are indicated with red of increasing intensity, and weakly expressed probe sets with green of increasing intensity. The dendrogram on the left of the figure represents the similarity matrix of probe sets.

Probe sets belonging to non-annotated RIKEN clones and expressed sequence tag sequences were removed. The remaining 652 probe sets were clustered hierarchically (Figure 5). Expression patterns are characteristic of cardiomyocytes (last three lanes), showing high expression levels as compared with undifferentiated α-MHC ES cells (first three lanes) and compared with the cells from the control EBs. The gene names correlated to relative expression level are given in Additional data file 4.

Two subclusters were identified. Subcluster A (196 genes) includes genes with low expression level in undifferentiated cells, moderate expression in the control EBs, and high expression levels in the α-MHC^+ ^cardiomyocytes. Cluster B (455 genes) includes genes with low expression in both control EBs and undifferentiated ES cells but with higher expression in α-MHC^+ ^cells. Interestingly, the expression level of a subset of genes (highlighted in Figure 5b) was higher in undifferentiated cells as compared with control EBs but lower than that in α-MHC^+ ^cells.

The DAVID (Database for Annotation, Visualization, and Integrated Discovery) tools were used to identify functional annotation terms in the categories of GO (level 5), KEGG pathway, and Biocarta pathway that are enriched in the lists of upregulated and downregulated transcripts. Table 1 indicates the KEGG and GO terms that are enriched in the 884 probe sets over-expressed in the α-MHC^+ ^cardiomyocytes. We identified two KEGG pathways (oxidative phosphorylation and calcium signaling) and a Biocarta pathway (p38 MAPK [mitogen-activated protein kinase] signaling pathway). Among the GO categories of 'biologic process' (GOTERM_BP), 'molecular function' (GOTERM_MF), and 'cellular component' (COTERM_CC), several categories associated with aerobic energy production (for instance, mitochondrion, hydrogen ion transporter activity, cytochrome *c *oxidase activity, and oxidative phosphorylation) were found to be enriched in probe sets that were over-expressed in the α-MHC^+ ^cardiomyocytes. In addition, several classic 'cardiomyocyte' cytoskeleton GO categories (for example, myofibril, cytoskeleton, myosin, and actin cytoskeleton) and the 'voltage-gated ion channel activity' GO category were found to be enriched in the cardiac population. All of these genes are necessary for intact cardiomyocyte function. Additional data file 5 (part a) lists the genes that belong to the GO categories striated muscle thin filament and myosin. As indicated, all cardiac-specific cytoskeletal genes are highly upregulated in ES cell derived cardiomyocytes. As shown in Additional data file 5 (part b), several voltage-gated channels such as the sodium channels, the calcium voltage-dependent channels, and the potassium channels are among the probe sets upregulated in the ES derived cardiomyocytes participating in the AP shape of the cardiac cells. These findings clearly indicate that the α-MHC^+ ^cardiomyocytes express classical cardiomyocyte genes, emphasizing the relevance and consistency of the gene signatures characteristic of the ES derived cardiomyocytes.

The 'oxidative phosphorylation' KEGG pathway is associated with aerobic energy production (also see below) whereas calcium is the second messenger regulating several physiologic processes such as contractility in cardiomyocytes [20]. Additional data file 6 (part a) shows the gene expression level changes of transcripts belonging to the oxidative phosphorylation KEGG pathway and the corresponding signal transduction scheme. Additional data file 6 (part b) shows the upregulated probe sets of the GO categories that participate in aerobic energy production and their increase in expression level. In general, the dependence of cardiac homeostasis on mitochondria is primarily attributed to the ATP derived from oxidative phosphorylation for maintaining myocardial contractility [21]. Genes in these categories are 'classical' for cardiomyocytes and essential for aerobic oxygen dependent energy production for intact heart function. Mammalian heart muscle cells fail to produce enough energy under anaerobic conditions to maintain essential cellular processes. Because the mammalian heart is an obligate aerobic organ that consumes oxygen intensively [22], a constant supply of oxygen is indispensable for sustaining cardiac function and viability. This notion is well elucidated by our analysis, indicating that genes involved in aerobic energy production are upregulated in the α-MHC^+ ^cardiomyocytes.

We also found the fatty acid metabolism GO category to be enriched in the genes over-expressed in the α-MHC^+ ^cardiomyocytes (Additional data file 6 [part c]). These findings are consistent with the fact that β-oxidation of fatty acids in mitochondria accounts for the vast majority of ATP generation and therefore is the preferred substrate in the adult myocardium, which supplies about 70% of total ATP (for review, see Huss and Kelly [23]). Defects in mitochondrial fatty acid transport and fatty acid oxidation result in sudden cardiac death, bioenergetic dysfunction, cardiac arrhythmias, and cardiomyopathy [21].

Genes that are specifically expressed in α-MHC^+ ^cells participate in multiple signal transduction pathways. Additional data file 7 (parts a and b) show that the genes belonging to the 'enzyme linked receptor protein signaling pathway' and to 'protein kinase activity' categories are over-expressed in ES cell derived cardiomyocytes. Among these genes, the phosphatidylinositol 3-kinase (Additional data file 7 [part a]) and the Wnt inhibitory factor 1 (Additional data file 7 [part b]) and several other kinases participate in key biologic signal transduction pathways.

Interestingly, five genes belonging to the category 'negative regulation of Wnt receptor signaling pathway' and four genes belonging to 'p38 mitogen-activated protein kinase signaling' were found to be upregulated in the α-MHC^+ ^cells (see Additional data file 7 [parts c and d]). In recent years, regulation of Wnt signal transduction has been discussed as an important event that initiates cardiac development (for review, see Eisenberg and Eisenberg [24]). It has been demonstrated that the Wnt inhibitors Dkk1 and crescent induce cardiogenesis, suggesting that Wnts actively inhibit cardiogenesis.

A number of genes belonging to the KEGG signaling pathway 'calcium signaling' were upregulated in α-MHC^+ ^cells. Apart from classic genes for cardiomyocytes (those encoding ryanodine receptor 2, phospholamban, calcium channel voltage dependent L-type, adrenergic receptor β_1_, troponin C, and Atp2a2), additional genes were identified as being over-expressed in the α-MHC^+ ^cells (*Cacna1c *and *Cacna1h*, *Nfatc2*, and *Gnal*; Additional data file 7 [part e]). Seven genes belonging to the GO category 'regulation of cell size" are upregulated in the ES cell derived cardiomyocytes (Additional data file 7 [part f]). Notably, among the 'regulation of cell size' genes, the myocardin gene was highly upregulated (10-fold) in the cardiac population. Myocardin is the founding member of a class of muscle transcription factors that is involved in the process of cardiomyogenesis [25]. Moreover, the estrogen receptor α, a member of the superfamily of steroid hormone receptors, is strongly upregulated (ninefold) in the cardiomyocytes. Estrogen receptors are activated by estrogen binding and by growth factors in the absence of estrogen (for review, see Mendelsohn and Karas [26]).

### Gene Ontology enrichment analysis of the downregulated genes in α-MHC^+ ^cardiomyocytes

In order to identify transcripts that are specifically downregulated in α-MHC^+ ^cells, a three-condition comparative analysis of the α-MHC^+ ^cells versus control EBs and versus α-MHC ES cells was made (intersection of genes differentially expressed between undifferentiated α-MHC ES cells and α-MHC^+ ^EBs as well as differentially expressed between 15-day-old control EBs and α-MHC^+ ^EBs at *t*-test *P *< 0.01, fold change <2 for both comparisons).

**Figure 6:**
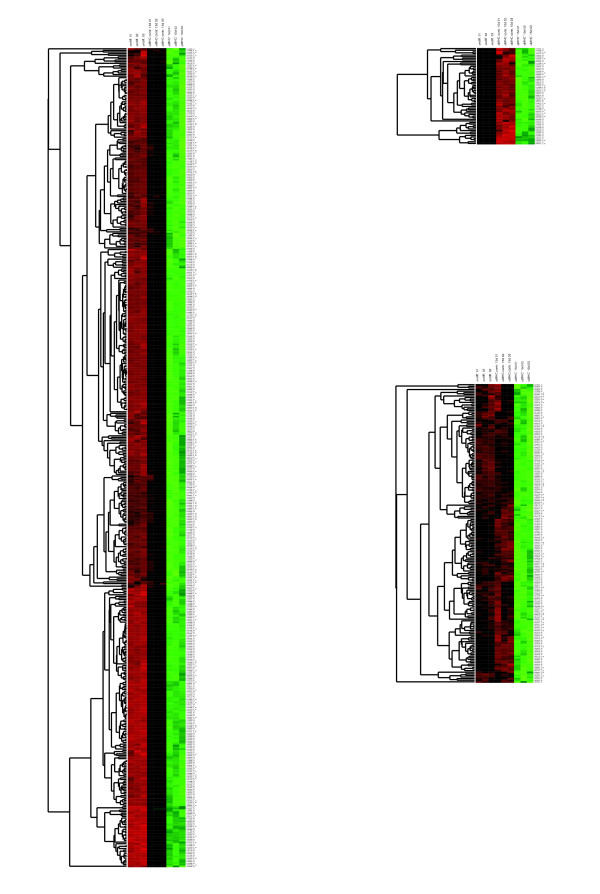
Hierarchical clustering of probe sets identified as downregulated in α-MHC^+ ^cells. Shown is a visualization of the hierarchical clustering of probe sets identified as downregulated in the α-MHC^+ ^cells with an expression level at least twofold lower than in 15-day-old EBs and in α-MHC embryonic stem cells. Each probe set is represented by a single row of colored boxes; each array is represented by a single column. Rectangles corresponding to intermediately expressed probe sets are colored black, upregulated probe sets are indicated with red of increasing intensity, and weakly expressed probe sets with green of increasing intensity. The dendrogram on the left of the figure represents the similarity matrix of probe sets.

Downregulated genes are grouped into three main subclusters. In all subclusters the gene expression level is lower in α-MHC^+ ^cells (last three lanes) compared with those in the other two cell populations. Subcluster A contains probe sets with a higher expression level in undifferentiated ES cells compared with the control EBs, whereas cluster C shows the genes with a similar expression level in the two cell populations (Figure 6; gene names are given in Additional data file 8). Only a small cluster contains genes with a higher expression level in control EBs compared with the undifferentiated ES cells (Figure 6b).

Table 2 shows the GO categories, and the KEGG and Biocarta pathways that are enriched among the probe sets downregulated in the α-MHC^+ ^cardiac cell population compared with the control EBs and compared with the undifferentiated α-MHC ES cells. Genes belonging to eight KEGG and 10 Biocarta pathways were identified as being downregulated in the ES cell derived cardiomyocytes (Table 2). Of particular interest are the genes that are associated with cell proliferation belonging to the KEGG pathway 'cell cycle' (32 genes) and to the 10 Biocarta pathways, as well as genes belonging to the GO category 'positive regulation of programmed cell death', because both the proliferation and apoptosis process are of great physiologic relevance. In accordance with the findings from the KEGG and Biocarta pathways, the GO category 'regulation of cell cycle' (also includes genes of the mitotic cell cycle and the M-phase GO categories) is enriched among the transcripts downregulated in the ES derived cardiomyocytes. This category contains genes that are involved in regulating cell proliferation.

Additional data file 9 (part a) indicates the downregulated genes that belong to the KEGG pathway 'cell cycle' and to the GO category 'cell cycle'. As indicated several cyclins (cyclin A_1_, cyclin A_2_, cyclin B_1_, cyclin B_2_, cyclin D_1_, cyclin D_2_, and cyclin E_1_) are downregulated in the ES cell derived cardiomyocytes. Fully differentiated cardiac myocytes are postmitotic cells, and regulation of cell size is their main mechanism of adaptation to variations in workload in the heart [27]. Increase in cell size of the cardiomyocytes due to increased protein synthesis without cell division is a characteristic phenomenon for cardiac cells and is referred as myocyte hypertrophy. These findings are consistent with the finding that 'cell growth' genes are upregulated in the cardiac cell population (Additional data file 7 [part f]). In agreement with these cardiac cell specific mechanisms, we found that - compared with the mixed cell population - genes belonging to the 'positive regulation of programmed cell death' (apoptosis) and to the 'apoptosis' GO categories are downregulated in the cardiac cell population (Additional data file 9 [part c]). Markedly, the expression level of the apoptosis-inducing gene (mainly in cancer cells) breast cancer 1 [28] was 13-fold lower in α-MHC^+ ^cardiomyocytes than in the mixed cell population of control EBs. In addition, it is well known that cardiac myocytes have developed remarkable mechanisms of cytoprotection and cell survival, also because of the absence of cell division [27].

To determine whether the identified gene signatures are specific to α-MHC^+ ^cardiomyocytes and are not due to puromycin treatment, we generated a transgenic β-actin ES cell line expressing both puromycin resistance and EGFP cassettes under the control of the β-actin promoter. After application of the differentiation protocol used for isolation of the α-MHC^+ ^cardiomyocytes (Figure 1), RNA was isolated from 15-day-old EBs either in the presence or in the absence of puromycin. Because β-actin is constitutively highly expressed in all cell types, the β-actin EBs were resistant to puromycin. Accordingly, no morphologic alterations were observed compared with 15-day-old control untreated EBs.

Analysis of the differentially expressed genes in the 15-day-old β-actin EBs in the absence of puromycin in comparison with the 15-day-old β-actin EBs treated with 4 μg/ml puromycin resulted in 554 differentially expressed probe sets (at *t*-test *P *< 0.01, fold change >2). Among the 554 probe sets, 31 are found also to be differentially expressed in the α-MHC^+ ^cardiomyocytes. Interestingly, all of the 31 probe sets are downregulated in the 15-day-old β-actin EBs (Additional data file 10). Although 20 of these probe sets are downregulated in the 15-day-old β-actin EBs because of puromycin treatment, they are upregulated in the α-MHC^+ ^cardiomyocytes enriched by puromycin treatment. Therefore, these 20 genes can be considered to be cardiomyocyte specific.

A group of 11 probe sets corresponding to 10 genes is downregulated in puromycin treated β-actin EBs and in cardiomyocytes. However, in comparison with 1,845 probe sets specifically regulated in cardiomyocytes, 10 genes account for a negligible fraction. Hence, our expression profiling remains of importance within the context of defining the cardiomyocyte specific transcriptome.

## Conclusion

After the completion of several mammalian genome sequencing projects, the next challenge will be to identify the complete cell specific transcriptome. Identification of the complete transcriptome of the ES cell derived somatic cells will contribute to our understanding of the developmental processes that lead from undifferentiated ES cells to tissue-specific cells. Here, we identified all α-MHC^+ ^cardiomyocyte specific gene expression signatures, including signal transduction pathways that are characteristic for cardiomyocytes. From our results, we conclude that - similar to mature cardiomyocytes - cardiomyocytes derived from ES cells strongly express genes required for regulation of respiratory energy metabolism, cytoskeleton, and ion channels; these genes are required for cardiomyocytes to fulfil their physiologic function. Interestingly, as in postmitotic adult cardiomyocytes, genes required for cell proliferation are significantly downregulated, whereas genes involved in the process of cell growth were significantly upregulated in the ES cell derived cardiomyocytes. Moreover, genes that are involved in the process of apoptosis were significantly downregulated in the ES cell derived cardiomyocytes. Finally, identification of the gene signatures and signal transduction pathways that are specifically expressed in the α-MHC^+ ^cell population will contribute to establishment of a gene expression atlas for cardiomyocytes and will be useful for further studies to elucidate their role during developmental processes.

## Materials and methods

### Embryonic stem cell culture and differentiation of embryoid bodies

CGR8 ES cells (ECACC 95011018) were cultured without feeder cells in Glasgow minimum essential medium (GMEM) supplemented with 10% fetal bovine serum, 2 mmol/l L-glutamine, 100 units/ml leukemia inhibitory factor, and 50 μmol/l β-mercaptoethanol (ME) in 0.2% gelatine coated flasks, as described previously [9]. The cells were passaged every other day and care was taken to maintain the confluency below 70%. The cells were passaged at least four times before use in the experiments to attain a stable gene expression profile. To induce differentiation, the hanging drop protocol was used, as described previously [9]. Briefly, an ES cell suspension of 2.5 × 10^4 ^cells/ml was prepared in Iscove's modified Dulbecco's Medium (IMDM) supplemented with 20% fetal calf serum, 1% non-essential amino acids (vol/vol), 2 mmol/l L-glutamine, and 100 μmol/l β-ME. Of this ES cell suspension, 20 μl was spotted on the inside of the upper lid of a 10 cm bacteriologic dish and then covered over its bottom dish containing 5 ml phosphate-buffered saline. On day 2, the formed multicellular aggregates (EBs) were transferred to suspension in a new dish with 20 ml IMDM supplemented with 20% fetal calf serum, 1% non-essential amino acids (vol/vol), 2 mmol/l L-glutamine, and 100 μmol/l β-ME. On day 8, contracting clusters were observed. At this stage, cultures were either treated with puromycin (4 μg/ml) or left without treatment for 7 days with a medium change on every other day. On day 15, the beating clusters were trypsinized and used for fluorescence-activated cell sorting analysis or for RNA extraction.

### Vector constructs and cell line generation

pIRES2 EGFP was purchased from Clontech (Heidelberg, Germany). The human cytomegalovirus (CMV) immediate early promoter and enhancer were removed by a double digestion with *Nhe*I and *Ase*I and then subsequently blunt end ligated to obtain pIRES2 EGFP Δ CMV. Puromycin *N*-acetyl transferase cDNA was flanked on both ends by *Bam*HI restriction sites, which was PCR-amplified from pIRESPuro3 by *Pfu *DNA polymerase (Promega, Germany) and inserted at the *Sma*I site of the pIRES2 EGFP Δ CMV construct to obtain pPuro IRES2 EGFP. The 5.5 kilobase α-MHC promoter construct was a kind gift from Dr J Robbins (Cincinnati, OH, USA) [29]. The promoter was klenowed on one end and the other end digested with *Sac*I. The resulting product was ligated to the vector pPuro IRES2 EGFP processed in the same way to generate p α-MHC^p ^Puro IRES2 EGFP. This α-MHC^p ^reporter construct drives the expression of both puromycin resistance and EGFP under the control of α-MHC promoter by the use of IRES sequence. To generate the α-MHC ES line, the construct was electroporated in CGR8 cells with 500 μF and 240 V in a Bio-Rad Gene Pulser™ (Bio-Rad, Hercules, CA, USA). The transfected clones were selected by 400 μg/ml neomycin, and after selection the clones were maintained with 200 μg/ml neomycin. During EB generation, the neomycin selection was discontinued.

To generate pβactin^p ^Puro IRES2 EGFP, the 1.7 kilobase β-actin promoter was excised from pCAGGS plasmid (kindly provided by Dr Miyazaki, Tohoku University, Tohoku, Japan) with *Eco*RI and *Sal*I and blunt-end ligated to *Eco*RI-digested, klenowed, and 5'-dephosphorylated pPuro IRES2 EGFP. Generation of the transgenic β-actin^+ ^ES cell line expressing both puromycin resistance and EGFP was performed as described for the α-MHC ES lineage using the linearized pβactin^p ^Puro IRES2 EGFP.

### Immunohistochemistry

EBs were plated on 6-well format tissue culture dishes. From day 8 the cells were treated with 4 μg/ml puromycin for the subsequent 7 days. Afterward, cells were dissociated with 1 mg/ml collagenase B (Roche, Mannheim, Germany) for 30 min. Dissociated cells were plated on fibronectin (Sigma, Taufkirchen, Germany) coated coverslips for 3 days. Fixation was done with 4% paraformaldehyde for 30 min. Samples were permeabilized with 0.5% Triton-X100 (Sigma) and 0.5 M ammonium chloride. Blocking was performed in 5% bovine serum albumin (Sigma). Incubations with the primary antibodies anti-α-actinin (Sigma; clone EA-53; 1:400) and anti-connexin 43 (Sigma; clone CXN-6; 1:400) were performed overnight at 4°C in 1% bovine serum albumin. After extensive washing, secondary detection was performed for 2 hours at room temperature. Anti-mouse IgG_1_-AlexaFluor555 and anti-rabbit-Ig-AlexaFluor647 were purchased from Molecular Probes (Invitrogen GmbH, Karlsruhe, Germany). Hoechst dye was used to stain nuclei. Samples were mounted using ProLong Gold Mounting medium (Molecular Probes) and observed with a Zeiss Axiovert 200 fluorescence microscope.

### Patch clamp and sharp electrode electrophysiological studies

AP recordings were performed in single α-MHC cardiomyocytes and in multicellular α-MHC^+ ^cell aggregates at days 11 to 16. Single cardiomyocytes were obtained by dissociating puromycin-purified α-MHC^+ ^cell aggregates using a protocol described previously [30] and incubated in Dulbecco's modified Eagle medium plus 20% fetal calf serum at 37°C and 5% carbon dioxide for 24 to 36 hours before measurements were taken. APs of spontaneously beating single α-MHC^+ ^cardiomyocytes were measured by means of the whole-cell current-clamp technique using an EPC-9 amplifier and the PULSE software package (Heka Elektronik, Lambrecht, Germany). Patch clamp electrodes had a resistance of 3-4 MΩ when filled with intracellular solution containing (in mmol/l) 50 KCl, 80 KAspartate, 1 MgCl_2_, 3 MgATP, 10 EGTA and 10 HEPES (pH 7.4).

Spontaneous electrical activity of cardiomyocytes within plated α-MHC^+ ^cell aggregates was assessed by conventional microelectrode recordings. The resistance of the sharp electrodes, which were filled with 3 M KCl, was 20 to 50 MΩ. Signals were acquired by a SEC-10LX amplifier (Npi Electronic, Tamm, Germany) connected to the PULSE software via the interface of the EPC-9.

All experiments were performed at 37°C in standard extracellular solution containing (in mmol/l) 140 NaCl, 5.4 KCl, 1.8 CaCl_2_, 1 MgCl_2_, 10 HEPES and 10 glucose, or 136 NaCl, 5.4 KCl, 0.33 NaH_2_PO_4_, 1 MgCl_2_, 10 glucose, 5 HEPES and 1.8 CaCl_2 _(pH 7.4, adjusted with NaOH). All electrophysiologic data were analyzed off-line with custom made analysis software (kindly provided by Philipp Sasse).

### Semiquantitative RT-PCR analysis

Total RNA was extracted using RNeasy Mini Kit with on-column DNase I digestion, in accordance with the manufacturer's instructions (Qiagen, Hilden, Germany). Total RNA 5 μg was reverse transcribed using SuperScript II Reverse transcriptase with random primers, in accordance with the manufacturer's recommended protocol (Invitrogen GmbH, Karlsruhe, Germany). PCR amplification was done with REDTaq ReadyMix (Sigma) with 0.4 μmol/l each primer. GAPDH was used as an internal control. The following conditions were used: an initial denaturation at 95°C for 2 min, followed by 22 to 35 cycles of 30 s denaturation at 95°C, 30 s annealing at 60°C, and 60 s of elongation at 72°C. A final extension at 72°C for 5 min was included. Electrophorectic separation of PCR products was carried out on 2% agarose gels with 0.001% ethidium bromide. The primer sequences are listed in the Additional data file 3.

### Quantitative real-time PCR

Validation of the Affymetrix data was performed by qPCR analysis with the ABI Prism 7900HT Sequence Detection System (Applied Biosystems, Foster City, CA, USA). A total of 100 ng RNA of α-MHC ES cells, 15-day-old control Ebs, and α-MHC^+ ^cells were reverse transcribed with ThermoScript™ Reverse Transcriptase (Invitrogen). Then, real-time PCR was perfomed in triplicates for every sample using TaqMan Gene Expression Assays (Applied Biosystems). The Gene Expression Assays used for validation were *Brachyury (T) *(Mm00436877_m1), *Sox17 *(Mm00488363_m1), α-*MHC *(Mm00440354_m1), *Bmp2 *(Mm01340178_m1), *GAPDH *(Mm99999915_g1), and *Nanog *(Mm02019550_s1). Averaged C_t _values of each qPCR reaction from the target gene were normalized with the average C_t _values of the housekeeping gene *GAPDH*, which ran in the same reaction plate to obtain the ΔC_t _value. The fold change was calculated as follows: fold change = 2−(ΔCtgene1−ΔCtgene2)
 MathType@MTEF@5@5@+=feaafiart1ev1aaatCvAUfeBSjuyZL2yd9gzLbvyNv2Caerbhv2BYDwAHbqedmvETj2BSbqee0evGueE0jxyaibaiKI8=vI8tuQ8FMI8Gi=hEeeu0xXdbba9frFj0=OqFfea0dXdd9vqai=hGuQ8kuc9pgc9s8qqaq=dirpe0xb9q8qiLsFr0=vr0=vr0dc8meaabaqaciGacaGaaeqabaqadeqadaaakeaacaaIYaWaaWbaaSqabeaacqGHsislcaGGOaGaeuiLdqKaae4qamaaBaaameaacaqG0baabeaaliaabEgacaqGLbGaaeOBaiaabwgacaaIXaGaeyOeI0IaeuiLdqKaae4qamaaBaaameaacaqG0baabeaaliaabEgacaqGLbGaaeOBaiaabwgacaaIYaGaaiykaaaaaaa@46CC@. Because the genes included are not expressed in at least one of the three conditions (α-MHC ES cells, 15-day-old control Ebs, or α-MHC^+^), the ΔC_t _of the gene in the sample with the lowest expression is used as ΔC_t _gene2 to calculate the fold change using the above formula. The resulting fold change is expressed as percentage of the maximum fold change.

### Flow cytometry

A single cell suspension was prepared by trypsinization. Cell clumps were removed by passing through cell strainer cap of a round bottom tube from Falcon^® ^**(**Becton Dickinson GmbH, Heidelberg,, Germany). Propidium iodide (PI) staining (Sigma) was included to exclude dead cells. Acquisition of 10,000 live (PI-negative) cells was made with FACscan (BD Biosciences) and the data were analyzed using the CellQuest software (BD Biosciences). The respective wild-type EBs on the same day as the sample EBs were used as controls.

### Affymetrix analysis

Total RNA was extracted from undifferentiated ES cells and EBs using the RNeasy total RNA isolation kit (Qiagen GmbH, Hilden, Germany). The preparation quality was assessed by agarose-formaldehyde gel electrophoresis. Three independent total RNA preparations, each 1 μg from the α-MHC^+ ^cells, from the mixed cell population and from the undifferentiated ES cells, were labelled with the One-Cycle Target Labeling and Control Reagent package (Affymetrix), as described in the manufacturer's instructions. Briefly, double-stranded cDNA was synthesized using the one-cycle cDNA synthesis module. Biotinylated cRNA was synthesized using the IVT labeling kit and cleaned up using the sample cleanup module.

After fragmenting of the cRNA for target preparation using the standard Affymetrix protocol, 15 μg fragmented cRNA was hybridized for 16 hours at 45°C to Mouse Genome 430 2.0 arrays, which carry probes representing 45,101 probe sets. Following hybridization, arrays were washed and stained with streptavidin-phycoerythrin in the Affymetrix Fluidics Station 450 and further scanned using the Affymetrix GeneChip Scanner 3000 7G. The image data were analyzed with GCOS 1.4 using Affymetrix default analysis settings. After RMA normalization [31], three pair-wise comparisons were performed using Student's *t*-test (unpaired, assuming unequal variances). A Student's *t*-test *P *value < 10^-2 ^and a fold change >2 between two conditions was used to define differential expression. An intersection of upregulation in α-MHC^+ ^cardiomyocytes (twofold; *t*-test *P *value < 0.01) compared with control cells in the 15-day-old EBs (d15) and compared with undifferentiated α-MHC ES cells (d0) was used to identify transcripts upregulated in α-MHC^+ ^cardiomyocytes. For downregulation, an intersection of downregulation in α-MHC^+ ^cardiomyocytes (twofold, *t*-test *P *value < 0.01) compared with control cells in the 15 day control EBs (d15) and compared with undifferentiated α-MHC ES cells (d0) was used.

Hierarchical clustering was done using Cluster version 3.0 [32,33] applying mean centering and normalization of genes before average linkage clustering with uncentered correlation.

### Functional annotation

Differentially expressed genes were analyzed according to predefined pathways and functional categories annotated by KEGG [34], Biocarta [35], and GO [36] using the DAVID bioinformatic resource [37]. For an over-represented GO or KEGG pathway, a cutoff *P *value of 0.01 has been selected. In general, it should be noted that one gene can participate in more than one KEGG or Biocarta pathway and GO category.

## Additional data files

The following additional data are available with the online version of this paper. Additional data file 1 provides a video clip showing the 15-day-old untreated EBs. Additional data file 2 provides a video clip showing the 15-day-old puromycin treated EBs. Additional data file 3 provides the RT-PCR conditions and primers used in the RT-PCR experiments. Additional data file 4 provides the lists of probe sets used for the subclusters A and B, as identified in the hierarchical clustering of probe sets upregulated in α-MHC^+ ^cells (Figure 5). Additional data file 5 summarizes genes of various GO categories that are upregulated in the α-MHC^+ ^cardiomyocytes compared with control cells in the 15-day-old EBs and compared with undifferentiated α-MHC ES cells. Additional data file 6 summarizes genes belonging to the KEGG pathway 'oxidative phosphorylation' and various GO categories that are upregulated in α-MHC^+ ^cardiomyocytes as compared with control cells in the 15-day-old EBs and compaed with undifferentiated α-MHC ES cells; also provided is a schematic of the KEGG oxidative phosphorylation pathway. Additional data file 7 summarizes genes belonging to various GO categories and the Biocarta pathway 'p38 mitogen-activated protein kinase signaling' that are upregulated in the α-MHC^+ ^cardiomyocytes compared with control cells in the 15-day-old EBs and compared with undifferentiated α-MHC ES cells. Additional data file 8 provides lists of probe sets for the subclusters A, B, and C, as identified in the hierarchical clustering of probe sets downregulated in α-MHC^+ ^cells (Figure 6). Additional data file 9 summarizes genes belonging to the KEGG pathway 'cell cycle', various GO categories, and Biocarta Pathways 'G1/S checkpoint' and 'G2/M checkpoint' that are downregulated in α-MHC^+ ^cardiomyocytes compared with control cells in the 15-day-old control EBs and compared with undifferentiated α-MHC ES cells. Additional data file 10 lists probe sets both differentially regulated by puromycin treatment in 15-day-old β-actin EBs and transcripts upregulated in α-MHC^+ ^cells compared with the control cells in the 15-day-old EBs and compared with undifferentiated α-MHC ES cells. Additional data file 11 lists probe sets differentially regulated in the puromycin-treated 15-day-old β-actin^+ ^EBs. Additional data file 12 provides raw data for the α-MHC experiments (part 1). Additional data file 13 provides raw data for the α-MHC experiments (part 2). Additional data file 14 provides raw data for the β-actin control experiments.

## Supplementary Material

Additional data file 1 Provided is a video clip showing the 15-day-old untreated EBs. Click here for file

Additional data file 2 Provided is a video clip showing the 15-day-old puromycin treated EBs. Click here for file

Additional data file 3 Summarized are the RT-PCR conditions and primers used for the RT-PCR experiments. Click here for file

Additional data file 4 Provided are lists of probe sets for the subclusters A and B, as identified in the hierarchical clustering of probe sets upregulated in α-MHC^+ ^cells (Figure 5). Click here for file

Additional data file 5 Part a provides the genes belonging to the GOTERM_CC categories 'myofibril, striated muscle thin filament, actin cytoskeleton', 'cytoskeleton' and 'myosin', and GOTERM_BP category 'cytoskeleton organization and biogenesis' that are upregulated in the α-MHC^+ ^cardiomyocytes (intersection of upregulation in α-MHC^+ ^cardiomyocytes [twofold, *t*-test *P *value < 0.01] compared with control cells in the 15-day-old EBs and compared with undifferentiated α-MHC ES cells). Part b provides the genes belonging to the GOTERM_MF categories 'voltage-gated ion channel activity' that are upregulated in the α-MHC^+ ^cardiomyocytes (intersection of upregulation in α-MHC^+ ^cardiomyocytes [twofold, *t*-test *P *value < 0.01] compared with control cells in the 15-day-old EBs and compared with undifferentiated α-MHC ES cells). Click here for file

Additional data file 6 Part a provides genes belonging to the KEGG pathway 'oxidative phosphorylation' that are upregulated in α-MHC^+ ^cardiomyocytes (intersection of upregulation in α-MHC^+ ^cardiomyocytes [twofold, *t*-test *P *value < 0.01] compared with control cells in the 15-day-old EBs and compared with undifferentiated α-MHC ES cells) and a schematic of the KEGG oxidative phosphorylation pathway. Part b provides the genes belonging to the GOTERM_CC_5 categories 'mitochondrion', 'mitochondrial membrane' and 'mitochondrial electron transport chain', and GOTERM_MF_5 categories 'hydrogen ion transporter activity', 'NADH dehydrogenase (quinone) activity' and 'sodium ion transporter activity' that are upregulated in α-MHC^+ ^cardiomyocytes (intersection of upregulation in α-MHC^+ ^cardiomyocytes [twofold, *t*-test *P *value < 0.01] compared with control cells in the 15-day-old EBs and compared with undifferentiated α-MHC ES cells). Part c provides genes belonging to the GOTERM_CC category "fatty acid metabolism" that are upregulated in the α-MHC^+ ^cardiomyocytes (intersection of upregulation in α-MHC^+ ^cardiomyocytes [twofold, *t*-test *P *value < 0.01] compared with control cells in the 15-day-old EBs and compared with undifferentiated α-MHC ES cells). Click here for file

Additional data file 7 Part a provides genes belonging to the GOTERM_BP_5 category 'enzyme linked receptor protein signaling pathway' that are upregulated in the α-MHC^+ ^cardiomyocytes (intersection of upregulation in α-MHC^+ ^cardiomyocytes [twofold, *t*-test *P *value < 0.01] compared with control cells in the 15-day-old EBs and compared with undifferentiated α-MHC ES cells). Part b provides genes that belong to the GOTERM_MF_5 category 'protein kinase activity' that are upregulated in α-MHC^+ ^cardiomyocytes (intersection of upregulation in α-MHC^+ ^cardiomyocytes [twofold, *t*-test *P *value < 0.01] compared with control cells in the 15-day-old EBs and compared with undifferentiated α-MHC ES cells). Part c provides genes belonging to the GOTERM_BP_5 categories 'negative regulation of Wnt receptor signaling pathway' and 'negative regulation of signal transduction' that are upregulated in α-MHC^+ ^cardiomyocytes (intersection of upregulation in α-MHC^+ ^cardiomyocytes [twofold, *t*-test *P *value < 0.01] compared with control cells in the 15-day-old EBs and compared with undifferentiated α-MHC ES cells). Part d provides genes belonging to the Biocarta pathway 'p38 mitogen-activated protein kinase signaling" that are upregulated in α-MHC^+ ^cardiomyocytes (intersection of upregulation in α-MHC^+ ^cardiomyocytes [twofold, *t*-test *P *value < 0.01] compared with control cells in the 15-day-old EBs and compared with undifferentiated α-MHC ES cells). Part e provides genes belonging to the KEGG pathway 'Calcium Signalling' that are upregulated in α-MHC^+ ^cardiomyocytes (intersection of upregulation in α-MHC^+ ^cardiomyocytes [twofold, *t*-test *P *value < 0.01] compared with control cells in the 15-day-old EBs and compared with undifferentiated α-MHC ES cells). Part f provides genes belonging to the GOTERM_BP_5 'Regulation of cell size' that are upregulated in α-MHC^+ ^cardiomyocytes (intersection of upregulation in α-MHC^+ ^cardiomyocytes [twofold, *t*-test *P *value < 0.01] compared with control cells in the 15-day-old EBs and compared with undifferentiated α-MHC ES cells). Click here for file

Additional data file 8 Provided are lists of probe sets for the subclusters A, B, and C, as identified in the hierarchical clustering of probe sets downregulated in α-MHC^+ ^cells (Figure 6). Probe sets are listed with the corresponding gene symbol and gene title. Click here for file

Additional data file 9 Part a provides genes belonging to the KEGG pathway 'cell cycle' as well as to the GO BP terms 'M-phase', 'mitotic cell cycle', and 'regulation of cell cycle' that are downregulated in α-MHC^+ ^cardiomyocytes (intersection of downregulation in α-MHC^+ ^cardiomyocytes [twofold, *t*-test *P *value < 0.01] compared with control cells in the 15-day-old control EBs and compared with undifferentiated α-MHC ES cells). It also provides a schematic of the KEGG cell cycle pathway, indicating the downregulated genes. Part b provides genes belonging to the BIOCARTA pathways 'G1/S checkpoint' and 'G2/M checkpoint' that are downregulated in α-MHC^+ ^cardiomyocytes (intersection of downregulation in α-MHC^+ ^cardiomyocytes [twofold, *t*-test *P *value < 0.01) compared with control cells in the 15-day-old control EBs and compared with undifferentiated α-MHC ES cells). Part c provides genes belonging to the GOTERM_BP_5 categories 'positive regulation of programmed cell death' that are downregulated in α-MHC^+ ^cardiomyocytes (intersection of downregulation in α-MHC^+ ^cardiomyocytes [twofold, *t*-test *P *value < 0.01] compared with control cells in the 15-day-old control EBs and compared with undifferentiated α-MHC ES cells). Click here for file

Additional data file 10 Provided are probe sets both differentially regulated by puromycin treatment in 15-day-old β-actin EBs (twofold, Student's *t*-test *P *value < 0.01) and transcripts upregulated in α-MHC^+ ^cells (intersection of upregulation in the α-MHC^+ ^cardiomyocytes [twofold, Student's *t*-test *P *value < 0.01] compared with the control cells in the 15-day-old EBs and in the undifferentiated α-MHC ES cells). Click here for file

Additional data file 11 Provides is a list of probe sets differentially regulated in the puromycin treated 15-day-old β-actin^+ ^EBs. Click here for file

Additional data file 12 Provided is the raw data for the α-MHC experiments. Click here for file

Additional data file 13 Provided is the raw data for the α-MHC experiments. Click here for file

Additional data file 14 Provided is the raw data for the β-actin control experiments. Click here for file

## Figures and Tables

**Table 1:** Functional annotations enriched among transcripts that are upregulated in α-MHC positive cells

Category	Term	Count	P value
GOTERM_MF_5	Hydrogen ion transporter activity	30	9.49 × e^-17^
GOTERM_MF_5	NADH dehydrogenase (quinone) activity	17	6.70 × e^-14^
GOTERM_MF_5	NADH dehydrogenase (ubiquinone) activity	17	6.70 × e^-14^
GOTERM_MF_5	Sodium ion transporter activity	17	6.70 × e^-14^
KEGG_PATHWAY	Oxidative phosphorylation (*Mus musculus*)	25	8.59 × e^-12^
GOTERM_CC_5	Myofibril	16	1.36 × e^-10^
GOTERM_CC_5	Mitochondrion	61	7.52 × e^-09^
GOTERM_CC_5	Striated muscle thin filament	8	4.12 × e^-07^
GOTERM_BP_5	Cation transport	30	7.59 × e^-06^
GOTERM_CC_5	Mitochondrial membrane	21	2.91 × e^-05^
GOTERM_CC_5	Mitochondrial inner membrane	18	1.21 × e^-04^
GOTERM_MF_5	Voltage-gated ion channel activity	14	1.75 × e^-04^
GOTERM_BP_5	Coenzyme metabolism	13	2.59 × e^-04^
GOTERM_BP_5	Ion transport	35	3.21 × e^-04^
GOTERM_CC_5	Mitochondrial electron transport chain	10	3.40 × e^-04^
GOTERM_BP_5	Cytoskeleton organization and biogenesis	24	5.84 × e^-04^
GOTERM_CC_5	Actin cytoskeleton	19	5.85 × e^-04^
GOTERM_BP_5	Electron transport	23	1.41 × e^-03^
GOTERM_CC_5	Cytoskeleton	44	1.46 × e^-03^
GOTERM_MF_5	ATPase activity, coupled to transmembrane movement of ions, phosphorylative mechanism	9	1.70 × e^-03^
GOTERM_CC_5	Sodium	4	2.39 × e^-03^
KEGG_PATHWAY	Calcium signaling pathway (*Mus musculus*)	17	2.42 × e^-03^
GOTERM_BP_5	Proton transport	7	2.92 × e^-03^
GOTERM_MF_5	Sodium	4	5.26 × e^-03^
GOTERM_BP_5	Phosphate metabolism	33	7.20 × e^-03^
GOTERM_BP_5	Oxidative phosphorylation	7	7.63 × e^-03^
GOTERM_BP_5	Vesicle-mediated transport	16	7.96 × e^-03^
GOTERM_BP_5	Hydrogen transport	7	8.19 × e^-03^
GOTERM_MF_5	Cytochrome *c *oxidase activity	5	9.38 × e^-03^
GOTERM_MF_5	ATPase activity, coupled to transmembrane movement of substances	10	1.24 × e^-02^
GOTERM_BP_5	Negative regulation of signal transduction	5	1.55 × e^-02^
GOTERM_BP_5	Negative regulation of cell organization and biogenesis	4	1.56 × e^-02^
GOTERM_BP_5	Co-factor biosynthesis	8	1.62 × e^-02^
GOTERM_CC_5	Myosin	7	1.75 × e^-02^
GOTERM_BP_5	Cell growth	7	2.03 × e^-02^
GOTERM_BP_5	Regulation of cell size	7	2.36 × e^-02^
GOTERM_BP_5	Negative regulation of Wnt receptor signaling pathway	3	2.55 × e^-02^
GOTERM_MF_5	Calcium	3	2.69 × e^-02^
GOTERM_BP_5	Enzyme-linked receptor protein signaling pathway	11	3.03 × e^-02^
GOTERM_BP_5	Nucleotide biosynthesis	8	3.53 × e^-02^
GOTERM_MF_5	Protein kinase activity	25	3.66 × e^-02^
GOTERM_BP_5	Cellular protein metabolism	89	3.88 × e^-02^
GOTERM_BP_5	Negative regulation of nucleobase, nucleoside, nucleotide, and nucleic acid metabolism	9	4.10 × e^-02^
GOTERM_BP_5	Fatty acid metabolism	8	4.71 × e^-02^
GOTERM_BP_5	Intracellular receptor-mediated signaling pathway	3	5.47 × e^-02^
GOTERM_MF_5	Protein phosphatase inhibitor activity	3	5.77 × e^-02^
GOTERM_BP_5	Regulation of Wnt receptor signaling pathway	3	6.15 × e^-02^
GOTERM_BP_5	Response to temperature	4	6.33 × e^-02^
GOTERM_BP_5	Gas transport	3	6.86 × e^-02^
GOTERM_BP_5	Oxygen transport	3	6.86 × e^-02^
BIOCARTA	P38 MAPK signaling pathway (*Mus musculus*)	5	7.68 × e^-02^
GOTERM_MF_5	Arsenate reductase (glutaredoxin) activity	2	7.68 × e^-02^
GOTERM_MF_5	Hydrogen-exporting ATPase activity, phosphorylative mechanism	4	7.72 × e^-02^
GOTERM_MF_5	Microtubule binding	4	7.72 × e^-02^
GOTERM_BP_5	Apoptosis	17	7.90 × e^-02^
GOTERM_MF_5	Transcription coactivator activity	5	8.72 × e^-02^
GOTERM_BP_5	Endocytosis	8	8.94 × e^-02^
GOTERM_BP_5	Nucleotide metabolism	9	9.01 × e^-02^
GOTERM_BP_5	Secretory pathway	7	9.20 × e^-02^
GOTERM_BP_5	Cellular lipid metabolism	15	9.71 × e^-02^
GOTERM_MF_5	Transcription corepressor activity	4	9.75 × e^-02^
GOTERM_CC_5	Adherens junction	5	9.84 × e^-02^
GOTERM_BP_5	Second messenger mediated signaling	6	9.87 × e^-02^

Functional annotations that are enriched among transcripts upregulated in α-myosin heavy chain (MHC) positive cells (intersection of upregulation in the 15-day-old α-MHC^+ ^cardiomyocytes [twofold, Student's *t*-test *P *value < 0.01] as compared with the control cells in the 15-day-old embryoid bodies and compared with the undifferentiated α-MHC embryonic stem cells). 'Count' refers to the number of transcripts in the respective category; the '*P *value' column shows the value of Fisher's exact *t*-test, as used by DAVID to measure the enrichment in annotation terms. GO, Gene Ontology; KEGG, Kyoto Encyclopedia of Genes and Genomes; MAPK, mitogen-activated protein kinase.

**Table 2:** Functional annotations enriched among transcripts that are downregulated in α-MHC positive cells

Category	Term	Count	P value
GOTERM_BP_5	DNA metabolism	76	3.17 × e^-24^
GOTERM_BP_5	M phase	41	1.79 × e^-23^
GOTERM_BP_5	DNA replication	35	2.91 × e^-19^
GOTERM_BP_5	Mitotic cell cycle	35	1.92 × e^-17^
KEGG_PATHWAY	Cell cycle	32	7.70 × e^-17^
GOTERM_MF_5	ATP binding	101	3.13 × e^-15^
GOTERM_CC_5	Chromosome	46	9.39 × e^-13^
GOTERM_BP_5	DNA repair	27	2.88 × e^-10^
GOTERM_BP_5	Regulation of cell cycle	40	7.18 × e^-10^
GOTERM_CC_5	Chromosome, pericentric region	15	2.39 × e^-09^
GOTERM_BP_5	RNA processing	32	5.21 × e^-09^
GOTERM_BP_5	mRNA metabolism	24	1.28 × e^-08^
GOTERM_BP_5	RNA metabolism	37	1.29 × e^-08^
GOTERM_CC_5	Nucleus	229	2.04 × e^-07^
GOTERM_MF_5	Exonuclease activity	12	9.96 × e^-07^
GOTERM_CC_5	Kinetochore	9	1.68 × e^-06^
GOTERM_CC_5	Replication fork	9	1.68 × e^-06^
GOTERM_CC_5	Condensed chromosome	12	3.78 × e^-06^
GOTERM_CC_5	Replisome	8	8.86 × e^-06^
GOTERM_MF_5	Pyrophosphatase activity	40	1.85 × e^-05^
GOTERM_BP_5	Regulation of DNA metabolism	6	4.69 × e^-05^
KEGG_PATHWAY	Pyrimidine metabolism	14	4.94 × e^-05^
GOTERM_CC_5	Spindle	13	8.62 × e^-05^
GOTERM_BP_5	DNA recombination	11	1.44 × e^-04^
GOTERM_BP_5	DNA damage response, signal transduction	6	3.13 × e^-04^
GOTERM_BP_5	Coenzyme metabolism	15	8.57 × e^-04^
GOTERM_BP_5	Carboxylic acid metabolism	27	1.04 × e^-03^
GOTERM_BP_5	Nuclear transport	11	1.07 × e^-03^
GOTERM_CC_5	Nucleolus	16	1.22 × e^-03^
GOTERM_BP_5	Amine biosynthesis	9	1.23 × e^-03^
GOTERM_BP_5	Amino acid metabolism	16	2.25 × e^-03^
GOTERM_CC_5	Microtubule cytoskeleton	24	3.62 × e^-03^
GOTERM_BP_5	Folic acid and derivative metabolism	4	3.75 × e^-03^
GOTERM_BP_5	Nucleocytoplasmic transport	11	4.02 × e^-03^
GOTERM_BP_5	Cellular protein metabolism	135	4.25 × e^-03^
GOTERM_BP_5	Cytoskeleton organization and biogenesis	28	5.66 × e^-03^
KEGG_PATHWAY	Purine metabolism	14	6.33 × e^-03^
KEGG_PATHWAY	Glycine, serine and threonine metabolism	7	1.01 × e^-02^
KEGG_PATHWAY	DNA polymerase	6	1.10 × e^-02^
GOTERM_BP_5	Dna packaging	15	1.16 × e^-02^
GOTERM_CC_5	Nuclear chromosome	8	1.17 × e^-02^
GOTERM_CC_5	Spindle pole	8	1.17 × e^-02^
GOTERM_MF_5	S-adenosylmethionine-dependent methyltransferase activity	9	1.24 × e^-02^
BIOCARTA	Cell cycle: G2/M checkpoint	7	1.33 × e^-02^
GOTERM_BP_5	Chromosome organization and biogenesis	19	1.36 × e^-02^
BIOCARTA	Cell cycle: G1/S check point	7	1.59 × e^-02^
GOTERM_CC_5	Delta DNA polymerase complex	3	1.59 × e^-02^
BIOCARTA	Role of Ran in mitotic spindle regulation	5	1.60 × e^-02^
GOTERM_BP_5	DNA modification	6	1.64 × e^-02^
GOTERM_BP_5	Phosphate metabolism	43	1.65 × e^-02^
GOTERM_BP_5	Determination of left/right symmetry	4	1.72 × e^-02^
GOTERM_BP_5	Sulfur amino acid metabolism	4	1.72 × e^-02^
GOTERM_BP_5	Ribosome biogenesis and assembly	9	1.87 × e^-02^
BIOCARTA	Role of BRCA1	6	1.89 × e^-02^
GOTERM_BP_5	Response to radiation	6	1.99 × e^-02^
KEGG_PATHWAY	Methionine metabolism	4	2.03 × e^-02^
BIOCARTA	CDK regulation of DNA replication	4	2.15 × e^-02^
BIOCARTA	RB tumor suppressor/checkpoint signaling in response to DNA damage	4	2.15 × e^-02^
GOTERM_BP_5	Cellular carbohydrate metabolism	16	2.21 × e^-02^
GOTERM_BP_5	rRNA metabolism	5	2.50 × e^-02^
GOTERM_BP_5	Nucleic acid transport	5	2.78 × e^-02^
GOTERM_BP_5	RNA transport	5	2.78 × e^-02^
GOTERM_CC_5	Centrosome	6	2.94 × e^-02^
BIOCARTA	E2F1 destruction pathway	4	2.96 × e^-02^
BIOCARTA	Sonic Hedgehog (SHH) receptor Ptc1 regulates cell cycle	4	2.96 × e^-02^
GOTERM_MF_5	Amino acid permease activity	3	3.17 × e^-02^
KEGG_PATHWAY	One carbon pool by folate	4	3.24 × e^-02^
GOTERM_CC_5	Nuclear pore	6	3.30 × e^-02^
GOTERM_BP_5	Heparan sulfate proteoglycan metabolism	3	3.40 × e^-02^
GOTERM_BP_5	Nucleotide metabolism	13	3.93 × e^-02^
GOTERM_MF_5	Glycine hydroxymethyltransferase activity	3	3.97 × e^-02^
GOTERM_MF_5	Protein kinase activity	32	4.12 × e^-02^
GOTERM_BP_5	Nucleobase, nucleoside, nucleotide and nucleic acid transport	5	4.51 × e^-02^
GOTERM_BP_5	Positive regulation of programmed cell death	10	4.61 × e^-02^
GOTERM_MF_5	DNA-directed DNA polymerase activity	4	4.87 × e^-02^
BIOCARTA	Activation of Src by protein-tyrosine phosphatase alpha	3	5.41 × e^-02^
BIOCARTA	Cyclins and cell cycle regulation	6	5.41 × e^-02^
GOTERM_BP_5	Steroid metabolism	9	5.54 × e^-02^
GOTERM_MF_5	Pre-mRNA splicing factor activity	5	6.43 × e^-02^
GOTERM_MF_5	Methylene-tetrahydrofolate dehydrogenase activity	2	7.11 × e^-02^
GOTERM_BP_5	Regulation of cell migration	5	7.20 × e^-02^
BIOCARTA	Cdc25 and chk1 regulatory pathway in response to DNA damage	3	7.55 × e^-02^
GOTERM_CC_5	Chromatin	13	8.01 × e^-02^
GOTERM_BP_5	Regulation of apoptosis	16	8.13 × e^-02^
GOTERM_CC_5	Nuclear membrane	9	8.33 × e^-02^
GOTERM_BP_5	Nucleotide biosynthesis	9	8.59 × e^-02^
GOTERM_CC_5	Nuclear body	4	8.81 × e^-02^
GOTERM_BP_5	Regulation of programmed cell death	16	8.95 × e^-02^
KEGG_PATHWAY	Urea cycle and metabolism of amino groups	4	9.73 × e^-02^
BIOCARTA	Regulation of p27 phosphorylation during cell cycle progression	4	9.84 × e^-02^
GOTERM_CC_5	Heterochromatin	4	9.89 × e^-02^

Functional annotations that are enriched among transcripts downregulated in myosin heavy chain (MHC) positive cells (intersection of down-regulation in the 15-day-old α-MHC^+ ^cardiomyocytes [twofold, Student's *t*-test *P *value < 0.01] compared with the control cells in the 15-day-old embryoid bodies and compared with the undifferentiated α-MHC embryonic stem cells). 'Count' refers to the number of transcripts in the respective category; the '*P *value' column shows the value of Fisher's exact *t*-test, as used by DAVID to measure the enrichment in annotation terms. GO, Gene Ontology; KEGG, Kyoto Encyclopedia of Genes and Genomes; MAPK, mitogen-activated protein kinase
