# Supplementary material for: Global transcriptome analysis of murine embryonic stem cell-derived cardiomyocytes
Source: Genome Biol. 2007 Apr 11;8(4):R56. doi: 10.1186/gb-2007-8-4-r56 (PMC1896009; doi:10.1186/gb-2007-8-4-r56)
Supplement: Additional data file 3 — Summarized are the RT-PCR conditions and primers used for the RT-PCR experiments. [file gb-2007-8-4-r56-S3.doc]

**Additional data file 3:**

| **gene** | **NCBI Accession** | **forward primer** | **reverse primer** | **amplicon size (bp)** | **melting**  **point (°C)** |
| --- | --- | --- | --- | --- | --- |
| AFP | NM_007423 | ccagaacctgccgagagttgc | gccttcaggtttgacgccatt | 552 | 60 |
| **α-MHC** | BC110700. | gatggcacagaagatgctga | ctgccccttggtgacatact | 120 | 60 |
| **Cardiac Troponin T** | NM_011619 | gaggaggtggtggaggagta | ggcttcttcatcaggaccaa | 150 | 60 |
| **Mef2c** | NM_025282 | agcactgacatggataaggtgtt | ggtgagtgcataagaggagtcag | 150 | 60 |
| **Nkx.2.5** | NM_008700. | ccaaagaccctcgggcggata | gcgcagctgtagccgggactg | 493 | 60 |
| **Myocardin** | AY303755 | tcctggctcagaaagtgaca | cggttcttactgtcacccaaa | 147 | 60 |
| **MLC-2v** | NM_010861.2 | aaagaggctccaggtccaat | tcagccttcagtgacccttt | 140 | 60 |
| **GAPDH** | BC085275. | cagcaaggacactgagcaag | gggtgcagcgaactttattg | 156 | 60 |

Primer sequences for RT-PCR analysis of total RNA isolated from ES cells and EBs
