# Supplementary material for: Global transcriptome analysis of murine embryonic stem cell-derived cardiomyocytes
Source: Genome Biol. 2007 Apr 11;8(4):R56. doi: 10.1186/gb-2007-8-4-r56 (PMC1896009; doi:10.1186/gb-2007-8-4-r56)
Supplement: Additional data file 4 — Provided are lists of probe sets for the subclusters A and B, as identified in the hierarchical clustering of probe sets upregulated in α-MHC+ cells (Figure 5). [file gb-2007-8-4-r56-S4.doc]

**Additional data file 4.**

### Subcluster A

| **Probe sets** | Symbol | Title | **fc d0**  **vs. d15** | **fc d0**  **vs. αMHC^+^** | **fc d15**  **vs. αMHC^+^** |
| --- | --- | --- | --- | --- | --- |
| 1418095_at | Smpx | small muscle protein, X-linked | 10.4 | 205.4 | 19.8 |
| 1427446_s_at | Ttn | titin | 10.4 | 178.8 | 17.2 |
| 1451801_at | Trdn | triadin | 21.0 | 309.9 | 14.7 |
| 1418769_at | Myoz2 | myozenin 2 | 14.2 | 181.5 | 12.8 |
| 1423359_at | Pln | phospholamban | 11.7 | 147.4 | 12.6 |
| 1448327_at | Actn2 | actinin alpha 2 | 16.4 | 204.1 | 12.4 |
| 1417701_at | Ppp1r14c | protein phosphatase 1, regulatory (inhibitor) subunit 14c | 10.3 | 124.1 | 12.1 |
| 1450952_at | Pln | phospholamban | 12.6 | 146.5 | 11.7 |
| 1447806_s_at | Stk23 | serine/threonine kinase 23 | 8.0 | 89.2 | 11.2 |
| 1437197_at | Sorbs2 | sorbin and SH3 domain containing 2 | 9.7 | 100.1 | 10.4 |
| 1426283_at | Hnt | neurotrimin | 7.5 | 75.8 | 10.1 |
| 1422754_at | Tmod1 | tropomodulin 1 | 5.8 | 57.1 | 9.8 |
| 1425978_at | Myocd | myocardin | 12.2 | 116.4 | 9.6 |
| 1457633_x_at | Cox6a2 | cytochrome c oxidase, subunit VI a, polypeptide 2 | 9.8 | 93.7 | 9.6 |
| 1447713_at | Tpm1 | tropomyosin 1, alpha | 6.2 | 58.3 | 9.4 |
| 1460332_at | Pln | phospholamban | 43.0 | 395.9 | 9.2 |
| 1455607_at | Rspo3 | R-spondin 3 homolog (Xenopus laevis) | 18.3 | 167.4 | 9.2 |
| 1421374_a_at | Fxyd1 | FXYD domain-containing ion transport regulator 1 | 7.0 | 62.8 | 9.0 |
| 1441165_s_at | Clstn2 | calsyntenin 2 | 5.9 | 50.4 | 8.6 |
| 1422536_at | Tnni3 | troponin I, cardiac | 12.9 | 110.4 | 8.6 |
| 1422644_at | Sh3bgr | Putative SH3BGR protein (SH3BGR gene) | 42.2 | 360.4 | 8.5 |
| 1449240_at | Gsbs | G substrate | 6.1 | 51.0 | 8.4 |
| 1433783_at | Ldb3 | LIM domain binding 3 | 16.0 | 132.7 | 8.3 |
| 1448747_at | Fbxo32 | F-box only protein 32 | 6.4 | 52.9 | 8.3 |
| 1447657_s_at | Synpo2l | synaptopodin 2-like | 6.2 | 50.9 | 8.2 |
| 1460336_at | Ppargc1a | peroxisome proliferative activated receptor, gamma, coactivator 1 alpha | 20.7 | 168.9 | 8.2 |
| 1429783_at | Pdlim5 | PDZ and LIM domain 5 | 8.1 | 64.9 | 8.0 |
| 1447927_at | Mpa2l | macrophage activation 2 like | 10.9 | 86.0 | 7.9 |
| 1426282_at | Hnt | neurotrimin | 5.7 | 44.4 | 7.8 |
| 1458492_x_at | Hnt | neurotrimin | 4.9 | 37.9 | 7.7 |
| 1428266_at | Myl3 | myosin, light polypeptide 3 | 20.0 | 152.6 | 7.6 |
| 1428444_at | Asb2 | ankyrin repeat and SOCS box-containing protein 2 | 12.5 | 93.8 | 7.5 |
| 1418373_at | Pgam2 | phosphoglycerate mutase 2 | 8.1 | 60.2 | 7.5 |
| 1438452_at | Nebl | nebulette | 22.1 | 164.6 | 7.5 |
| 1427445_a_at | Ttn | titin | 20.1 | 148.3 | 7.4 |
| 1442143_at | Tmem16d | transmembrane protein 16D (eight membrane-spanning domains) | 3.4 | 25.0 | 7.3 |
| 1418589_a_at | Mlf1 | myeloid leukemia factor 1 | 8.7 | 61.4 | 7.1 |
| 1436223_at | Itgb8 | PREDICTED: integrin beta 8 [Mus musculus], mRNA sequence | 10.6 | 73.8 | 7.0 |
| 1434100_x_at | Ppargc1a | Caspase 7, mRNA (cDNA clone MGC:5872 IMAGE:3600155) | 5.0 | 33.7 | 6.8 |
| 1437482_at | Srd5a2l2 | steroid 5 alpha-reductase 2-like 2 | 11.1 | 74.7 | 6.8 |
| 1434099_at | Ppargc1a | Caspase 7, mRNA (cDNA clone MGC:5872 IMAGE:3600155) | 9.7 | 65.0 | 6.7 |
| 1428662_a_at | Hod | homeobox only domain | 6.9 | 45.8 | 6.7 |
| 1418030_at | Slco3a1 | solute carrier organic anion transporter family, member 3a1 | 5.9 | 39.3 | 6.7 |
| 1435649_at | Nexn | nexilin | 21.7 | 143.6 | 6.6 |
| 1455164_at | Cdgap | Cdc42 GTPase-activating protein | 6.4 | 42.0 | 6.6 |
| 1452922_at | Ppp1r3d | protein phosphatase 1, regulatory subunit 3D | 6.0 | 39.0 | 6.5 |
| 1417607_at | Cox6a2 | cytochrome c oxidase, subunit VI a, polypeptide 2 | 5.7 | 36.3 | 6.4 |
| 1436939_at | Unc45b | cardiomyopathy associated 4 | 34.1 | 209.2 | 6.1 |
| 1417673_at | Grb14 | growth factor receptor bound protein 14 | 5.2 | 30.3 | 5.9 |
| 1450268_at | Fign | fidgetin | 3.3 | 19.1 | 5.8 |
| 1426285_at | Lama2 | laminin, alpha 2 | 5.2 | 29.4 | 5.6 |
| 1440862_at | Gpr178 | G protein-coupled receptor 178 | 7.1 | 39.2 | 5.5 |
| 1434572_at | Hdac9 | histone deacetylase 9 | 5.8 | 31.6 | 5.5 |
| 1426708_at | Antxr2 | anthrax toxin receptor 2 | 50.0 | 270.7 | 5.4 |
| 1452353_at | Gpr155 | G protein-coupled receptor 155 | 3.0 | 15.4 | 5.2 |
| 1427164_at | Il13ra1 | interleukin 13 receptor, alpha 1 | 5.8 | 29.5 | 5.1 |
| 1427186_a_at | Mef2a | myocyte enhancer factor 2A | 9.3 | 46.2 | 5.0 |
| 1447903_x_at | Ap1s2 | adaptor-related protein complex 1, sigma 2 subunit | 4.5 | 21.6 | 4.8 |
| 1423557_at | Ifngr2 | interferon gamma receptor 2 | 4.8 | 23.3 | 4.8 |
| 1452657_at | Ap1s2 | adaptor-related protein complex 1, sigma 2 subunit | 4.5 | 21.4 | 4.8 |
| 1421252_a_at | Mef2a | myocyte enhancer factor 2A | 6.6 | 31.6 | 4.8 |
| 1450621_a_at | Hbb-y | hemoglobin Y, beta-like embryonic chain | 9.0 | 42.7 | 4.7 |
| 1418497_at | Fgf13 | fibroblast growth factor 13 | 10.7 | 50.6 | 4.7 |
| 1422580_at | Myl4 | myosin, light polypeptide 4 | 21.4 | 99.8 | 4.7 |
| 1460318_at | Csrp3 | cysteine and glycine-rich protein 3 | 9.4 | 42.8 | 4.6 |
| 1455901_at | Chpt1 | choline phosphotransferase 1 | 3.6 | 16.4 | 4.5 |
| 1451342_at | Spon1 | spondin 1, (f-spondin) extracellular matrix protein | 22.6 | 100.6 | 4.5 |
| 1452347_at | Mef2a | myocyte enhancer factor 2A | 8.2 | 36.5 | 4.4 |
| 1448394_at | Myl2 | myosin, light polypeptide 2, regulatory, cardiac, slow | 77.2 | 340.3 | 4.4 |
| 1436650_at | Filip1 | filamin A interacting protein 1 | 7.1 | 31.0 | 4.4 |
| 1425534_at | Stau2 | staufen (RNA binding protein) homolog 2 (Drosophila) | 4.5 | 19.3 | 4.3 |
| 1434820_s_at | Pkig | protein kinase inhibitor, gamma | 3.1 | 13.4 | 4.2 |
| 1433453_a_at | Abtb2 | ankyrin repeat and BTB (POZ) domain containing 2 | 3.4 | 14.3 | 4.2 |
| 1434369_a_at | Cryab | crystallin, alpha B | 44.2 | 183.9 | 4.2 |
| 1448669_at | Dkk3 | dickkopf homolog 3 (Xenopus laevis) | 13.9 | 57.2 | 4.1 |
| 1448554_s_at | Myh6 | myosin, heavy polypeptide 6, cardiac muscle, alpha | 104.6 | 421.6 | 4.0 |
| 1429622_at | Cand2 | cullin-associated and neddylation-dissociated 2 (putative) | 2.4 | 9.6 | 4.0 |
| 1438251_x_at | Htra1 | HtrA serine peptidase 1 | 9.6 | 37.5 | 3.9 |
| 1456139_at | Air | antisense Igf2r RNA | 2.9 | 11.4 | 3.9 |
| 1450813_a_at | Tnni1 | troponin I, skeletal, slow 1 | 69.9 | 273.6 | 3.9 |
| 1427768_s_at | Myl3 | myosin, light polypeptide 3 | 228.7 | 892.7 | 3.9 |
| 1435910_at | Fads3 | Fatty acid desaturase 3 (Fads3), mRNA | 2.9 | 11.1 | 3.9 |
| 1427769_x_at | Myl3 | myosin, light polypeptide 3 | 46.9 | 180.2 | 3.8 |
| 1452380_at | Epha7 | Eph receptor A7 | 5.5 | 20.7 | 3.8 |
| 1416749_at | Htra1 | HtrA serine peptidase 1 | 11.8 | 44.6 | 3.8 |
| 1453351_at | Tbx20 | T-box 20 | 26.8 | 99.8 | 3.7 |
| 1436717_x_at | Hbb-y | hemoglobin Y, beta-like embryonic chain | 249.9 | 928.2 | 3.7 |
| 1436823_x_at | Hbb-y | hemoglobin Y, beta-like embryonic chain | 285.1 | 1049.2 | 3.7 |
| 1438183_x_at | Sord | sorbitol dehydrogenase 1 | 4.2 | 15.3 | 3.7 |
| 1422562_at | Rrad | Ras-related associated with diabetes | 12.1 | 44.3 | 3.7 |
| 1436853_a_at | Snca | synuclein, alpha | 6.1 | 22.2 | 3.7 |
| 1452363_a_at | Atp2a2 | ATPase, Ca++ transporting, cardiac muscle, slow twitch 2 | 6.7 | 24.5 | 3.7 |
| 1447854_s_at | Hist2h2be | histone 2, H2be | 2.4 | 8.8 | 3.6 |
| 1455078_at | Hsp90aa1 | Heat shock protein 90kDa alpha (cytosolic), class A member 1 | 3.3 | 12.0 | 3.6 |
| 1439093_at | Hspa4l | heat shock protein 4 like | 2.6 | 9.4 | 3.6 |
| 1450732_a_at | Bicd2 | bicaudal D homolog 2 (Drosophila) | 3.2 | 11.3 | 3.6 |
| 1457589_at | Fat3 | FAT tumor suppressor homolog 3 (Drosophila) | 4.0 | 13.9 | 3.5 |
| 1455056_at | Lmo7 | LIM domain only 7 | 3.3 | 11.4 | 3.4 |
| 1418815_at | Cdh2 | cadherin 2 | 98.4 | 329.8 | 3.4 |
| 1436042_at | Tln1 | talin 1 | 3.6 | 12.0 | 3.3 |
| 1454646_at | Tcp11l2 | t-complex 11 (mouse) like 2 | 2.5 | 8.4 | 3.3 |
| 1434272_at | Cpeb2 | cytoplasmic polyadenylation element binding protein 2 | 8.0 | 26.4 | 3.3 |
| 1457038_at | Frem2 | Fras1 related extracellular matrix protein 2 | 11.2 | 36.7 | 3.3 |
| 1449106_at | Gpx3 | glutathione peroxidase 3 | 11.2 | 36.2 | 3.2 |
| 1437990_x_at | Hbb-bh1 | hemoglobin Z, beta-like embryonic chain | 95.9 | 310.6 | 3.2 |
| 1454849_x_at | Clu | clusterin | 18.2 | 58.8 | 3.2 |
| 1437810_a_at | Hbb-bh1 | hemoglobin Z, beta-like embryonic chain | 60.3 | 194.0 | 3.2 |
| 1433768_at | Palld | palladin, cytoskeletal associated protein | 3.1 | 9.8 | 3.2 |
| 1424308_at | Slc24a3 | solute carrier family 24 (sodium/potassium/calcium exchanger), member 3 | 3.8 | 12.0 | 3.2 |
| 1424967_x_at | Tnnt2 | troponin T2, cardiac | 292.5 | 921.7 | 3.2 |
| 1419081_at | Atg10 | autophagy-related 10 (yeast) | 4.1 | 12.9 | 3.1 |
| 1418370_at | Tnnc1 | troponin C, cardiac/slow skeletal | 179.2 | 561.2 | 3.1 |
| 1418726_a_at | Tnnt2 | troponin T2, cardiac | 463.6 | 1440.8 | 3.1 |
| 1449071_at | Myl7 | myosin, light polypeptide 7, regulatory | 79.7 | 247.1 | 3.1 |
| 1420859_at | Pkia | protein kinase inhibitor, alpha | 17.2 | 53.2 | 3.1 |
| 1457637_at | Prickle1 | prickle like 1 | 4.3 | 13.4 | 3.1 |
| 1437458_x_at | Clu | clusterin | 12.6 | 38.7 | 3.1 |
| 1419255_at | Spnb2 | spectrin beta 2 | 3.3 | 9.9 | 3.0 |
| 1448826_at | Myh6 | myosin, heavy polypeptide 6, cardiac muscle, alpha | 303.3 | 916.1 | 3.0 |
| 1422710_a_at | Cacna1h | calcium channel, voltage-dependent, T type, alpha 1H subunit | 5.8 | 17.3 | 3.0 |
| 1435645_at | Mmd | monocyte to macrophage differentiation-associated | 6.7 | 19.8 | 3.0 |
| 1455267_at | Esrrg | estrogen-related receptor gamma | 2.9 | 8.4 | 2.9 |
| 1457434_s_at | Ptpla | protein tyrosine phosphatase-like (proline instead of catalytic arginine), member a | 3.8 | 11.0 | 2.9 |
| 1460337_at | Sh3kbp1 | SH3-domain kinase binding protein 1 | 7.5 | 21.8 | 2.9 |
| 1416551_at | Atp2a2 | ATPase, Ca++ transporting, cardiac muscle, slow twitch 2 | 7.6 | 21.3 | 2.8 |
| 1430561_at | Dnajb14 | DnaJ (Hsp40) homolog, subfamily B, member 14 | 3.5 | 9.6 | 2.8 |
| 1453851_a_at | Gadd45g | growth arrest and DNA-damage-inducible 45 gamma | 18.1 | 50.3 | 2.8 |
| 1450736_a_at | Hbb-bh1 | hemoglobin Z, beta-like embryonic chain | 21.7 | 59.7 | 2.8 |
| 1427185_at | Mef2a | MEF2A mRNA, partial 3' UTR sequence | 6.4 | 17.7 | 2.7 |
| 1437689_x_at | Clu | clusterin | 25.9 | 71.0 | 2.7 |
| 1428484_at | Osbpl3 | oxysterol binding protein-like 3 | 2.8 | 7.6 | 2.7 |
| 1455385_at | Exoc6 | SEC15-like 1 (S. cerevisiae) | 7.0 | 18.9 | 2.7 |
| 1434736_at | Hlf | hepatic leukemia factor | 1.7 | 4.6 | 2.6 |
| 1424349_a_at | Lpgat1 | lysophosphatidylglycerol acyltransferase 1 | 2.9 | 7.8 | 2.6 |
| 1451542_at | Ssbp2 | single-stranded DNA binding protein 2 | 8.2 | 21.5 | 2.6 |
| 1451991_at | Epha7 | Eph receptor A7 | 23.9 | 62.4 | 2.6 |
| 1449583_at | Pcdhb20 | protocadherin beta 20 | 6.6 | 17.2 | 2.6 |
| 1418318_at | Rnf128 | ring finger protein 128 | 2.4 | 6.2 | 2.6 |
| 1424261_at | Zfp672 | zinc finger protein 672 | 8.7 | 22.3 | 2.6 |
| 1447720_x_at | Prkaca | Protein kinase, cAMP dependent, catalytic, alpha, mRNA | 2.1 | 5.3 | 2.5 |
| 1425270_at | Kif1b | kinesin family member 1B | 2.3 | 5.7 | 2.5 |
| 1452114_s_at | Igfbp5 | insulin-like growth factor binding protein 5 | 113.3 | 283.6 | 2.5 |
| 1452973_at | Ppm1k | protein phosphatase 1K (PP2C domain containing) | 2.1 | 5.1 | 2.5 |
| 1437462_x_at | Mmp15 | Transcribed locus | 2.6 | 6.3 | 2.5 |
| 1438294_at | Atxn1 | spinocerebellar ataxia 1 homolog (human) | 6.8 | 16.5 | 2.4 |
| 1424755_at | Hip1 | huntingtin interacting protein 1 | 2.9 | 7.2 | 2.4 |
| 1421088_at | Gpc4 | glypican 4 | 2.7 | 6.4 | 2.4 |
| 1429005_at | Mfhas1 | malignant fibrous histiocytoma amplified sequence 1 | 16.8 | 40.3 | 2.4 |
| 1449036_at | Rnf128 | ring finger protein 128 | 2.4 | 5.7 | 2.4 |
| 1440431_at | Meis1 | Myeloid ecotropic viral integration site 1, mRNA | 6.7 | 15.9 | 2.4 |
| 1451177_at | Dnajb4 | DnaJ (Hsp40) homolog, subfamily B, member 4 | 6.4 | 15.3 | 2.4 |
| 1421027_a_at | Mef2c | myocyte enhancer factor 2C | 31.7 | 75.1 | 2.4 |
| 1418960_at | Phf20l1 | PHD finger protein 20-like 1 | 2.4 | 5.7 | 2.4 |
| 1418018_at | Cpd | carboxypeptidase D | 11.0 | 25.8 | 2.4 |
| 1425918_at | Egln3 | EGL nine homolog 3 (C. elegans) | 6.7 | 15.7 | 2.3 |
| 1434909_at | Rragd | Ras-related GTP binding D | 4.7 | 10.9 | 2.3 |
| 1420618_at | Cpeb4 | cytoplasmic polyadenylation element binding protein 4 | 3.1 | 7.1 | 2.3 |
| 1457229_at | Gpr173 | G-protein coupled receptor 173 | 2.1 | 4.8 | 2.3 |
| 1419668_at | Sgcb | sarcoglycan, beta (dystrophin-associated glycoprotein) | 5.2 | 11.9 | 2.3 |
| 1442051_at | Hist2h3c1 | histone 2, H3c1 | 1.8 | 4.0 | 2.3 |
| 1421127_at | Tmem42 | transmembrane protein 42 | 2.4 | 5.5 | 2.3 |
| 1452251_at | Nbea | neurobeachin | 8.8 | 19.9 | 2.3 |
| 1436600_at | Tnrc9 | trinucleotide repeat containing 9 | 17.7 | 39.8 | 2.2 |
| 1440384_at | Tmcc1 | transmembrane and coiled coil domains 1 | 1.8 | 4.1 | 2.2 |
| 1451474_a_at | Parp8 | poly (ADP-ribose) polymerase family, member 8 | 2.3 | 5.2 | 2.2 |
| 1435493_at | Dsp | desmoplakin | 5.1 | 11.3 | 2.2 |
| 1439382_x_at | Ddr1 | discoidin domain receptor family, member 1 | 1.8 | 4.1 | 2.2 |
| 1451642_at | Kif1b | kinesin family member 1B | 3.0 | 6.5 | 2.2 |
| 1416632_at | Mod1 | malic enzyme, supernatant | 3.1 | 6.8 | 2.2 |
| 1456226_x_at | Ddr1 | discoidin domain receptor family, member 1 | 3.5 | 7.8 | 2.2 |
| 1433722_at | Akap13 | A kinase (PRKA) anchor protein 13 | 2.5 | 5.6 | 2.2 |
| 1420617_at | Cpeb4 | cytoplasmic polyadenylation element binding protein 4 | 3.1 | 6.8 | 2.2 |
| 1435820_x_at | Ddr1 | discoidin domain receptor family, member 1 | 3.4 | 7.4 | 2.2 |
| 1426405_at | Rnf11 | ring finger protein 11 | 1.7 | 3.7 | 2.2 |
| 1450791_at | Nppb | natriuretic peptide precursor type B | 1.7 | 3.7 | 2.2 |
| 1424594_at | Lgals7 | Lgals7- lectin, galactose binding, soluble 7 | 28.5 | 62.3 | 2.2 |
| 1449630_s_at | Mark1 | MAP/microtubule affinity-regulating kinase 1 | 5.6 | 12.2 | 2.2 |
| 1443897_at | Ddit3 | DNA-damage inducible transcript 3 | 1.6 | 3.5 | 2.2 |
| 1428405_at | Hcfc1r1 | host cell factor C1 regulator 1 (XPO1-dependent) | 2.8 | 6.0 | 2.1 |
| 1456482_at | Pik3r3 | phosphatidylinositol 3 kinase, regulatory subunit, polypeptide 3 (p55) | 3.1 | 6.7 | 2.1 |
| 1424394_at | Selm | selenoprotein M | 4.5 | 9.6 | 2.1 |
| 1435020_at | Sdccag1 | Serologically defined colon cancer antigen 1 (Sdccag1), transcript variant 1, mRNA | 2.3 | 4.8 | 2.1 |
| 1456022_at | Hipk2 | homeodomain interacting protein kinase 2 | 2.7 | 5.7 | 2.1 |
| 1455286_at | Btbd1 | BTB (POZ) domain containing 1 | 1.9 | 4.0 | 2.1 |
| 1437351_at | Cxxc4 | CXXC finger 4 | 4.9 | 10.3 | 2.1 |
| 1449459_s_at | Asb13 | ankyrin repeat and SOCS box-containing protein 13 | 5.7 | 11.9 | 2.1 |
| 1434683_at | Cutl1 | Cut-like 1 (Drosophila), transcript variant 2, mRNA | 3.8 | 7.9 | 2.1 |
| 1447812_x_at | Flnc | filamin C, gamma (actin binding protein 280) | 8.1 | 16.6 | 2.1 |
| 1428343_at | Rcor3 | REST corepressor 3 | 2.5 | 5.2 | 2.1 |
| 1416578_at | Rbx1 | ring-box 1 | 1.7 | 3.4 | 2.1 |
| 1438930_s_at | Mecp2 | methyl CpG binding protein 2 | 3.7 | 7.5 | 2.0 |
| 1419047_at | Pcnx | pecanex homolog (Drosophila) | 2.0 | 4.0 | 2.0 |
| 1436895_at | Centd1 | centaurin, delta 1 | 2.2 | 4.4 | 2.0 |
| 1435023_at | Itsn2 | Intersectin 2 (Itsn2), mRNA | 2.2 | 4.4 | 2.0 |
| 1426461_at | Ugp2 | UDP-glucose pyrophosphorylase 2 | 3.3 | 6.6 | 2.0 |
| 1438211_s_at | Dbp | D site albumin promoter binding protein | 2.6 | 5.3 | 2.0 |

**Subcluster B**

| Probe sets | Symbol | Title | **fc d0**  **vs. d15** | **fc d0**  **vs. αMHC^+^** | **fc d15**  **vs. αMHC^+^** |
| --- | --- | --- | --- | --- | --- |
| 1426143_at | Trdn | triadin | 3.4 | 152.2 | 44.9 |
| 1426144_x_at | Trdn | triadin | 3.0 | 127.7 | 42.8 |
| 1444083_at | Ttn | titin | 2.3 | 95.9 | 41.6 |
| 1417729_at | Myh6 | myosin, heavy polypeptide 6, cardiac muscle, alpha | 2.1 | 81.4 | 37.9 |
| 1426142_a_at | Trdn | triadin | 5.7 | 185.7 | 32.5 |
| 1450917_at | Myom2 | myomesin 2 | 1.3 | 41.9 | 31.4 |
| 1443799_at | Ppp1r14c | Protein phosphatase 1, regulatory (inhibitor) subunit 14c (Ppp1r14c), mRNA | 2.1 | 56.2 | 26.4 |
| 1441679_at | Cacna1c | Calcium channel, voltage-dependent, L type, alpha 1C subunit (Cacna1c), mRNA | 1.9 | 50.5 | 25.9 |
| 1457435_x_at | Myom2 | myomesin 2 | 2.5 | 64.2 | 25.5 |
| 1436044_at | Scn7a | sodium channel, voltage-gated, type VII, alpha | 2.0 | 49.7 | 24.9 |
| 1451940_x_at | Trdn | triadin | 1.2 | 29.3 | 24.4 |
| 1436043_at | Scn7a | sodium channel, voltage-gated, type VII, alpha | 1.5 | 35.6 | 24.3 |
| 1422153_a_at | Asb11 | ankyrin repeat and SOCS box-containing protein 11 | 1.0 | 25.0 | 24.1 |
| 1441624_at | Sorbs2 | sorbin and SH3 domain containing 2 | 1.3 | 28.9 | 22.2 |
| 1451203_at | Mb | myoglobin | 7.5 | 164.1 | 21.9 |
| 1444638_at | Ttn | titin | 6.3 | 137.3 | 21.8 |
| 1448827_s_at | Myh6 | myosin, heavy polypeptide 6, cardiac muscle, alpha; myosin, heavy polypeptide 7, cardiac muscle, beta | 4.2 | 88.1 | 21.0 |
| 1439793_at | Gja3 | Gap junction membrane channel protein alpha 3 (Gja3), mRNA | 1.6 | 32.2 | 19.7 |
| 1438175_x_at | Myom2 | myomesin 2 | 2.1 | 40.7 | 19.1 |
| 1439658_at | Lmod3 | leiomodin 3 (fetal) | 1.2 | 22.6 | 18.5 |
| 1452474_a_at | Art3 | ADP-ribosyltransferase 3 | 7.2 | 129.8 | 18.1 |
| 1450193_at | Hcn1 | hyperpolarization-activated, cyclic nucleotide-gated K+ 1 | 2.2 | 39.6 | 18.0 |
| 1430286_s_at | Ppp1r14c | protein phosphatase 1, regulatory (inhibitor) subunit 14c | 5.2 | 91.2 | 17.6 |
| 1441667_s_at | Smyd1 | SET and MYND domain containing 1 | 3.6 | 63.2 | 17.3 |
| 1449218_at | Cox8b | cytochrome c oxidase, subunit VIIIb | 1.6 | 27.9 | 17.1 |
| 1417626_at | Pde4dip | upregulated during skeletal muscle growth 4 | 2.1 | 34.7 | 16.5 |
| 1418951_at | Txlnb | taxilin beta | 2.2 | 34.5 | 15.9 |
| 1452345_at | Lmod2 | leiomodin 2 (cardiac) | 2.2 | 34.1 | 15.8 |
| 1429197_s_at | Rabgap1l | RAB GTPase activating protein 1-like | 6.6 | 103.6 | 15.7 |
| 1434196_at | Dnaja4 | DnaJ (Hsp40) homolog, subfamily A, member 4 | 1.5 | 22.3 | 15.2 |
| 1456968_at | Actn2 | Actinin alpha 2 (Actn2) | 1.4 | 21.8 | 15.2 |
| 1429196_at | Rabgap1l | RAB GTPase activating protein 1-like | 7.4 | 110.7 | 15.0 |
| 1451999_at | Ldb3 | LIM domain binding 3 | 3.4 | 51.5 | 15.0 |
| 1419985_s_at | Ccdc69 | DNA segment, Chr 11, ERATO Doi 461, expressed | 1.5 | 22.2 | 14.9 |
| 1445498_at | Ryr2 | Ryanodine receptor 2, cardiac (Ryr2), mRNA | -1.2 | 12.1 | 14.8 |
| 1450123_at | Ryr2 | ryanodine receptor 2, cardiac | 4.8 | 71.8 | 14.8 |
| 1455296_at | Adcy5 | adenylate cyclase 5 | 2.7 | 39.7 | 14.7 |
| 1437675_at | Slc8a1 | solute carrier family 8 (sodium/calcium exchanger), member 1 | 5.7 | 83.5 | 14.6 |
| 1449872_at | Hspb3 | heat shock protein 3 | 1.5 | 21.9 | 14.6 |
| 1419440_at | Trim54 | tripartite motif-containing 54 | -1.2 | 12.2 | 14.4 |
| 1426134_at | Trdn | triadin | 1.2 | 17.7 | 14.4 |
| 1420362_a_at | Bik | Bcl2-interacting killer-like | 2.4 | 32.6 | 13.7 |
| 1419539_at | Irx4 | Iroquois related homeobox 4 (Drosophila) | 1.3 | 17.0 | 13.3 |
| 1447043_at | Erbb4 | v-erb-a erythroblastic leukemia viral oncogene homolog 4 [Mus musculus], mRNA sequence | 1.1 | 14.9 | 13.2 |
| 1427278_at | Rsnl2 | restin-like 2 | 1.4 | 17.2 | 12.7 |
| 1455785_at | Kcna1 | Potassium voltage-gated channel, shaker-related subfamily, member 1 | 4.0 | 50.2 | 12.6 |
| 1436731_at | Zfp533 | zinc finger protein 533 | 2.0 | 24.6 | 12.5 |
| 1454137_s_at | Hfe2 | hemochromatosis type 2 (juvenile) (human homolog) | -2.5 | 4.9 | 12.3 |
| 1443823_s_at | Atp1a2 | Transcribed locus | 5.7 | 69.1 | 12.1 |
| 1437230_at | Kcna1 | Potassium voltage-gated channel, shaker-related subfamily, member 1 | 1.8 | 21.5 | 11.7 |
| 1435551_at | Fhod3 | formin-family protein FHOS2 | -1.1 | 10.5 | 11.5 |
| 1451830_a_at | Spnb2 | spectrin beta 2 | 2.3 | 25.9 | 11.4 |
| 1444693_at | Cacnb2 | Calcium channel, voltage-dependent, beta 2 subunit | 4.0 | 44.9 | 11.4 |
| 1417889_at | Apobec2 | apolipoprotein B editing complex 2 | 4.1 | 46.3 | 11.3 |
| 1456395_at | Ppargc1a | peroxisome proliferative activated receptor, gamma, coactivator 1 alpha | 3.0 | 33.3 | 11.0 |
| 1451776_s_at | Hod | homeobox only domain | 2.5 | 26.3 | 10.7 |
| 1437797_at | Atp2a2 | ATPase, Ca++ transporting, cardiac muscle, slow twitch 2 | 1.2 | 11.6 | 9.8 |
| 1425158_at | Tbx20 | T-box 20 | 4.8 | 46.4 | 9.7 |
| 1457031_at | Fsd2 | fibronectin type III and SPRY domain containing 2 | 1.5 | 14.1 | 9.5 |
| 1416752_at | Ldb3 | LIM domain binding 3 | 2.2 | 20.4 | 9.3 |
| 1424030_at | Grhl1 | grainyhead-like 1 (Drosophila) | -1.4 | 6.6 | 9.2 |
| 1417946_at | Abhd3 | abhydrolase domain containing 3 | -2.5 | 3.7 | 9.2 |
| 1425425_a_at | Wif1 | Wnt inhibitory factor 1 | 1.9 | 17.6 | 9.1 |
| 1455372_at | Cpeb3 | Cytoplasmic polyadenylation element binding protein 3 | -1.4 | 6.3 | 9.1 |
| 1418723_at | Edg7 | endothelial differentiation, lysophosphatidic acid G-protein-coupled receptor 7 | 4.3 | 38.6 | 9.0 |
| 1436212_at | Tmem71 | ransmembrane protein 71 | 2.5 | 22.6 | 9.0 |
| 1445841_at | Lrrc39 | leucine rich repeat containing 39 | 1.2 | 10.7 | 8.9 |
| 1460591_at | Esr1 | estrogen receptor 1 (alpha) | 1.1 | 9.5 | 8.8 |
| 1447853_x_at | Kif13a | KIF13A | -1.2 | 7.0 | 8.7 |
| 1439897_at | Nebl | Nebulette (Actin-binding Z-disk protein) | 2.6 | 22.5 | 8.6 |
| 1424367_a_at | Homer2 | homer homolog 2 (Drosophila) | 3.5 | 29.9 | 8.5 |
| 1426032_at | Nfatc2 | nuclear factor of activated T-cells, cytoplasmic, calcineurin-dependent 2 | 1.0 | 8.9 | 8.5 |
| 1452478_at | Alpk2 | alpha-kinase 2 | 2.8 | 23.9 | 8.4 |
| 1422654_at | Sgca | sarcoglycan, alpha (dystrophin-associated glycoprotein) | 1.1 | 9.2 | 8.3 |
| 1434990_at | Ppm1e | protein phosphatase 1E (PP2C domain containing) | -1.3 | 6.1 | 8.2 |
| 1425275_at | Asph | aspartate-beta-hydroxylase | 1.3 | 10.4 | 8.0 |
| 1440635_at | Palld | palladin, cytoskeletal associated protein | 1.4 | 10.8 | 8.0 |
| 1435991_at | Nr3c2 | nuclear receptor subfamily 3, group C, member 2 [Mus musculus] | 1.1 | 8.1 | 7.7 |
| 1458482_at | Tnni3k | TNNI3 interacting kinase | 1.0 | 7.8 | 7.6 |
| 1425695_at | Tbx5 | T-box 5 | 2.3 | 17.5 | 7.6 |
| 1421297_a_at | Cacna1c | calcium channel, voltage-dependent, L type, alpha 1C subunit | 2.2 | 16.4 | 7.6 |
| 1436867_at | Srl | sarcalumenin | 2.6 | 19.4 | 7.5 |
| 1429718_at | Slitrk5 | SLIT and NTRK-like family, member 5 | 3.1 | 23.0 | 7.5 |
| 1419109_at | Hrc | histidine rich calcium binding protein | 3.0 | 22.8 | 7.5 |
| 1428804_at | Mfap3l | microfibrillar-associated protein 3-like | 1.4 | 10.9 | 7.5 |
| 1419606_a_at | Tnnt1 | troponin T1, skeletal, slow | 1.2 | 9.0 | 7.5 |
| 1459358_at | Pkp2 | plakophilin 2 | 1.4 | 10.2 | 7.4 |
| 1460511_at | Pkp2 | plakophilin 2 | 1.3 | 9.7 | 7.4 |
| 1447500_at | Cutl2 | Cut-like 2 (Drosophila) | 1.1 | 7.9 | 7.3 |
| 1456062_at | Nppa | natriuretic peptide precursor type A | 1.6 | 11.3 | 7.2 |
| 1417168_a_at | Usp2 | ubiquitin specific peptidase 2 | 2.5 | 18.0 | 7.2 |
| 1421425_a_at | Dscr1l1 | Down syndrome critical region gene 1-like 1 | 3.1 | 21.9 | 7.1 |
| 1455493_at | Syne1 | synaptic nuclear envelope 1 | 2.3 | 16.4 | 7.1 |
| 1422064_a_at | Zbtb20 | zinc finger and BTB domain containing 20 | 2.6 | 18.6 | 7.1 |
| 1440424_at | Tnnt2 | Troponin T2, cardiac (Tnnt2), mRNA | 1.7 | 12.1 | 7.0 |
| 1434944_at | Dmpk | dystrophia myotonica-protein kinase | 1.8 | 12.9 | 7.0 |
| 1442710_at | Pdlim5 | PDZ and LIM domain 5 | 2.5 | 17.7 | 7.0 |
| 1418314_a_at | A2bp1 | ataxin 2 binding protein 1 | 1.8 | 12.4 | 6.9 |
| 1419220_at | Cmya1 | cardiomyopathy associated 1 | 1.4 | 9.2 | 6.8 |
| 1447658_x_at | Synpo2l | synaptopodin 2-like | 2.3 | 15.4 | 6.7 |
| 1452646_at | Trp53inp2 | tumor protein p53 inducible nuclear protein 2 | 3.5 | 23.3 | 6.7 |
| 1437598_at | Zbtb20 | Acyl-Coenzyme A binding domain containing 6, mRNA | 3.7 | 24.7 | 6.6 |
| 1422835_at | Kcnd2 | potassium voltage-gated channel, Shal-related family, member 2 | 1.3 | 8.6 | 6.6 |
| 1429223_a_at | Hfe2 | hemochromatosis type 2 (juvenile) (human homolog) | -1.2 | 5.5 | 6.5 |
| 1444139_at | Ddit4l | DNA-damage-inducible transcript 4-like | 1.6 | 10.7 | 6.5 |
| 1427177_at | Fyco1 | FYVE and coiled-coil domain containing 1 | 2.4 | 15.1 | 6.4 |
| 1436188_a_at | Ndrg4 | N-myc downstream regulated gene 4 | 1.7 | 10.9 | 6.3 |
| 1453421_at | Srr | serine racemase | 1.2 | 7.3 | 6.3 |
| 1418709_at | Cox7a1 | cytochrome c oxidase, subunit VIIa 1 | 1.5 | 9.4 | 6.3 |
| 1437751_at | Ppargc1a | peroxisome proliferative activated receptor, gamma, coactivator 1 alpha | 1.6 | 10.0 | 6.2 |
| 1457169_at | Ppp1r12b | protein phosphatase 1, regulatory (inhibitor) subunit 12B [Mus musculus] | -1.0 | 6.1 | 6.2 |
| 1455708_at | Tmod3 | Tropomodulin 3 (Tmod3), mRNA | 1.4 | 8.6 | 6.2 |
| 1444409_at | Kbtbd8 | Noc2 | 1.9 | 11.8 | 6.2 |
| 1429463_at | Prkaa2 | protein kinase, AMP-activated, alpha 2 catalytic subunit | 1.2 | 7.7 | 6.2 |
| 1455214_at | Mitf | microphthalmia-associated transcription factor | -2.2 | 2.7 | 6.1 |
| 1417416_at | Kcna1 | potassium voltage-gated channel, shaker-related subfamily, member 1 | 2.2 | 13.2 | 6.1 |
| 1438704_at | Trim63 | tripartite motif-containing 63 | 1.5 | 9.1 | 6.1 |
| 1449945_at | Ppargc1b | peroxisome proliferative activated receptor, gamma, coactivator 1 beta | 1.9 | 11.1 | 6.0 |
| 1434786_at | Ppp1r12b | protein phosphatase 1, regulatory (inhibitor) subunit 12B | 1.9 | 11.6 | 5.9 |
| 1456429_at | Malt1 | mucosa associated lymphoid tissue lymphoma translocation gene 1 | 1.4 | 7.9 | 5.7 |
| 1449501_a_at | Gzmm | granzyme M (lymphocyte met-ase 1) | -1.4 | 4.1 | 5.7 |
| 1431028_a_at | Pank1 | pantothenate kinase 1 | -1.5 | 3.7 | 5.7 |
| 1452399_at | Rgs6 | regulator of G-protein signaling 6 | 1.8 | 10.0 | 5.7 |
| 1432022_at | Cdgap | Cdc42 GTPase-activating protein | 2.3 | 13.3 | 5.7 |
| 1435285_at | Mpped2 | metallophosphoesterase domain containing 2 | 3.8 | 21.6 | 5.7 |
| 1438892_at | Qk | Quaking (Qk), mRNA | -1.1 | 5.0 | 5.6 |
| 1431751_a_at | Mpped2 | metallophosphoesterase domain containing 2 | 2.9 | 16.3 | 5.6 |
| 1448955_s_at | Cadps | Ca<2+>dependent activator protein for secretion | 1.6 | 8.6 | 5.4 |
| 1443983_at | Sorbs1 | Sorbin and SH3 domain containing 1, mRNA | 1.0 | 5.6 | 5.4 |
| 1426615_s_at | Ndrg4 | N-myc downstream regulated gene 4 | 1.8 | 9.9 | 5.4 |
| 1434960_at | Taf9b | TAF9-like RNA polymerase II, TATA box binding protein (TBP)-associated factor | 2.3 | 12.2 | 5.4 |
| 1417169_at | Usp2 | ubiquitin specific peptidase 2 | 1.3 | 7.0 | 5.3 |
| 1436378_at | Lrrfip2 | leucine rich repeat (in FLII) interacting protein 2 | -1.1 | 4.8 | 5.2 |
| 1456755_at | Trak1 | trafficking protein, kinesin binding 1 | 1.1 | 5.7 | 5.2 |
| 1441573_at | Scmh1 | Sex comb on midleg homolog 1 (Scmh1), mRNA | 1.7 | 8.7 | 5.2 |
| 1437092_at | Rsnl2 | restin-like 2 | 1.7 | 8.8 | 5.2 |
| 1421290_at | Hspb7 | heat shock protein family, member 7 (cardiovascular) | 1.3 | 6.5 | 5.2 |
| 1422834_at | Kcnd2 | potassium voltage-gated channel, Shal-related family, member 2 | -1.0 | 5.1 | 5.2 |
| 1458721_at | Pcdhgc3 | Protocadherin gamma subfamily A, 10 | 1.3 | 6.7 | 5.1 |
| 1435870_at | Sycp3 | Synaptonemal complex protein 3 | 2.7 | 13.7 | 5.1 |
| 1456960_at | Adk | Adenosine kinase | 1.0 | 5.1 | 5.1 |
| 1437224_at | Rtn4 | reticulon 4 | -1.1 | 4.4 | 5.0 |
| 1423420_at | Adrb1 | adrenergic receptor, beta 1 | 1.8 | 8.7 | 5.0 |
| 1455226_at | Spnb1 | similar to spectrin beta 1 | 1.4 | 6.7 | 4.9 |
| 1428557_a_at | Osgepl1 | O-sialoglycoprotein endopeptidase-like 1 | -1.2 | 4.0 | 4.9 |
| 1444477_at | Lrrtm3 | Leucine rich repeat transmembrane neuronal 3 | -1.2 | 4.3 | 4.9 |
| 1452879_at | Synpo2 | synaptopodin 2 | 2.3 | 11.1 | 4.9 |
| 1448183_a_at | Hif1a | hypoxia inducible factor 1, alpha subunit | -1.0 | 4.8 | 4.8 |
| 1441937_s_at | Pink1 | PTEN induced putative kinase 1 | 1.1 | 5.3 | 4.7 |
| 1459143_at | Chchd3 | coiled-coil-helix-coiled-coil-helix domain containing 3 | -1.0 | 4.6 | 4.7 |
| 1425292_at | Dtna | dystrobrevin alpha | -1.4 | 3.5 | 4.7 |
| 1438443_at | Zbtb20 | zinc finger and BTB domain containing 20 | 2.4 | 11.5 | 4.7 |
| 1441055_at | Palm2 | paralemmin 2 | 1.0 | 4.8 | 4.7 |
| 1449999_a_at | Cacna2d1 | calcium channel, voltage-dependent, alpha2/delta subunit 1 | 1.2 | 5.7 | 4.6 |
| 1447259_at | Ank3 | ankyrin 3, epithelial | -1.1 | 4.3 | 4.6 |
| 1455358_at | A2bp1 | Hexaribonucleotide binding protein 1 (Hrnbp1) | 1.2 | 5.5 | 4.6 |
| 1418744_s_at | Tesc | tescalcin | 2.1 | 9.7 | 4.6 |
| 1443475_at | Hist1h3i | Histone 1, H3i (Hist1h3i), mRNA | 1.0 | 4.7 | 4.6 |
| 1428547_at | Nt5e | 5' nucleotidase, ecto | 1.3 | 6.1 | 4.6 |
| 1438724_at | Osbpl3 | oxysterol binding protein-like 3 | 1.1 | 5.0 | 4.5 |
| 1418715_at | Pank1 | pantothenate kinase 1 | -1.6 | 2.8 | 4.5 |
| 1442029_at | Kcnq1 | KCNQ1b mRNA for Q1-type potassium channel spliced variant | -1.8 | 2.5 | 4.5 |
| 1445597_s_at | Hrasls3 | HRAS like suppressor 3 | -1.6 | 2.7 | 4.5 |
| 1460028_at | Grip2 | glutamate receptor interacting protein 2 | 1.0 | 4.5 | 4.4 |
| 1448381_at | Gfm1 | G elongation factor 1 | -2.1 | 2.1 | 4.4 |
| 1437765_at | Cpeb3 | cytoplasmic polyadenylation element binding protein 3 | -1.3 | 3.4 | 4.4 |
| 1447020_at | Zfpm2 | Zinc-finger factor FOG2 | 2.3 | 10.1 | 4.4 |
| 1429888_a_at | Hspb2 | heat shock protein 2 | 1.1 | 4.6 | 4.4 |
| 1439946_at | Mef2c | Myocyte enhancer factor 2C, mRNA | 1.3 | 5.7 | 4.3 |
| 1442166_at | Cpne5 | copine V | -1.9 | 2.3 | 4.3 |
| 1436119_at | Aldh1l2 | aldehyde dehydrogenase 1 family, member L2 | 1.4 | 6.2 | 4.3 |
| 1434314_s_at | Rab11fip5 | RAB11 family interacting protein 5 (class I) | 1.2 | 5.3 | 4.3 |
| 1427943_at | Acyp2 | acylphosphatase 2, muscle type | -1.8 | 2.4 | 4.3 |
| 1459961_a_at | Stat3 | signal transducer and activator of transcription 3 | 1.9 | 7.9 | 4.3 |
| 1425968_s_at | Speg | aortic preferentially expressed gene 1 | 1.5 | 6.4 | 4.3 |
| 1457126_at | Myl4 | myosin, light polypeptide 4 | 1.1 | 4.5 | 4.3 |
| 1440870_at | Prdm16 | MKIAA1675 protein | -1.2 | 3.6 | 4.1 |
| 1457356_at | Igf2r | Insulin-like growth factor 2 receptor (Igf2r), mRNA | 2.8 | 11.6 | 4.1 |
| 1416852_a_at | Ncdn | neurochondrin | -1.1 | 3.8 | 4.1 |
| 1433944_at | Hectd2 | HECT domain containing 2 | 2.2 | 8.8 | 4.1 |
| 1456542_s_at | Qrsl1 | glutaminyl-tRNA synthase (glutamine-hydrolyzing)-like 1 | -1.6 | 2.6 | 4.0 |
| 1427228_at | Palld | palladin, cytoskeletal associated protein | 1.9 | 7.7 | 4.0 |
| 1460434_at | Fundc2 | FUN14 domain containing 2 | 1.9 | 7.8 | 4.0 |
| 1448927_at | Kcnn2 | potassium intermediate/small conductance calcium-activated channel, subfamily N, member 2 | -1.5 | 2.6 | 4.0 |
| 1456735_x_at | Acpl2 | acid phosphatase-like 2 | 1.0 | 4.1 | 4.0 |
| 1435667_at | Rims1 | regulating synaptic membrane exocytosis 1 | -1.1 | 3.5 | 4.0 |
| 1459871_x_at | March2 | membrane-associated ring finger (C3HC4) 2 | 1.4 | 5.5 | 4.0 |
| 1438331_at | Ypel2 | Ypel2 mRNA for yippee-like 2 | 1.2 | 4.8 | 4.0 |
| 1422605_at | Ppp1r1a | protein phosphatase 1, regulatory (inhibitor) subunit 1A | -1.6 | 2.4 | 3.9 |
| 1435148_at | Atp1b2 | ATPase, Na+/K+ transporting, beta 2 polypeptide | 1.3 | 5.1 | 3.9 |
| 1429183_at | Pkp2 | plakophilin 2 | 2.0 | 7.8 | 3.9 |
| 1459670_at | Hnrpll | Heterogeneous nuclear ribonucleoprotein L-like (Hnrpll), mRNA | 1.3 | 5.0 | 3.9 |
| 1417384_at | Entpd5 | ectonucleoside triphosphate diphosphohydrolase 5 | 2.7 | 10.3 | 3.8 |
| 1456423_at | Mbd5 | methyl-CpG binding domain protein 5 | 1.7 | 6.4 | 3.8 |
| 1418062_at | Eef1a2 | eukaryotic translation elongation factor 1 alpha 2 | -1.1 | 3.6 | 3.8 |
| 1453821_at | Hemk2 | HemK methyltransferase family member 2 | 1.1 | 4.0 | 3.8 |
| 1436622_at | Iqsec2 | IQ motif and Sec7 domain 2 | 1.2 | 4.4 | 3.7 |
| 1428586_at | Tmem41b | transmembrane protein 41B | -1.0 | 3.6 | 3.7 |
| 1441364_at | Gata4 | Transcription factor GATA-4 | 1.8 | 6.7 | 3.7 |
| 1439168_at | Camk2d | calcium/calmodulin-dependent protein kinase II, delta | 1.6 | 6.1 | 3.7 |
| 1417008_at | Crat | carnitine acetyltransferase | 1.4 | 5.1 | 3.7 |
| 1448131_at | Mfn2 | mitofusin 2 | 2.2 | 8.1 | 3.7 |
| 1451628_a_at | Ank3 | ankyrin 3, epithelial | 1.2 | 4.5 | 3.7 |
| 1458455_at | Abra | actin-binding Rho activating protein | -1.1 | 3.3 | 3.7 |
| 1424698_s_at | Gca | grancalcin | 1.6 | 6.0 | 3.7 |
| 1434754_at | Garnl4 | GTPase activating RANGAP domain-like 4 | 1.8 | 6.5 | 3.7 |
| 1452005_at | Dlat | dihydrolipoamide S-acetyltransferase (E2 component of pyruvate dehydrogenase complex) | -1.3 | 2.9 | 3.7 |
| 1423856_at | Popdc3 | popeye domain containing 3 | 2.4 | 8.6 | 3.6 |
| 1422484_at | Cycs | cytochrome c, somatic | 1.5 | 5.3 | 3.6 |
| 1447174_at | Dach1 | DACH protein (Dach) | 1.5 | 5.2 | 3.6 |
| 1437724_x_at | Pitpnm1 | phosphatidylinositol membrane-associated 1 | 1.1 | 4.0 | 3.6 |
| 1420513_at | Efcab2 | EF-hand calcium binding domain 2 | -1.1 | 3.4 | 3.6 |
| 1457716_at | Za20d1 | zinc finger, A20 domain containing 1 | 1.4 | 5.1 | 3.6 |
| 1455903_at | Tbc1d4 | TBC1 domain family, member 4 | 1.7 | 6.2 | 3.6 |
| 1417286_at | Ndufa5 | NADH dehydrogenase (ubiquinone) 1 alpha subcomplex, 5 | 1.2 | 4.4 | 3.6 |
| 1439036_a_at | Atp1b1 | ATPase, Na+/K+ transporting, beta 1 polypeptide | 1.7 | 6.2 | 3.5 |
| 1442056_at | Zfp608 | Mus musculus zinc finger protein 608 (Zfp608), mRNA | 1.3 | 4.5 | 3.5 |
| 1440977_at | Akap13 | A kinase (PRKA) anchor protein 13 | 1.2 | 4.1 | 3.5 |
| 1452378_at | Malat1 | metastasis associated lung adenocarcinoma transcript 1 (non-coding RNA) | 1.2 | 4.3 | 3.5 |
| 1437067_at | Phtf2 | putative homeodomain transcription factor 2 | -1.4 | 2.6 | 3.5 |
| 1451431_a_at | Dbndd2 | DNA segment, Chr 2, Brigham & Women's Genetics 0891 expressed | 1.9 | 6.8 | 3.5 |
| 1453710_at | Tmem116 | transmembrane protein 116 | -1.1 | 3.0 | 3.4 |
| 1443158_at | Scmh1 | Sex comb on midleg homolog 1 (Scmh1), mRNA | 1.1 | 3.9 | 3.4 |
| 1440104_at | Ranbp2 | RAN binding protein 2 | 1.4 | 4.6 | 3.4 |
| 1457432_at | Prox1 | prospero-related homeobox 1 | 1.1 | 3.7 | 3.4 |
| 1443932_at | Klhdc1 | kelch domain containing 1 | 1.9 | 6.5 | 3.4 |
| 1453355_at | Wnk2 | WNK lysine deficient protein kinase 2 | 1.4 | 4.5 | 3.3 |
| 1417273_at | Pdk4 | pyruvate dehydrogenase kinase, isoenzyme 4 | 1.2 | 3.9 | 3.3 |
| 1456487_at | Adcy1 | adenylate cyclase 1 | 1.4 | 4.5 | 3.3 |
| 1418288_at | Lpin1 | lipin 1 | 1.5 | 5.1 | 3.3 |
| 1423653_at | Atp1a1 | ATPase, Na+/K+ transporting, alpha 1 polypeptide | 1.4 | 4.5 | 3.3 |
| 1452265_at | Clasp1 | CLIP associating protein 1 | 2.1 | 7.0 | 3.3 |
| 1431035_at | Daam1 | dishevelled associated activator of morphogenesis 1 | 1.8 | 5.8 | 3.2 |
| 1455614_at | Tomm40l | nuclear receptor subfamily 1, group I, member 3 | 1.2 | 3.9 | 3.2 |
| 1418258_s_at | Dynll2 | dynein light chain LC8-type 2 | 1.8 | 5.7 | 3.2 |
| 1421087_at | Per3 | period homolog 3 (Drosophila) | -1.1 | 2.8 | 3.2 |
| 1426272_at | Lmbr1 | limb region 1 | -1.1 | 3.0 | 3.2 |
| 1426856_at | Hsdl2 | hydroxysteroid dehydrogenase like 2 | 1.8 | 5.7 | 3.2 |
| 1417970_at | Atp5s | ATP synthase, H+ transporting, mitochondrial F0 complex, subunit s | -1.4 | 2.3 | 3.2 |
| 1436468_at | Zdhhc8 | zinc finger, DHHC domain containing 8 | -1.2 | 2.7 | 3.2 |
| 1428749_at | Dmxl2 | Dmx-like 2 | -1.3 | 2.4 | 3.2 |
| 1457997_at | Leng4 | leukocyte receptor cluster (LRC) member 4 | -1.1 | 2.9 | 3.2 |
| 1436934_s_at | Aco2 | aconitase 2, mitochondrial | 1.1 | 3.4 | 3.2 |
| 1438613_at | Kcna4 | potassium voltage-gated channel, shaker-related subfamily, member 4 | 1.1 | 3.3 | 3.2 |
| 1449799_s_at | Pkp2 | plakophilin 2 | 2.1 | 6.5 | 3.2 |
| 1450101_a_at | Magi3 | membrane associated guanylate kinase, WW and PDZ domain containing 3 | 1.3 | 4.2 | 3.1 |
| 1457297_at | Lysmd4 | MEF2A mRNA, partial 3' UTR sequence | 1.1 | 3.5 | 3.1 |
| 1438691_at | Zzef1 | zinc finger, ZZ-type with EF hand domain 1 | -1.2 | 2.7 | 3.1 |
| 1457338_at | Ppp1r12b | protein phosphatase 1, regulatory (inhibitor) subunit 12B | -1.2 | 2.7 | 3.1 |
| 1435807_at | Cdc42 | cell division cycle 42 homolog (S. cerevisiae) | 2.1 | 6.4 | 3.1 |
| 1417285_a_at | Ndufa5 | NADH dehydrogenase (ubiquinone) 1 alpha subcomplex, 5 | 1.5 | 4.5 | 3.1 |
| 1444874_at | Atp5g1 | ATP synthase, H+ transporting, mitochondrial F0 complex, subunit c (subunit 9), isoform 1 | 1.0 | 3.2 | 3.1 |
| 1434657_at | Gls | Glutaminase | 1.8 | 5.6 | 3.1 |
| 1440880_at | Mppe1 | metallophosphoesterase 1 | 1.2 | 3.6 | 3.1 |
| 1444598_at | Etv6 | ets variant gene 6 (TEL oncogene) | 1.1 | 3.4 | 3.1 |
| 1434053_x_at | Atp5k | ATP synthase, H+ transporting, mitochondrial F1F0 complex, subunit e | 1.3 | 3.9 | 3.0 |
| 1458263_at | Cugbp2 | CUG triplet repeat, RNA binding protein 2 | 1.0 | 3.1 | 3.0 |
| 1460580_at | Pcnx | pecanex homolog (Drosophila) | 1.1 | 3.3 | 3.0 |
| 1423833_a_at | Brp44 | brain protein 44 | 2.2 | 6.5 | 3.0 |
| 1435153_at | Btbd6 | BTB (POZ) domain containing 6 | 1.2 | 3.5 | 3.0 |
| 1451071_a_at | Atp1a1 | ATPase, Na+/K+ transporting, alpha 1 polypeptide | 1.4 | 4.2 | 3.0 |
| 1443037_at | Nptn | Stromal cell derived factor receptor 1 (Sdfr1), mRNA | -1.4 | 2.2 | 3.0 |
| 1448724_at | Cish | cytokine inducible SH2-containing protein | 1.9 | 5.6 | 3.0 |
| 1441730_at | Ppp1r12b | protein phosphatase 1, regulatory (inhibitor) subunit 12B | -1.1 | 2.7 | 3.0 |
| 1416996_at | Tbc1d8 | TBC1 domain family, member 8 | -1.1 | 2.6 | 3.0 |
| 1459754_x_at | Eef1d | eukaryotic translation elongation factor 1 delta (guanine nucleotide exchange protein) | -1.1 | 2.6 | 3.0 |
| 1438501_at | Rps17 | Adult male cecum cDNA, RIKEN full-length enriched library, clone:9130403J09 product:ribosomal protein S17, full insert sequence | -1.0 | 2.9 | 2.9 |
| 1460694_s_at | Svil | supervillin | 1.4 | 4.0 | 2.9 |
| 1450162_at | Dpf3 | D4, zinc and double PHD fingers, family 3 | 1.9 | 5.6 | 2.9 |
| 1437164_x_at | Atp5o | ATP synthase, H+ transporting, mitochondrial F1 complex, O subunit; similar to ATP synthase, H+ transporting, mitochondrial F1 complex, O subunit | -1.0 | 2.9 | 2.9 |
| 1436436_at | Cnih4 | cornichon homolog 4 (Drosophila) | 1.3 | 3.8 | 2.9 |
| 1451148_at | Pink1 | PTEN induced putative kinase 1 | 1.2 | 3.6 | 2.9 |
| 1455703_at | Akt2 | thymoma viral proto-oncogene 2 | -1.3 | 2.3 | 2.9 |
| 1435021_at | Gabrb3 | gamma-aminobutyric acid (GABA-A) receptor, subunit beta 3 | -1.3 | 2.3 | 2.9 |
| 1425673_at | Lpp | LIM domain containing preferred translocation partner in lipoma | 1.6 | 4.7 | 2.9 |
| 1435746_at | Srpk2 | serine/arginine-rich protein specific kinase 2 | -1.2 | 2.4 | 2.9 |
| 1442216_at | Atp6v0d1 | ATPase, H+ transporting, V0 subunit D isoform 1 | 1.0 | 2.9 | 2.9 |
| 1441435_at | Tbl1x | transducin (beta)-like 1 X-linked | -1.4 | 2.0 | 2.9 |
| 1439787_at | P2rx7 | purinergic receptor P2X, ligand-gated ion channel, 7 | -1.4 | 2.0 | 2.9 |
| 1456573_x_at | Nnt | nicotinamide nucleotide transhydrogenase | -1.0 | 2.8 | 2.9 |
| 1458385_at | Hspa4l | heat shock 70kDa protein 4 like | 1.7 | 4.7 | 2.9 |
| 1424415_s_at | Spon1 | spondin 1, (f-spondin) extracellular matrix protein | 1.2 | 3.3 | 2.8 |
| 1449623_at | Txnrd3 | thioredoxin reductase 3 | -1.4 | 2.0 | 2.8 |
| 1444431_at | Rcsd1 | RCSD domain containing 1 | 1.1 | 3.0 | 2.8 |
| 1448292_at | Uqcr | ubiquinol-cytochrome c reductase (6.4kD) subunit | 1.2 | 3.5 | 2.8 |
| 1441581_at | Asb10 | ankyrin repeat and SOCS box-containing protein 10 | -1.1 | 2.7 | 2.8 |
| 1421340_at | Map3k5 | mitogen activated protein kinase kinase kinase 5 | 1.0 | 2.9 | 2.8 |
| 1427955_a_at | Deb1 | differentially expressed in B16F10 1 | 1.6 | 4.4 | 2.8 |
| 1418321_at | Dci | dodecenoyl-Coenzyme A delta isomerase (3,2 trans-enoyl-Coenyme A isomerase) | -1.0 | 2.8 | 2.8 |
| 1433970_at | Bola3 | bolA-like 3 (E. coli) | 1.7 | 4.8 | 2.8 |
| 1436803_a_at | Ndufb9 | NADH dehydrogenase (ubiquinone) 1 beta subcomplex, 9 | -1.2 | 2.4 | 2.8 |
| 1455244_at | Daam1 | dishevelled associated activator of morphogenesis 1 | 1.8 | 5.0 | 2.8 |
| 1423707_at | Tmem50b | transmembrane protein 50B | 1.5 | 4.3 | 2.8 |
| 1418586_at | Adcy9 | adenylate cyclase 9 | -1.0 | 2.7 | 2.8 |
| 1444328_at | Clta | clathrin, light polypeptide (Lca) | -1.3 | 2.1 | 2.8 |
| 1452864_at | Med12l | mediator of RNA polymerase II transcription, subunit 12 homolog (yeast)-like | 1.5 | 4.1 | 2.8 |
| 1450667_a_at | Cs | citrate synthase | 1.2 | 3.5 | 2.8 |
| 1442102_at | Aco2 | Polymerase (RNA) III (DNA directed) polypeptide H (Polr3h), mRNA | -1.3 | 2.2 | 2.8 |
| 1420259_at | Pkp2 | Plakophilin 2 (Pkp2), mRNA | 1.1 | 3.0 | 2.8 |
| 1427984_at | Senp6 | SUMO/sentrin specific peptidase 6 | 1.1 | 3.0 | 2.8 |
| 1441869_x_at | Auts2 | PREDICTED: hypothetical protein XP_488533 [Mus musculus], mRNA sequence | 1.4 | 3.9 | 2.8 |
| 1441560_at | Ctnna3 | catenin (cadherin associated protein), alpha 3 | -1.0 | 2.7 | 2.7 |
| 1451481_s_at | Osta | organic solute transporter alpha | -1.1 | 2.6 | 2.7 |
| 1423365_at | Cacna1g | calcium channel, voltage-dependent, T type, alpha 1G subunit | 1.8 | 4.9 | 2.7 |
| 1427104_at | Zfp612 | zinc finger protein 612 | 1.2 | 3.2 | 2.7 |
| 1446258_at | Sntb1 | syntrophin, basic 1 | -1.1 | 2.5 | 2.7 |
| 1428578_s_at | Ppfia4 | protein tyrosine phosphatase, receptor type, f polypeptide (PTPRF), interacting protein (liprin), alpha 4 | 1.0 | 2.8 | 2.7 |
| 1455594_at | Exoc3 | exocyst complex component 3 | 1.5 | 4.1 | 2.7 |
| 1460285_at | Itga9 | integrin alpha 9 | 1.2 | 3.4 | 2.7 |
| 1456480_at | Fry | RIKEN cDNA 9330186A19 gene | -1.2 | 2.2 | 2.7 |
| 1457311_at | Camk2a | calcium/calmodulin-dependent protein kinase II alpha | 1.2 | 3.2 | 2.7 |
| 1434611_at | Rnf123 | ring finger protein 123 | -1.2 | 2.2 | 2.7 |
| 1453636_at | Pcgf5 | polycomb group ring finger 5 | 1.1 | 2.9 | 2.7 |
| 1448934_at | Ndufa10 | NADH dehydrogenase (ubiquinone) 1 alpha subcomplex 10 | 1.0 | 2.7 | 2.7 |
| 1435055_a_at | Tom1 | target of myb1 homolog (chicken); similar to TOM1 | -1.3 | 2.1 | 2.6 |
| 1447701_x_at | Idh3a | isocitrate dehydrogenase 3 (NAD+) alpha | -1.3 | 2.0 | 2.6 |
| 1446141_at | Tgfb2 | Transforming growth factor, beta 2 | 1.1 | 2.9 | 2.6 |
| 1457246_at | Rai16 | retinoic acid induced 16 | 1.4 | 3.6 | 2.6 |
| 1420833_at | Vamp2 | vesicle-associated membrane protein 2 | 1.4 | 3.8 | 2.6 |
| 1456812_at | Abcd2 | ATP-binding cassette, sub-family D (ALD), member 2 | 1.2 | 3.2 | 2.6 |
| 1441056_at | Usp3 | Ubiquitin specific peptidase 3 (Usp3), mRNA | 1.3 | 3.4 | 2.6 |
| 1429914_at | Epc1 | enhancer of polycomb homolog 1 (Drosophila) | 1.1 | 2.8 | 2.6 |
| 1419835_s_at | Plec1 | plectin 1 | -1.1 | 2.5 | 2.6 |
| 1443511_at | Rora | Transcribed locus | -1.2 | 2.2 | 2.6 |
| 1429139_at | Za20d1 | zinc finger, A20 domain containing 1 | 1.5 | 3.8 | 2.6 |
| 1423907_a_at | Ndufs8 | NADH dehydrogenase (ubiquinone) Fe-S protein 8 | 1.2 | 3.0 | 2.6 |
| 1441259_s_at | Ift122 | WD repeat domain 10 | 1.0 | 2.7 | 2.6 |
| 1459377_at | Palm2 | paralemmin 2 | -1.0 | 2.6 | 2.6 |
| 1423108_at | Slc25a20 | solute carrier family 25 (mitochondrial carnitine/acylcarnitine translocase), member 20 | 1.2 | 3.2 | 2.6 |
| 1439857_at | Usp32 | Ubiquitin specific peptidase 32 | -1.1 | 2.3 | 2.6 |
| 1417316_at | Them2 | thioesterase superfamily member 2 | 1.3 | 3.3 | 2.6 |
| 1436695_x_at | Rbed1 | RNA binding motif and ELMO domain 1 | -1.0 | 2.5 | 2.6 |
| 1441953_at | Stox2 | Storkhead box 2 | 1.1 | 2.8 | 2.6 |
| 1421152_a_at | Gnao1 | guanine nucleotide binding protein, alpha o | 1.1 | 2.9 | 2.6 |
| 1455079_at | Dcun1d4 | DCN1, defective in cullin neddylation 1, domain containing 4 (S. cerevisiae) | -1.2 | 2.2 | 2.6 |
| 1424313_a_at | Ndufs7 | NADH dehydrogenase (ubiquinone) Fe-S protein 7 | 1.1 | 2.9 | 2.6 |
| 1421282_at | Bmp5 | bone morphogenetic protein 5 | 1.6 | 4.0 | 2.6 |
| 1444956_at | Gabarapl2 | gamma-aminobutyric acid (GABA-A) receptor-associated protein-like 2 | 1.6 | 4.0 | 2.6 |
| 1455806_x_at | Ndufa12 | NADH dehydrogenase (ubiquinone) 1 alpha subcomplex, 12 | 1.2 | 3.1 | 2.5 |
| 1446783_at | Gadd45gip1 | RAD23a homolog (S. cerevisiae) (Rad23a), mRNA | -1.2 | 2.1 | 2.5 |
| 1455936_a_at | Rbpms | RNA binding protein gene with multiple splicing | 1.1 | 2.9 | 2.5 |
| 1422642_at | Cdc42ep3 | CDC42 effector protein (Rho GTPase binding) 3 | 1.6 | 4.2 | 2.5 |
| 1436737_a_at | Sorbs1 | sorbin and SH3 domain containing 1 | 1.6 | 4.1 | 2.5 |
| 1459457_at | Camk2d | calcium/calmodulin-dependent protein kinase II, delta | 1.4 | 3.5 | 2.5 |
| 1457177_at | Rora | RAR-related orphan receptor alpha | 1.0 | 2.5 | 2.5 |
| 1434672_at | Gpr22 | G protein-coupled receptor 22 | 1.0 | 2.6 | 2.5 |
| 1453409_at | Cgrrf1 | cell growth regulator with ring finger domain 1 | 1.3 | 3.3 | 2.5 |
| 1455967_at | Sorbs1 | sorbin and SH3 domain containing 1 | -1.1 | 2.3 | 2.5 |
| 1428206_at | Ccdc69 | DNA segment, Chr 11, ERATO Doi 461, expressed | -1.2 | 2.1 | 2.5 |
| 1422025_at | Mitf | microphthalmia-associated transcription factor | -1.2 | 2.0 | 2.5 |
| 1440565_at | Zbtb20 | zinc finger and BTB domain containing 20 | -1.1 | 2.3 | 2.4 |
| 1428322_a_at | Ndufb10 | NADH dehydrogenase (ubiquinone) 1 beta subcomplex, 10 | -1.0 | 2.3 | 2.4 |
| 1419974_at | Scp2 | sterol carrier protein 2, liver | 1.2 | 2.9 | 2.4 |
| 1456071_a_at | Cycs | cytochrome c, somatic | 1.6 | 3.9 | 2.4 |
| 1452213_at | Tex2 | testis expressed gene 2 | -1.1 | 2.1 | 2.4 |
| 1422241_a_at | Ndufa1 | NADH dehydrogenase (ubiquinone) 1 alpha subcomplex, 1 | -1.1 | 2.3 | 2.4 |
| 1448198_a_at | Ndufb8 | NADH dehydrogenase (ubiquinone) 1 beta subcomplex 8 | 1.1 | 2.5 | 2.4 |
| 1456072_at | Ppp1r9a | protein phosphatase 1, regulatory (inhibitor) subunit 9A | 1.5 | 3.5 | 2.4 |
| 1446632_at | Cacnb2 | Calcium channel, voltage-dependent, beta 2 subunit (Cacnb2), mRNA | 1.2 | 2.8 | 2.4 |
| 1441230_at | Fndc3b | MKIAA4164 protein | -1.1 | 2.1 | 2.4 |
| 1428783_at | Prkar2a | protein kinase, cAMP dependent regulatory, type II alpha | 1.5 | 3.5 | 2.4 |
| 1435520_at | Msi2 | Musashi homolog 2 (Drosophila) | 1.0 | 2.4 | 2.4 |
| 1449686_s_at | Scp2 | sterol carrier protein 2, liver | 1.6 | 3.9 | 2.4 |
| 1433773_at | Rrm2b | ribonucleotide reductase M2 B (TP53 inducible) | 1.4 | 3.4 | 2.4 |
| 1456871_a_at | Phf20l1 | PHD finger protein 20-like 1 | 1.3 | 3.1 | 2.4 |
| 1416910_at | Dnajc15 | DnaJ (Hsp40) homolog, subfamily C, member 15 | 1.1 | 2.6 | 2.3 |
| 1441445_at | Per3 | period homolog 3 (Drosophila) | -1.1 | 2.2 | 2.3 |
| 1427285_s_at | Malat1 | metastasis associated lung adenocarcinoma transcript 1 (non-coding RNA) | 1.2 | 2.8 | 2.3 |
| 1448853_at | Synj2bp | synaptojanin 2 binding protein | -1.1 | 2.2 | 2.3 |
| 1423692_at | Ndufa8 | NADH dehydrogenase (ubiquinone) 1 alpha subcomplex, 8 | 1.4 | 3.2 | 2.3 |
| 1452944_at | Afmid | synaptogyrin 2 | -1.0 | 2.3 | 2.3 |
| 1417382_at | Entpd5 | ectonucleoside triphosphate diphosphohydrolase 5 | 1.7 | 3.9 | 2.3 |
| 1444698_at | Qk | Quaking (Qk), mRNA | -1.1 | 2.1 | 2.3 |
| 1430856_at | Pex11c | peroxisomal biogenesis factor 11c | -1.1 | 2.2 | 2.3 |
| 1438166_x_at | Ndufs4 | NADH dehydrogenase (ubiquinone) Fe-S protein 4 | -1.1 | 2.2 | 2.3 |
| 1455283_x_at | Ndufs8 | NADH dehydrogenase (ubiquinone) Fe-S protein 8 | 1.2 | 2.7 | 2.3 |
| 1458155_at | Rabgap1l | RAB GTPase activating protein 1-like | -1.0 | 2.3 | 2.3 |
| 1434579_x_at | Ndufs8 | NADH dehydrogenase (ubiquinone) Fe-S protein 8 | 1.2 | 2.7 | 2.3 |
| 1417264_at | Coq5 | DNA segment, Chr 5, ERATO Doi 33, expressed | -1.1 | 2.1 | 2.3 |
| 1428724_at | Pcf11 | cleavage and polyadenylation factor subunit homolog (S. cerevisiae) | 1.3 | 3.0 | 2.3 |
| 1429214_at | Adamtsl2 | ADAMTS-like 2 | 1.1 | 2.6 | 2.3 |
| 1422665_a_at | Pcmt1 | protein-L-isoaspartate (D-aspartate) O-methyltransferase 1 | 1.1 | 2.5 | 2.3 |
| 1435543_at | Apc | Adenomatosis polyposis coli (Apc), mRNA | 1.2 | 2.7 | 2.3 |
| 1417191_at | Dnajb9 | DnaJ (Hsp40) homolog, subfamily B, member 9 | 1.6 | 3.5 | 2.3 |
| 1436510_a_at | Lrrfip2 | leucine rich repeat (in FLII) interacting protein 2 | 1.1 | 2.4 | 2.3 |
| 1434355_at | Zfp617 | Zinc finger protein 617 (Zfp617), mRNA | 1.2 | 2.7 | 2.3 |
| 1437744_at | Slitrk4 | SLIT and NTRK-like family, member 4 | 1.5 | 3.3 | 2.3 |
| 1452357_at | Gp1bb | glycoprotein Ib, beta polypeptide; septin 5 | 1.7 | 3.9 | 2.3 |
| 1436833_x_at | Ttll1 | tubulin tyrosine ligase-like 1 | 1.2 | 2.6 | 2.3 |
| 1433513_x_at | Ndufa12 | NADH dehydrogenase (ubiquinone) 1 alpha subcomplex, 12 | 1.2 | 2.7 | 2.2 |
| 1460735_at | Svil | supervillin | 1.2 | 2.6 | 2.2 |
| 1452166_a_at | Krt1-10 | keratin complex 1, acidic, gene 10 | 1.4 | 3.1 | 2.2 |
| 1436166_at | Stox2 | storkhead box 2 | 1.5 | 3.4 | 2.2 |
| 1425677_a_at | Ank1 | ankyrin 1, erythroid | -1.0 | 2.1 | 2.2 |
| 1429064_at | Dip2c | DIP2 disco-interacting protein 2 homolog C (Drosophila) | 1.2 | 2.8 | 2.2 |
| 1434213_x_at | Ndufs8 | NADH dehydrogenase (ubiquinone) Fe-S protein 8 | 1.1 | 2.5 | 2.2 |
| 1449997_at | Tpm3 | tropomyosin 3, gamma | 1.2 | 2.6 | 2.2 |
| 1416417_a_at | Ndufb7 | NADH dehydrogenase (ubiquinone) 1 beta subcomplex, 7 | 1.4 | 3.1 | 2.2 |
| 1449884_at | Efcab2 | EF-hand calcium binding domain 2 | 1.0 | 2.2 | 2.2 |
| 1445723_at | Plcl1 | phospholipase C-like 1 | 1.6 | 3.6 | 2.2 |
| 1449421_a_at | Kcne2 | potassium voltage-gated channel, Isk-related subfamily, gene 2 | -1.0 | 2.2 | 2.2 |
| 1449181_at | Fech | ferrochelatase | 1.6 | 3.5 | 2.2 |
| 1458381_at | Clic5 | chloride intracellular channel 5 | 1.2 | 2.8 | 2.2 |
| 1417383_at | Entpd5 | ectonucleoside triphosphate diphosphohydrolase 5 | 1.0 | 2.3 | 2.2 |
| 1458685_at | Garnl1 | GTPase activating RANGAP domain-like 1 | 1.0 | 2.2 | 2.2 |
| 1419256_at | Spnb2 | spectrin beta 2 | 1.7 | 3.7 | 2.2 |
| 1423738_at | Oxa1l | oxidase assembly 1-like | -1.1 | 2.1 | 2.2 |
| 1441507_at | Spnb2 | spectrin beta 2 | 1.0 | 2.3 | 2.2 |
| 1445984_at | Pftk1 | PFTAIRE protein kinase 1 (Pftk1), mRNA | 1.5 | 3.3 | 2.2 |
| 1437619_x_at | Ddr1 | discoidin domain receptor family, member 1 | -1.1 | 2.0 | 2.2 |
| 1418074_at | St6galnac4 | ST6 (alpha-N-acetyl-neuraminyl-2,3-beta-galactosyl-1,3)-N-acetylgalactosaminide alpha-2,6-sialyltransferase 4 | 1.1 | 2.3 | 2.2 |
| 1417405_at | Stard3 | START domain containing 3 | 1.6 | 3.5 | 2.2 |
| 1442021_at | Gnal | guanine nucleotide binding protein, alpha stimulating, olfactory type | 1.1 | 2.4 | 2.2 |
| 1458700_at | Lrrc8c | Leucine rich repeat containing 8 family, member C | 1.1 | 2.5 | 2.2 |
| 1457944_at | Etv6 | Ets variant gene 6 (TEL oncogene) (Etv6), mRNA | 1.2 | 2.6 | 2.2 |
| 1438435_at | Phca | phytoceramidase, alkaline | 1.1 | 2.4 | 2.2 |
| 1424319_at | Oraov1 | oral cancer overexpressed 1 | -1.0 | 2.2 | 2.2 |
| 1446835_at | Ftl1 | ferritin light chain 1 | 1.7 | 3.7 | 2.2 |
| 1435757_a_at | Uqcrc2 | ubiquinol cytochrome c reductase core protein 2 | 1.1 | 2.4 | 2.2 |
| 1456126_at | Malt1 | mucosa associated lymphoid tissue lymphoma translocation gene 1 | 1.2 | 2.6 | 2.1 |
| 1433566_at | Rasl10b | RAS-like, family 10, member B | -1.0 | 2.1 | 2.1 |
| 1460074_x_at | Epdr2 | ependymin related protein 2 (zebrafish) | -1.0 | 2.1 | 2.1 |
| 1430979_a_at | Prdx2 | peroxiredoxin 2 | 1.2 | 2.5 | 2.1 |
| 1416494_at | Ndufs5 | NADH dehydrogenase (ubiquinone) Fe-S protein 5 | -1.0 | 2.1 | 2.1 |
| 1423632_at | Gpr146 | G protein-coupled receptor 146 | 1.5 | 3.3 | 2.1 |
| 1421289_at | Hspb7 | heat shock protein family, member 7 (cardiovascular) | 1.0 | 2.1 | 2.1 |
| 1417102_a_at | Ndufb5 | NADH dehydrogenase (ubiquinone) 1 beta subcomplex, 5 | 1.0 | 2.1 | 2.1 |
| 1453898_at | Itgb1bp3 | integrin beta 1 binding protein 3 | 1.1 | 2.2 | 2.1 |
| 1444100_at | Pdpn | podoplanin | 1.3 | 2.7 | 2.1 |
| 1422998_a_at | Glrx2 | glutaredoxin 2 (thioltransferase) | 1.1 | 2.3 | 2.1 |
| 1442279_at | Epc1 | Enhancer of polycomb homolog 1 (Drosophila) (Epc1), transcript variant 1, mRNA | 1.1 | 2.2 | 2.1 |
| 1422756_at | Slc32a1 | solute carrier family 32 (GABA vesicular transporter), member 1 | 1.2 | 2.5 | 2.1 |
| 1444396_at | Trp53inp2 | Tumor protein p53 inducible nuclear protein 2, mRNA | 1.2 | 2.5 | 2.1 |
| 1460630_at | Camk2d | calcium/calmodulin-dependent protein kinase II, delta | 1.3 | 2.6 | 2.1 |
| 1417799_at | Atp6v1g2 | ATPase, H+ transporting, V1 subunit G isoform 2 | 1.1 | 2.3 | 2.1 |
| 1447231_at | Slc8a1 | Solute carrier family 8 (sodium/calcium exchanger), member 1 (Slc8a1), mRNA | 1.0 | 2.1 | 2.1 |
| 1447919_x_at | Ndufab1 | NADH dehydrogenase (ubiquinone) 1, alpha/beta subcomplex, 1 | 1.1 | 2.3 | 2.1 |
| 1455027_at | Rufy3 | DNA segment, Chr 5, Brigham & Women's Genetics 0860 expressed | 1.1 | 2.2 | 2.1 |
| 1428513_at | Calcoco1 | calcium binding and coiled coil domain 1 | 1.0 | 2.1 | 2.1 |
| 1426778_at | Dag1 | dystroglycan 1 | 1.4 | 2.9 | 2.1 |
| 1451736_a_at | Map2k7 | mitogen activated protein kinase kinase 7 | -1.0 | 2.0 | 2.1 |
| 1427145_at | Iqsec1 | IQ motif and Sec7 domain 1 | 1.0 | 2.1 | 2.1 |
| 1425983_x_at | Hipk2 | homeodomain interacting protein kinase 2 | 1.1 | 2.2 | 2.0 |
| 1423341_at | Cspg4 | chondroitin sulfate proteoglycan 4 | 1.4 | 2.9 | 2.0 |
| 1450640_x_at | Atp5k | ATP synthase, H+ transporting, mitochondrial F1F0 complex, subunit e | 1.1 | 2.3 | 2.0 |
| 1448284_a_at | Ndufc1 | NADH dehydrogenase (ubiquinone) 1, subcomplex unknown, 1 | 1.2 | 2.4 | 2.0 |
| 1424422_s_at | Lenep | lens epithelial protein; RFad1, flavin adenine dinucleotide synthetase, homolog (yeast) | 1.1 | 2.2 | 2.0 |
| 1423242_at | Mrps36 | mitochondrial ribosomal protein S36 | 1.3 | 2.7 | 2.0 |
| 1422627_a_at | Mkks | McKusick-Kaufman syndrome protein | 1.6 | 3.3 | 2.0 |
| 1424979_at | Aph1a | anterior pharynx defective 1a homolog (C. elegans) | 1.1 | 2.3 | 2.0 |
| 1419805_s_at | Ggps1 | geranylgeranyl diphosphate synthase 1 | 1.3 | 2.6 | 2.0 |
| 1449843_at | St8sia2 | ST8 alpha-N-acetyl-neuraminide alpha-2,8-sialyltransferase 2 | 1.1 | 2.3 | 2.0 |
| 1436405_at | Dock4 | dedicator of cytokinesis 4 | 1.2 | 2.5 | 2.0 |
| 1438848_at | Rbpsuh | Oxysterol binding protein | 1.1 | 2.2 | 2.0 |
| 1437172_x_at | Hadhb | hydroxyacyl-Coenzyme A dehydrogenase/3-ketoacyl-Coenzyme A thiolase/enoyl-Coenzyme A hydratase (trifunctional protein), beta subunit | 1.4 | 2.8 | 2.0 |

Lists of probe sets for the subclusters A and B as identified in the hierarchical clustering of probe sets upregulated in α-MHC^+^ cells (Fig. 5). Probe sets are listed with the corresponding gene symbol and gene title. Fold changes (fc) are given for pairwise comparisons between undifferentiated α-MHC ES cells (d0) and day 15 control EBs (d15), between undifferentiated α-MHC ES cells (d0) and 15 day old α-MHC^+^ cardiomyocytes (α-MHC^+^) as well as between day 15 control EBs (d15) and 15 day old α-MHC^+^ cardiomyocytes (α-MHC^+^).
