# Supplementary material for: Global transcriptome analysis of murine embryonic stem cell-derived cardiomyocytes
Source: Genome Biol. 2007 Apr 11;8(4):R56. doi: 10.1186/gb-2007-8-4-r56 (PMC1896009; doi:10.1186/gb-2007-8-4-r56)
Supplement: Additional data file 5 — Part a provides the genes belonging to the GOTERM_CC categories 'myofibril, striated muscle thin filament, actin cytoskeleton', 'cytoskeleton' and 'myosin', and GOTERM_BP category 'cytoskeleton organization and biogenesis' that are upregulated in the α-MHC+ cardiomyocytes (intersection of upregulation in α-MHC+ cardiomyocytes [twofold, t-test P value < 0.01] compared with control cells in the 15-day-old EBs and compared with undifferentiated α-MHC ES cells). Part b provides the genes belonging to the GOTERM_MF categories 'voltage-gated ion channel activity' that are upregulated in the α-MHC+ cardiomyocytes (intersection of upregulation in α-MHC+ cardiomyocytes [twofold, t-test P value < 0.01] compared with control cells in the 15-day-old EBs and compared with undifferentiated α-MHC ES cells). [file gb-2007-8-4-r56-S5.doc]

**Additional Data file 5a:**

| Probe sets | Symbol | Title | **fc d0**  **vs. d15** | **fc d0 vs. αMHC^+^** | **fc d15 vs. αMHC^+^** |
| --- | --- | --- | --- | --- | --- |
| 1417729_at | Myh6 | myosin, heavy polypeptide 6, cardiac muscle, alpha | 2.1 | 81.4 | 37.9 |
| 1450917_at | Myom2 | myomesin 2 | 1.3 | 41.9 | 31.4 |
| 1442715_at | Dmd | Dystrophin, muscular dystrophy | -1.0 | 24.2 | 24.7 |
| 1444638_at | Ttn | titin | 6.3 | 137.3 | 21.8 |
| 1448827_s_at | Myh6; Myh7 | myosin, heavy polypeptide 6, cardiac muscle, alpha; myosin, heavy polypeptide 7, cardiac muscle, beta | 4.2 | 88.1 | 21.0 |
| 1452345_at | Lmod2 | leiomodin 2 (cardiac) | 2.2 | 34.1 | 15.8 |
| 1419440_at | Trim54 | tripartite motif-containing 54 | -1.2 | 12.2 | 14.4 |
| 1418769_at | Myoz2 | myozenin 2 | 14.2 | 181.5 | 12.8 |
| 1448327_at | Actn2 | actinin alpha 2 | 16.4 | 204.1 | 12.4 |
| 1451830_a_at | Spnb2 | spectrin beta 2 | 2.3 | 25.9 | 11.4 |
| 1422754_at | Tmod1 | tropomodulin 1 | 5.8 | 57.1 | 9.8 |
| 1447713_at | Tpm1 | tropomyosin 1, alpha | 6.2 | 58.3 | 9.4 |
| 1447853_x_at | Kif13a | KIF13A | -1.2 | 7.0 | 8.7 |
| 1422536_at | Tnni3 | troponin I, cardiac | 12.9 | 110.4 | 8.6 |
| 1447657_s_at | Synpo2l | synaptopodin 2-like | 6.2 | 50.9 | 8.2 |
| 1429783_at | Pdlim5 | PDZ and LIM domain 5 | 8.1 | 64.9 | 8.0 |
| 1428266_at | Myl3 | myosin, light polypeptide 3 | 20.0 | 152.6 | 7.6 |
| 1419606_a_at | Tnnt1 | troponin T1, skeletal, slow | 1.2 | 9.0 | 7.5 |
| 1418589_a_at | Mlf1 | myeloid leukemia factor 1 | 8.7 | 61.4 | 7.1 |
| 1455493_at | Syne1 | synaptic nuclear envelope 1 | 2.3 | 16.4 | 7.1 |
| 1440424_at | Tnnt2 | Troponin T2, cardiac | 1.7 | 12.1 | 7.0 |
| 1455708_at | Tmod3 | Tropomodulin 3 | 1.4 | 8.6 | 6.2 |
| 1443983_at | Sorbs1 | Sorbin and SH3 domain containing 1 | 1.0 | 5.6 | 5.4 |
| 1421290_at | Hspb7 | heat shock protein family, member 7 (cardiovascular) | 1.3 | 6.5 | 5.2 |
| 1452879_at | Synpo2 | synaptopodin 2 | 2.3 | 11.1 | 4.9 |
| 1422580_at | Myl4 | myosin, light polypeptide 4 | 21.4 | 99.8 | 4.7 |
| 1447259_at | Ank3 | ankyrin 3, epithelial | -1.1 | 4.3 | 4.6 |
| 1448394_at | Myl2 | myosin, light polypeptide 2, regulatory, cardiac, slow | 77.2 | 340.3 | 4.4 |
| 1434369_a_at | Cryab | crystallin, alpha B | 44.2 | 183.9 | 4.2 |
| 1450813_a_at | Tnni1 | troponin I, skeletal, slow 1 | 69.9 | 273.6 | 3.9 |
| 1450732_a_at | Bicd2 | bicaudal D homolog 2 (Drosophila) | 3.2 | 11.3 | 3.6 |
| 1452265_at | Clasp1 | CLIP associating protein 1 | 2.1 | 7.0 | 3.3 |
| 1436042_at | Tln1 | talin 1 | 3.6 | 12.0 | 3.3 |
| 1418258_s_at | Dynll2 | dynein light chain LC8-type 2 | 1.8 | 5.7 | 3.2 |
| 1431035_at | Daam1 | dishevelled associated activator of morphogenesis 1 | 1.8 | 5.8 | 3.2 |
| 1435807_at | Cdc42 | cell division cycle 42 homolog (S. cerevisiae) | 2.1 | 6.4 | 3.1 |
| 1449071_at | Myl7 | myosin, light polypeptide 7, regulatory | 79.7 | 247.1 | 3.1 |
| 1418370_at | Tnnc1 | troponin C, cardiac/slow skeletal | 179.2 | 561.2 | 3.1 |
| 1436678_at | Sgcb | Sarcoglycan, beta (dystrophin-associated glycoprotein) | 4.3 | 12.6 | 3.0 |
| 1460694_s_at | Svil | supervillin | 1.4 | 4.0 | 2.9 |
| 1424746_at | Kif1c | Kinesin family member 1C | 2.2 | 6.0 | 2.8 |
| 1435189_at | Frmpd1 | FERM and PDZ domain containing 1 | 1.2 | 3.1 | 2.6 |
| 1419835_s_at | Plec1 | plectin 1 | -1.1 | 2.5 | 2.6 |
| 1436695_x_at | Rbed1 | RNA binding motif and ELMO domain 1 | -1.0 | 2.5 | 2.6 |
| 1425270_at | Kif1b | kinesin family member 1B | 2.3 | 5.7 | 2.5 |
| 1419668_at | Sgcb | sarcoglycan, beta (dystrophin-associated glycoprotein) | 5.2 | 11.9 | 2.3 |
| 1425677_a_at | Ank1 | ankyrin 1, erythroid | -1.0 | 2.1 | 2.2 |
| 1452166_a_at | Krt1-10 | keratin complex 1, acidic, gene 10 | 1.4 | 3.1 | 2.2 |
| 1449997_at | Tpm3 | tropomyosin 3, gamma | 1.2 | 2.6 | 2.2 |
| 1426778_at | Dag1 | dystroglycan 1 | 1.4 | 2.9 | 2.1 |
| 1447812_x_at | Flnc | filamin C, gamma (actin binding protein 280) | 8.1 | 16.6 | 2.1 |
| 1440990_at | 4832420M10 | hypothetical protein 4832420M10 | 13.6 | 27.8 | 2.0 |

Genes belonging to the GOTERM_CC, “MYOFIBRIL, STRIATED MUSCLE THIN FILAMENT, ACTIN CYTOSKELETON”, “CYTOSKELETON”, “MYOSIN” and GOTERM_BP “CYTOSKELETON ORGANIZATION AND BIOGENESIS” that are upregulated in the 15 days α-MHC^+^ cardiomyocytes (intersection of upregulation in α-MHC^+^ cardiomyocytes (2-fold, t-test p-value < 0.01) compared to control cells in the 15-days old EBs (d15) and to undifferentiated α-MHC ES cells (d0)).

Fold changes (fc) are given for pairwise comparisons between undifferentiated α-MHC ES cells (d0) and day 15 control EBs (d15), between undifferentiated α-MHC ES cells (d0) and 15 day old α-MHC^+^ cardiomyocytes (α-MHC^+^) as well as between day 15 control EBs (d15) and 15 day old α-MHC^+^ cardiomyocytes (α-MHC^+^).

**Additional Data file 5b**

| Probe sets | Symbol | Title | **fc d0**  **vs. d15** | **fc d0 vs. αMHC^+^** | **fc d15 vs. αMHC^+^** |
| --- | --- | --- | --- | --- | --- |
| 1436043_at | Scn7a | sodium channel, voltage-gated, type VII, alpha | 1.5 | 35.6 | 24.3 |
| 1450754_at | Cacna2d2 | calcium channel, voltage-dependent, alpha 2/delta subunit 2 | 2.0 | 39.3 | 20.0 |
| 1450193_at | Hcn1 | hyperpolarization-activated, cyclic nucleotide-gated K+ 1 | 2.2 | 39.6 | 18.0 |
| 1421297_a_at | Cacna1c | calcium channel, voltage-dependent, L type, alpha 1C subunit | 2.2 | 16.4 | 7.6 |
| 1417416_at | Kcna1 | potassium voltage-gated channel, shaker-related subfamily, member 1 | 2.2 | 13.2 | 6.1 |
| 1422834_at | Kcnd2 | potassium voltage-gated channel, Shal-related family, member 2 | -1.0 | 5.1 | 5.2 |
| 1449999_a_at | Cacna2d1 | calcium channel, voltage-dependent, alpha2/delta subunit 1 | 1.2 | 5.7 | 4.6 |
| 1455374_at | Kcnj3 | Potassium inwardly-rectifying channel, subfamily J, member 3 | 1.5 | 6.1 | 4.0 |
| 1438613_at | Kcna4 | potassium voltage-gated channel, shaker-related subfamily, member 4 | 1.1 | 3.3 | 3.2 |
| 1422710_a_at | Cacna1h | calcium channel, voltage-dependent, T type, alpha 1H subunit | 5.8 | 17.3 | 3.0 |
| 1423365_at | Cacna1g | calcium channel, voltage-dependent, T type, alpha 1G subunit | 1.8 | 4.9 | 2.7 |
| 1446632_at | Cacnb2 | Calcium channel, voltage-dependent, beta 2 subunit (Cacnb2), mRNA | 1.2 | 2.8 | 2.4 |
| 1449421_a_at | Kcne2 | potassium voltage-gated channel, Isk-related subfamily, gene 2 | -1.0 | 2.2 | 2.2 |
| 1458381_at | Clic5 | chloride intracellular channel 5 | 1.2 | 2.8 | 2.2 |

Genes belonging to the GOTERM_MF “VOLTAGE-GATED ION CHANNEL ACTIVITY” that are upregulated in the 15 days α-MHC^+^ cardiomyocytes (intersection of upregulation in α-MHC^+^ cardiomyocytes (2-fold, t-test p-value < 0.01) compared to control cells in the 15-days old EBs (d15) and to undifferentiated α-MHC ES cells (d0)).

Fold changes (fc) are given for pairwise comparisons between undifferentiated α-MHC ES cells (d0) and day 15 control EBs (d15), between undifferentiated α-MHC ES cells (d0) and 15 day old α-MHC^+^ cardiomyocytes (α-MHC^+^) as well as between day 15 control EBs (d15) and 15 day old α-MHC^+^ cardiomyocytes (α-MHC^+^).
