# Supplementary material for: Global transcriptome analysis of murine embryonic stem cell-derived cardiomyocytes
Source: Genome Biol. 2007 Apr 11;8(4):R56. doi: 10.1186/gb-2007-8-4-r56 (PMC1896009; doi:10.1186/gb-2007-8-4-r56)
Supplement: Additional data file 6 — Part a provides genes belonging to the KEGG pathway 'oxidative phosphorylation' that are upregulated in α-MHC+ cardiomyocytes (intersection of upregulation in α-MHC+ cardiomyocytes [twofold, t-test P value < 0.01] compared with control cells in the 15-day-old EBs and compared with undifferentiated α-MHC ES cells) and a schematic of the KEGG oxidative phosphorylation pathway. Part b provides the genes belonging to the GOTERM_CC_5 categories 'mitochondrion', 'mitochondrial membrane' and 'mitochondrial electron transport chain', and GOTERM_MF_5 categories 'hydrogen ion transporter activity', 'NADH dehydrogenase (quinone) activity' and 'sodium ion transporter activity' that are upregulated in α-MHC+ cardiomyocytes (intersection of upregulation in α-MHC+ cardiomyocytes [twofold, t-test P value < 0.01] compared with control cells in the 15-day-old EBs and compared with undifferentiated α-MHC ES cells). Part c provides genes belonging to the GOTERM_CC category "fatty acid metabolism" that are upregulated in the α-MHC+ cardiomyocytes (intersection of upregulation in α-MHC+ cardiomyocytes [twofold, t-test P value < 0.01] compared with control cells in the 15-day-old EBs and compared with undifferentiated α-MHC ES cells). [file gb-2007-8-4-r56-S6.doc]

**Additional Data file 6a**

| **Probe Set** | **Symbol** | Title | **fc d0**  **vs. d15** | **fc d0 vs. αMHC^+^** | **fc d15 vs. αMHC^+^** |
| --- | --- | --- | --- | --- | --- |
| 1449218_at | Cox8b | cytochrome c oxidase, subunit VIIIb | 1.6 | 27.9 | 17.1 |
| 1457633_x_at | Cox6a2 | cytochrome c oxidase, subunit VI a, polypeptide 2 | 9.8 | 93.7 | 9.6 |
| 1418709_at | Cox7a1 | cytochrome c oxidase, subunit VIIa 1 | 1.5 | 9.4 | 6.3 |
| 1417286_at | Ndufa5 | NADH dehydrogenase (ubiquinone) 1 alpha subcomplex, 5 | 1.2 | 4.4 | 3.6 |
| 1434053_x_at | Atp5k | ATP synthase, H+ transporting, mitochondrial F1F0 complex, subunit e | 1.3 | 3.9 | 3.0 |
| 1437164_x_at | Atp5o | ATP synthase, H+ transporting, mitochondrial F1 complex, O subunit; similar to ATP5o | -1.0 | 2.9 | 2.9 |
| 1442216_at | Atp6v0d1 | ATPase, H+ transporting, V0 subunit D isoform 1 | 1.0 | 2.9 | 2.9 |
| 1448292_at | Uqcr | ubiquinol-cytochrome c reductase (6.4kD) subunit | 1.2 | 3.5 | 2.8 |
| 1436803_a_at | Ndufb9 | NADH dehydrogenase (ubiquinone) 1 beta subcomplex, 9 | -1.2 | 2.4 | 2.8 |
| 1448934_at | Ndufa10 | NADH dehydrogenase (ubiquinone) 1 alpha subcomplex 10 | 1.0 | 2.7 | 2.7 |
| 1423907_a_at | Ndufs8 | NADH dehydrogenase (ubiquinone) Fe-S protein 8 | 1.2 | 3.0 | 2.6 |
| 1424313_a_at | Ndufs7 | NADH dehydrogenase (ubiquinone) Fe-S protein 7 | 1.1 | 2.9 | 2.6 |
| 1455806_x_at | Ndufa12 | NADH dehydrogenase (ubiquinone) 1 alpha subcomplex, 12 | 1.2 | 3.1 | 2.5 |
| 1428322_a_at | Ndufb10 | NADH dehydrogenase (ubiquinone) 1 beta subcomplex, 10 | -1.0 | 2.3 | 2.4 |
| 1422241_a_at | Ndufa1 | NADH dehydrogenase (ubiquinone) 1 alpha subcomplex, 1 | -1.1 | 2.3 | 2.4 |
| 1448198_a_at | Ndufb8 | NADH dehydrogenase (ubiquinone) 1 beta subcomplex 8 | 1.1 | 2.5 | 2.4 |
| 1423692_at | Ndufa8 | NADH dehydrogenase (ubiquinone) 1 alpha subcomplex, 8 | 1.4 | 3.2 | 2.3 |
| 1438166_x_at | Ndufs4 | NADH dehydrogenase (ubiquinone) Fe-S protein 4 | -1.1 | 2.2 | 2.3 |
| 1416417_a_at | Ndufb7 | NADH dehydrogenase (ubiquinone) 1 beta subcomplex, 7 | 1.4 | 3.1 | 2.2 |
| 1435757_a_at | Uqcrc2 | ubiquinol cytochrome c reductase core protein 2 | 1.1 | 2.4 | 2.2 |
| 1416494_at | Ndufs5 | NADH dehydrogenase (ubiquinone) Fe-S protein 5 | -1.0 | 2.1 | 2.1 |
| 1417102_a_at | Ndufb5 | NADH dehydrogenase (ubiquinone) 1 beta subcomplex, 5 | 1.0 | 2.1 | 2.1 |
| 1417799_at | Atp6v1g2 | ATPase, H+ transporting, V1 subunit G isoform 2 | 1.1 | 2.3 | 2.1 |
| 1447919_x_at | Ndufab1 | NADH dehydrogenase (ubiquinone) 1, alpha/beta subcomplex, 1 | 1.1 | 2.3 | 2.1 |
| 1448284_a_at | Ndufc1 | NADH dehydrogenase (ubiquinone) 1, subcomplex unknown, 1 | 1.2 | 2.4 | 2.0 |

Genes belonging to the KEGG pathway “Oxidative Phosphorylation” that are upregulated in α-MHC^+^ cardiomyocytes (intersection of upregulation in α-MHC^+^ cardiomyocytes (2-fold, t-test p-value < 0.01) compared to control cells in the 15-days old EBs (d15) and to undifferentiated α-MHC ES cells (d0)).

Fold changes (fc) are given for pairwise comparisons between undifferentiated α-MHC ES cells (d0) and day 15 control EBs (d15), between undifferentiated α-MHC ES cells (d0) and 15 day old α-MHC^+^ cardiomyocytes (α-MHC^+^) as well as between day 15 control EBs (d15) and 15 day old α-MHC^+^ cardiomyocytes (α-MHC^+^).

Schematic of the KEGG Oxidative Phosphorylation pathway indicating the upregulated genes (labelled with red background and white letters).

**Additional Data file 6b**

| Probe Set | Symbol | Title | **fc d0**  **vs. d15** | **fc d0 vs. αMHC^+^** | **fc d15 vs. αMHC^+^** |
| --- | --- | --- | --- | --- | --- |
| 1449218_at | Cox8b | cytochrome c oxidase, subunit VIIIb | 1.6 | 27.9 | 17.1 |
| 1420362_a_at | Biklk | Bcl2-interacting killer-like | 2.4 | 32.6 | 13.7 |
| 1457633_x_at | Cox6a2 | cytochrome c oxidase, subunit VI a, polypeptide 2 | 9.8 | 93.7 | 9.6 |
| 1457031_at | Fsd2 | fibronectin type III and SPRY domain containing 2 | 1.5 | 14.1 | 9.5 |
| 1418709_at | Cox7a1 | cytochrome c oxidase, subunit VIIa 1 | 1.5 | 9.4 | 6.3 |
| 1419687_at | Lrp16 | Lrp16 ortholog (human) | -1.2 | 4.9 | 6.0 |
| 1441937_s_at | Pink1 | PTEN induced putative kinase 1 | 1.1 | 5.3 | 4.7 |
| 1440989_at | Mrpl35 | Mitochondrial ribosomal protein L35 | 1.0 | 4.7 | 4.7 |
| 1448381_at | Gfm1 | G elongation factor 1 | -2.1 | 2.1 | 4.4 |
| 1434314_s_at | Rab11fip5 | RAB11 family interacting protein 5 (class I) | 1.2 | 5.3 | 4.3 |
| 1443822_s_at | Zcd1 | Zinc finger, CDGSH-type domain 1 | 1.2 | 5.1 | 4.1 |
| 1417008_at | Crat | carnitine acetyltransferase | 1.4 | 5.1 | 3.7 |
| 1448131_at | Mfn2 | mitofusin 2 | 2.2 | 8.1 | 3.7 |
| 1452005_at | Dlat | dihydrolipoamide S-acetyltransferase (E2 component of pyruvate dehydrogenase complex) | -1.3 | 2.9 | 3.7 |
| 1422484_at | Cycs | cytochrome c, somatic | 1.5 | 5.3 | 3.6 |
| 1449000_at | D10Jhu81e | DNA segment, Chr 10, Johns Hopkins University 81 expressed | 1.1 | 3.9 | 3.6 |
| 1459793_s_at | Kras | v-Ki-ras2 Kirsten rat sarcoma viral oncogene homolog | -1.6 | 2.2 | 3.6 |
| 1417286_at | Ndufa5 | NADH dehydrogenase (ubiquinone) 1 alpha subcomplex, 5 | 1.2 | 4.4 | 3.6 |
| 1418996_a_at | Lyrm5 | LYR motif containing 5 | 1.0 | 3.6 | 3.5 |
| 1417273_at | Pdk4 | pyruvate dehydrogenase kinase, isoenzyme 4 | 1.2 | 3.9 | 3.3 |
| 1426856_at | Hsdl2 | hydroxysteroid dehydrogenase like 2 | 1.8 | 5.7 | 3.2 |
| 1417970_at | Atp5s | ATP synthase, H+ transporting, mitochondrial F0 complex, subunit s | -1.4 | 2.3 | 3.2 |
| 1436934_s_at | Aco2 | aconitase 2, mitochondrial | 1.1 | 3.4 | 3.2 |
| 1434657_at | Gls | Glutaminase | 1.8 | 5.6 | 3.1 |
| 1434053_x_at | Atp5k | ATP synthase, H+ transporting, mitochondrial F1F0 complex, subunit e | 1.3 | 3.9 | 3.0 |
| 1423833_a_at | Brp44 | brain protein 44 | 2.2 | 6.5 | 3.0 |
| 1437164_x_at | Atp5o | ATP synthase, H+ transporting, mitochondrial F1 complex, O subunit; similar to ATP5o | -1.0 | 2.9 | 2.9 |
| 1442216_at | Atp6v0d1 | ATPase, H+ transporting, V0 subunit D isoform 1 | 1.0 | 2.9 | 2.9 |
| 1456573_x_at | Nnt | nicotinamide nucleotide transhydrogenase | -1.0 | 2.8 | 2.9 |
| 1448292_at | Uqcr | ubiquinol-cytochrome c reductase (6.4kD) subunit | 1.2 | 3.5 | 2.8 |
| 1418321_at | Dci | dodecenoyl-Coenzyme A delta isomerase (3,2 trans-enoyl-Coenyme A isomerase) | -1.0 | 2.8 | 2.8 |
| 1436803_a_at | Ndufb9 | NADH dehydrogenase (ubiquinone) 1 beta subcomplex, 9 | -1.2 | 2.4 | 2.8 |
| 1450667_a_at | Cs | citrate synthase | 1.2 | 3.5 | 2.8 |
| 1448934_at | Ndufa10 | NADH dehydrogenase (ubiquinone) 1 alpha subcomplex 10 | 1.0 | 2.7 | 2.7 |
| 1447701_x_at | Idh3a | isocitrate dehydrogenase 3 (NAD+) alpha | -1.3 | 2.0 | 2.6 |
| 1423907_a_at | Ndufs8 | NADH dehydrogenase (ubiquinone) Fe-S protein 8 | 1.2 | 3.0 | 2.6 |
| 1423108_at | Slc25a20 | solute carrier family 25 (mitochondrial carnitine/acylcarnitine translocase), member 20 | 1.2 | 3.2 | 2.6 |
| 1424628_a_at | Ndufv3 | NADH dehydrogenase (ubiquinone) flavoprotein 3, 10kDa | 1.4 | 3.6 | 2.6 |
| 1417316_at | Them2 | thioesterase superfamily member 2 | 1.3 | 3.3 | 2.6 |
| 1424313_a_at | Ndufs7 | NADH dehydrogenase (ubiquinone) Fe-S protein 7 | 1.1 | 2.9 | 2.6 |
| 1455806_x_at | Ndufa12 | NADH dehydrogenase (ubiquinone) 1 alpha subcomplex, 12 | 1.2 | 3.1 | 2.5 |
| 1447720_x_at | Prkaca | Protein kinase, cAMP dependent, catalytic, alpha | 2.1 | 5.3 | 2.5 |
| 1425270_at | Kif1b | kinesin family member 1B | 2.3 | 5.7 | 2.5 |
| 1428322_a_at | Ndufb10 | NADH dehydrogenase (ubiquinone) 1 beta subcomplex, 10 | -1.0 | 2.3 | 2.4 |
| 1419974_at | Scp2 | sterol carrier protein 2, liver | 1.2 | 2.9 | 2.4 |
| 1422241_a_at | Ndufa1 | NADH dehydrogenase (ubiquinone) 1 alpha subcomplex, 1 | -1.1 | 2.3 | 2.4 |
| 1448198_a_at | Ndufb8 | NADH dehydrogenase (ubiquinone) 1 beta subcomplex 8 | 1.1 | 2.5 | 2.4 |
| 1448853_at | Synj2bp | synaptojanin 2 binding protein | -1.1 | 2.2 | 2.3 |
| 1423692_at | Ndufa8 | NADH dehydrogenase (ubiquinone) 1 alpha subcomplex, 8 | 1.4 | 3.2 | 2.3 |
| 1438166_x_at | Ndufs4 | NADH dehydrogenase (ubiquinone) Fe-S protein 4 | -1.1 | 2.2 | 2.3 |
| 1416417_a_at | Ndufb7 | NADH dehydrogenase (ubiquinone) 1 beta subcomplex, 7 | 1.4 | 3.1 | 2.2 |
| 1449181_at | Fech | ferrochelatase | 1.6 | 3.5 | 2.2 |
| 1423738_at | Oxa1l | oxidase assembly 1-like | -1.1 | 2.1 | 2.2 |
| 1426088_at | Nd5 | NADH dehydrogenase subunit 5 | -1.0 | 2.1 | 2.2 |
| 1435864_a_at | LOC67892 | LOC67892 hyp. protein | 1.5 | 3.3 | 2.2 |
| 1435757_a_at | Uqcrc2 | ubiquinol cytochrome c reductase core protein 2 | 1.1 | 2.4 | 2.2 |
| 1416494_at | Ndufs5 | NADH dehydrogenase (ubiquinone) Fe-S protein 5 | -1.0 | 2.1 | 2.1 |
| 1417102_a_at | Ndufb5 | NADH dehydrogenase (ubiquinone) 1 beta subcomplex, 5 | 1.0 | 2.1 | 2.1 |
| 1422998_a_at | Glrx2 | glutaredoxin 2 (thioltransferase) | 1.1 | 2.3 | 2.1 |
| 1417799_at | Atp6v1g2 | ATPase, H+ transporting, V1 subunit G isoform 2 | 1.1 | 2.3 | 2.1 |
| 1447919_x_at | Ndufab1 | NADH dehydrogenase (ubiquinone) 1, alpha/beta subcomplex, 1 | 1.1 | 2.3 | 2.1 |
| 1448284_a_at | Ndufc1 | NADH dehydrogenase (ubiquinone) 1, subcomplex unknown, 1 | 1.2 | 2.4 | 2.0 |
| 1423242_at | Mrps36 | mitochondrial ribosomal protein S36 | 1.3 | 2.7 | 2.0 |
| 1437172_x_at | Hadhb | hydroxyacyl-Coenzyme A dehydrogenase/3-ketoacyl-Coenzyme A thiolase/enoyl-Coenzyme A hydratase (trifunctional protein), beta subunit | 1.4 | 2.8 | 2.0 |

Genes belonging to the GOTERM_CC_5 “MITOCHONDRION”, “MITOCHONDRIAL MEMBRANE”, “MITOCHONDRIAL ELECTRON TRANSPORT CHAIN” and GOTERM_MF_5 “HYDROGEN ION TRANSPORTER ACTIVITY”, “NADH DEHYDROGENASE (QUINONE) ACTIVITY”, “SODIUM ION TRANSPORTER ACTIVITY” that are upregulated in α-MHC^+^ cardiomyocytes (intersection of upregulation in α-MHC^+^ cardiomyocytes (2-fold, t-test p-value < 0.01) compared to control cells in the 15-days old EBs (d15) and to undifferentiated α-MHC ES cells (d0)).

Fold changes (fc) are given for pairwise comparisons between undifferentiated α-MHC ES cells (d0) and day 15 control EBs (d15), between undifferentiated α-MHC ES cells (d0) and 15 day old α-MHC^+^ cardiomyocytes (α-MHC^+^) as well as between day 15 control EBs (d15) and 15 day old α-MHC^+^ cardiomyocytes (α-MHC^+^).

**Additional Data file 6c**

| Probe Set | Symbol | Title | **fc d0**  **vs. d15** | **fc d0 vs. αMHC^+^** | **fc d15 vs. αMHC^+^** |
| --- | --- | --- | --- | --- | --- |
| 1456395_at | Ppargc1a | peroxisome proliferative activated receptor, gamma, coactivator 1 alpha | 3.0 | 33.3 | 11.0 |
| 1434100_x_at | Casp7 | Caspase 7 | 5.0 | 33.7 | 6.8 |
| 1435910_at | Fads3 | Fatty acid desaturase 3 (Fads3), mRNA | 2.9 | 11.1 | 3.9 |
| 1417008_at | Crat | carnitine acetyltransferase | 1.4 | 5.1 | 3.7 |
| 1418321_at | Dci | dodecenoyl-Coenzyme A delta isomerase (3,2 trans-enoyl-Coenyme A isomerase) | -1.0 | 2.8 | 2.8 |
| 1456812_at | Abcd2 | ATP-binding cassette, sub-family D (ALD), member 2 | 1.2 | 3.2 | 2.6 |
| 1419974_at | Scp2 | sterol carrier protein 2, liver | 1.2 | 2.9 | 2.4 |
| 1447919_x_at | Ndufab1 | NADH dehydrogenase (ubiquinone) 1, alpha/beta subcomplex, 1 | 1.1 | 2.3 | 2.1 |
| 1437172_x_at | Hadhb | hydroxyacyl-CoA dehydrogenase/3-ketoacyl-CoA thiolase/enoyl-CoA hydratase (trifunctional protein), beta subunit | 1.4 | 2.8 | 2.0 |

Genes belonging to the GOTERM_CC “Fatty Acid Metabolism” that are up-regulated in the α-MHC^+^ cardiomyocytes (intersection of upregulation in α-MHC^+^ cardiomyocytes (2-fold, t-test p-value < 0.01) compared to control cells in the 15-days old EBs (d15) and to undifferentiated α-MHC ES cells (d0)).

Fold changes (fc) are given for pairwise comparisons between undifferentiated α-MHC ES cells (d0) and day 15 control EBs (d15), between undifferentiated α-MHC ES cells (d0) and 15 day old α-MHC^+^ cardiomyocytes (α-MHC^+^) as well as between day 15 control EBs (d15) and 15 day old α-MHC^+^ cardiomyocytes (α-MHC^+^).
