# Supplementary material for: Global transcriptome analysis of murine embryonic stem cell-derived cardiomyocytes
Source: Genome Biol. 2007 Apr 11;8(4):R56. doi: 10.1186/gb-2007-8-4-r56 (PMC1896009; doi:10.1186/gb-2007-8-4-r56)
Supplement: Additional data file 7 — Part a provides genes belonging to the GOTERM_BP_5 category 'enzyme linked receptor protein signaling pathway' that are upregulated in the α-MHC+ cardiomyocytes (intersection of upregulation in α-MHC+ cardiomyocytes [twofold, t-test P value < 0.01] compared with control cells in the 15-day-old EBs and compared with undifferentiated α-MHC ES cells). Part b provides genes that belong to the GOTERM_MF_5 category 'protein kinase activity' that are upregulated in α-MHC+ cardiomyocytes (intersection of upregulation in α-MHC+ cardiomyocytes [twofold, t-test P value < 0.01] compared with control cells in the 15-day-old EBs and compared with undifferentiated α-MHC ES cells). Part c provides genes belonging to the GOTERM_BP_5 categories 'negative regulation of Wnt receptor signaling pathway' and 'negative regulation of signal transduction' that are upregulated in α-MHC+ cardiomyocytes (intersection of upregulation in α-MHC+ cardiomyocytes [twofold, t-test P value < 0.01] compared with control cells in the 15-day-old EBs and compared with undifferentiated α-MHC ES cells). Part d provides genes belonging to the Biocarta pathway 'p38 mitogen-activated protein kinase signaling" that are upregulated in α-MHC+ cardiomyocytes (intersection of upregulation in α-MHC+ cardiomyocytes [twofold, t-test P value < 0.01] compared with control cells in the 15-day-old EBs and compared with undifferentiated α-MHC ES cells). Part e provides genes belonging to the KEGG pathway 'Calcium Signalling' that are upregulated in α-MHC+ cardiomyocytes (intersection of upregulation in α-MHC+ cardiomyocytes [twofold, t-test P value < 0.01] compared with control cells in the 15-day-old EBs and compared with undifferentiated α-MHC ES cells). Part f provides genes belonging to the GOTERM_BP_5 'Regulation of cell size' that are upregulated in α-MHC+ cardiomyocytes (intersection of upregulation in α-MHC+ cardiomyocytes [twofold, t-test P value < 0.01] compared with control cells in the 15-day-old EBs and compare [file gb-2007-8-4-r56-S7.doc]

**Additional data file 7a**

| Probe Set | Symbol | Title | **fc d0**  **vs. d15** | **fc d0 vs. αMHC^+^** | **fc d15 vs. αMHC^+^** |
| --- | --- | --- | --- | --- | --- |
| 1443187_at | Rspo3 | R-spondin 3 homolog (Xenopus laevis) | 1.4 | 30.5 | 21.7 |
| 1451830_a_at | Spnb2 | spectrin beta 2 | 2.3 | 25.9 | 11.4 |
| 1443983_at | Sorbs1 | Sorbin and SH3 domain containing 1 | 1.0 | 5.6 | 5.4 |
| 1438251_x_at | Htra1 | HtrA serine peptidase 1 | 9.6 | 37.5 | 3.9 |
| 1452380_at | Epha7 | Eph receptor A7 | 5.5 | 20.7 | 3.8 |
| 1421282_at | Bmp5 | bone morphogenetic protein 5 | 1.6 | 4.0 | 2.6 |
| 1442371_at | LOC76908 | hypothetical Receptor tyrosine kinase class II containing protein | -1.0 | 2.4 | 2.5 |
| 1439382_x_at | Ddr1 | discoidin domain receptor family, member 1 | 1.8 | 4.1 | 2.2 |
| 1456482_at | Pik3r3 | phosphatidylinositol 3 kinase, regulatory subunit, polypeptide 3 (p55) | 3.1 | 6.7 | 2.1 |
| 1425983_x_at | Hipk2 | homeodomain interacting protein kinase 2 | 1.1 | 2.2 | 2.0 |
| 1423341_at | Cspg4 | chondroitin sulfate proteoglycan 4 | 1.4 | 2.9 | 2.0 |

Genes belonging to the GOTERM_BP_5, “Enzyme linked receptor protein signaling Pathway” that are upregulated in α-MHC^+^ cardiomyocytes (intersection of upregulation in α-MHC^+^ cardiomyocytes (2-fold, t-test p-value < 0.01) compared to control cells in the 15-days old EBs (d15) and to undifferentiated α-MHC ES cells (d0)).

Fold changes (fc) are given for pairwise comparisons between undifferentiated α-MHC ES cells (d0) and day 15 control EBs (d15), between undifferentiated α-MHC ES cells (d0) and 15 day old α-MHC^+^ cardiomyocytes (α-MHC^+^) as well as between day 15 control EBs (d15) and 15 day old α-MHC^+^ cardiomyocytes (α-MHC^+^).

**Additional data file 7b**

| Probe Set | Symbol | Title | **fc d0**  **vs. d15** | **fc d0 vs. αMHC^+^** | **fc d15 vs. αMHC^+^** |
| --- | --- | --- | --- | --- | --- |
| 1443187_at | Rspo3 | R-spondin 3 homolog (Xenopus laevis) | 1.4 | 30.5 | 21.7 |
| 1447043_at | Erbb4 | v-erb-a erythroblastic leukemia viral oncogene homolog 4 | 1.1 | 14.9 | 13.2 |
| 1439101_at | Mlck | Myosin light chain kinase | 4.5 | 53.5 | 12.0 |
| 1447806_s_at | Stk23 | serine/threonine kinase 23 | 8.0 | 89.2 | 11.2 |
| 1425425_a_at | Wif1 | Wnt inhibitory factor 1 | 1.9 | 17.6 | 9.1 |
| 1452478_at | Alpk2 | alpha-kinase 2 | 2.8 | 23.9 | 8.4 |
| 1434944_at | Dmpk | dystrophia myotonica-protein kinase | 1.8 | 12.9 | 7.0 |
| 1429463_at | Prkaa2 | protein kinase, AMP-activated, alpha 2 catalytic subunit | 1.2 | 7.7 | 6.2 |
| 1434766_at | Prkaa2 | Protein kinase, AMP-activated, alpha 2 catalytic subunit (Prkaa2), mRNA | 1.1 | 5.3 | 4.9 |
| 1441937_s_at | Pink1 | PTEN induced putative kinase 1 | 1.1 | 5.3 | 4.7 |
| 1425968_s_at | Apeg1 | aortic preferentially expressed gene 1 | 1.5 | 6.4 | 4.3 |
| 1452380_at | Epha7 | Eph receptor A7 | 5.5 | 20.7 | 3.8 |
| 1439168_at | Camk2d | calcium/calmodulin-dependent protein kinase II, delta | 1.6 | 6.1 | 3.7 |
| 1447623_s_at | Prkcm | Protein kinase C mu type | 14.5 | 49.3 | 3.4 |
| 1417273_at | Pdk4 | pyruvate dehydrogenase kinase, isoenzyme 4 | 1.2 | 3.9 | 3.3 |
| 1455703_at | Akt2 | thymoma viral proto-oncogene 2 | -1.3 | 2.3 | 2.9 |
| 1435746_at | Srpk2 | serine/arginine-rich protein specific kinase 2 | -1.2 | 2.4 | 2.9 |
| 1421340_at | Map3k5 | mitogen activated protein kinase kinase kinase 5 | 1.0 | 2.9 | 2.8 |
| 1457311_at | Camk2a | calcium/calmodulin-dependent protein kinase II alpha | 1.2 | 3.2 | 2.7 |
| 1447720_x_at | Prkaca | Protein kinase, cAMP dependent, catalytic, alpha | 2.1 | 5.3 | 2.5 |
| 1442371_at | LOC76908 | hypothetical Receptor tyrosine kinase class II containing protein | -1.0 | 2.4 | 2.5 |
| 1428783_at | Prkar2a | protein kinase, cAMP dependent regulatory, type II alpha | 1.5 | 3.5 | 2.4 |
| 1439382_x_at | Ddr1 | discoidin domain receptor family, member 1 | 1.8 | 4.1 | 2.2 |
| 1449630_s_at | Mark1 | MAP/microtubule affinity-regulating kinase 1 | 5.6 | 12.2 | 2.2 |
| 1451736_a_at | Map2k7 | mitogen activated protein kinase kinase 7 | -1.0 | 2.0 | 2.1 |
| 1425983_x_at | Hipk2 | homeodomain interacting protein kinase 2 | 1.1 | 2.2 | 2.0 |

Genes belonging to the GOTERM_MF_5, “PROTEIN KINASE ACTIVITY” that are up-regulated in α-MHC^+^ cardiomyocytes (intersection of upregulation in α-MHC^+^ cardiomyocytes (2-fold, t-test p-value < 0.01) compared to control cells in the 15-days old EBs (d15) and to undifferentiated α-MHC ES cells (d0)).

Fold changes (fc) are given for pairwise comparisons between undifferentiated α-MHC ES cells (d0) and day 15 control EBs (d15), between undifferentiated α-MHC ES cells (d0) and 15 day old α-MHC^+^ cardiomyocytes (α-MHC^+^) as well as between day 15 control EBs (d15) and 15 day old α-MHC^+^ cardiomyocytes (α-MHC^+^).

**Additional data file 7c**

| **Probesets** | **Symbol** | **Title** | **fc d0 vs. d15** | **fc d0 vs. αMHC^+^** | **fc d15 vs. αMHC^+^** |
| --- | --- | --- | --- | --- | --- |
| 1425425_a_at | Wif1 | Wnt inhibitory factor 1 | 1.9 | 17.6 | 9.1 |
| 1444409_at | Rph3al | Noc2 | 1.9 | 11.8 | 6.2 |
| 1448669_at | Dkk3 | dickkopf homolog 3 (Xenopus laevis) | 13.9 | 57.2 | 4.1 |
| 1416749_at | Htra1 | HtrA serine peptidase 1 | 11.8 | 44.6 | 3.8 |
| 1437351_at | Cxxc4 | CXXC finger 4 | 4.9 | 10.3 | 2.1 |

Genes belonging to the GOTERMs_BP_5 “NEGATIVE REGULATION OF WNT RECEPTOR SIGNALING PATHWAY” and “NEGATIVE REGULATION OF SIGNAL TRANSDUCTION” that are up-regulated in α-MHC^+^ cardiomyocytes (intersection of upregulation in α-MHC^+^ cardiomyocytes (2-fold, t-test p-value < 0.01) compared to control cells in the 15-days old EBs (d15) and to undifferentiated α-MHC ES cells (d0)).

Fold changes (fc) are given for pairwise comparisons between undifferentiated α-MHC ES cells (d0) and day 15 control EBs (d15), between undifferentiated α-MHC ES cells (d0) and 15 day old α-MHC^+^ cardiomyocytes (α-MHC^+^) as well as between day 15 control EBs (d15) and 15 day old α-MHC^+^ cardiomyocytes (α-MHC^+^).

**Additional data file 7d**

| Probesets | Symbol | Title | **fc d0 vs. d15** | **fc d0 vs. αMHC^+^** | **fc d15 vs. αMHC^+^** |
| --- | --- | --- | --- | --- | --- |
| 1427186_a_at | Mef2a | myocyte enhancer factor 2A | 9.3 | 46.2 | 5.0 |
| 1429888_a_at | Hspb2 | heat shock protein 2 | 1.1 | 4.6 | 4.4 |
| 1435807_at | Cdc42 | cell division cycle 42 homolog (S. cerevisiae) | 2.1 | 6.4 | 3.1 |
| 1421340_at | Map3k5 | mitogen activated protein kinase kinase kinase 5 | 1.0 | 2.9 | 2.8 |
| 1443897_at | Ddit3 | DNA-damage inducible transcript 3 | 1.6 | 3.5 | 2.2 |

Genes belonging to the Biocarta Pathway, “p38 MAPK signalling” that are up-regulated in α-MHC^+^ cardiomyocytes (intersection of upregulation in α-MHC^+^ cardiomyocytes (2-fold, t-test p-value < 0.01) compared to control cells in the 15-days old EBs (d15) and to undifferentiated α-MHC ES cells (d0)).

Fold changes (fc) are given for pairwise comparisons between undifferentiated α-MHC ES cells (d0) and day 15 control EBs (d15), between undifferentiated α-MHC ES cells (d0) and 15 day old α-MHC^+^ cardiomyocytes (α-MHC^+^) as well as between day 15 control EBs (d15) and 15 day old α-MHC^+^ cardiomyocytes (α-MHC^+^).

**Additional data file 7e**

| **Probesets** | **Symbol** | **Title** | **fc d0**  **vs. d15** | **fc d0 vs. αMHC^+^** | **fc d15 vs. αMHC^+^** |
| --- | --- | --- | --- | --- | --- |
| 1450123_at | Ryr2 | ryanodine receptor 2, cardiac | 4.8 | 71.8 | 14.8 |
| 1437675_at | Slc8a1 | solute carrier family 8 (sodium/calcium exchanger), member 1 | 5.7 | 83.5 | 14.6 |
| 1447043_at | Erbb4 | PREDICTED: v-erb-a erythroblastic leukemia viral oncogene homolog 4 [Mus musculus], mRNA sequence | 1.1 | 14.9 | 13.2 |
| 1423359_at | Pln | phospholamban | 11.7 | 147.4 | 12.6 |
| 1439101_at | Mlck | Myosin light chain kinase | 4.5 | 53.5 | 12.0 |
| 1426032_at | Nfatc2 | nuclear factor of activated T-cells, cytoplasmic, calcineurin-dependent 2 | 1.0 | 8.9 | 8.5 |
| 1421297_a_at | Cacna1c | calcium channel, voltage-dependent, L type, alpha 1C subunit | 2.2 | 16.4 | 7.6 |
| 1423420_at | Adrb1 | adrenergic receptor, beta 1 | 1.8 | 8.7 | 5.0 |
| 1439168_at | Camk2d | calcium/calmodulin-dependent protein kinase II, delta | 1.6 | 6.1 | 3.7 |
| 1452363_a_at | Atp2a2 | ATPase, Ca++ transporting, cardiac muscle, slow twitch 2 | 6.7 | 24.5 | 3.7 |
| 1418370_at | Tnnc1 | troponin C, cardiac/slow skeletal | 179.2 | 561.2 | 3.1 |
| 1422710_a_at | Cacna1h | calcium channel, voltage-dependent, T type, alpha 1H subunit | 5.8 | 17.3 | 3.0 |
| 1418586_at | Adcy9 | adenylate cyclase 9 | -1.0 | 2.7 | 2.8 |
| 1423365_at | Cacna1g | calcium channel, voltage-dependent, T type, alpha 1G subunit | 1.8 | 4.9 | 2.7 |
| 1457311_at | Camk2a | calcium/calmodulin-dependent protein kinase II alpha | 1.2 | 3.2 | 2.7 |
| 1447720_x_at | Prkaca | Protein kinase, cAMP dependent, catalytic, alpha | 2.1 | 5.3 | 2.5 |
| 1442021_at | Gnal | guanine nucleotide binding protein, alpha stimulating, olfactory type | 1.1 | 2.4 | 2.2 |

Genes belonging to the KEGG Pathway, “Calcium Signalling” that are upregulated in α-MHC^+^ cardiomyocytes (intersection of upregulation in α-MHC^+^ cardiomyocytes (2-fold, t-test p-value < 0.01) compared to control cells in the 15-days old EBs (d15) and to undifferentiated α-MHC ES cells (d0)).

Fold changes (fc) are given for pairwise comparisons between undifferentiated α-MHC ES cells (d0) and day 15 control EBs (d15), between undifferentiated α-MHC ES cells (d0) and 15 day old α-MHC^+^ cardiomyocytes (α-MHC^+^) as well as between day 15 control EBs (d15) and 15 day old α-MHC^+^ cardiomyocytes (α-MHC^+^).

Schematic of the Calcium Signaling pathway indicating the upregulated genes (labelled with red background and white letters).

Schematic of the Calcium Signaling pathway indicating the upregulated genes (labelled with red background and white letters).

**Additional data file 7f**

| **Probesets** | **Symbol** | **Title** | **fc d0**  **vs. d15** | **fc d0 vs. αMHC^+^** | **fc d15 vs. αMHC^+^** |
| --- | --- | --- | --- | --- | --- |
| 1425978_at | Myocd | myocardin | 12.2 | 116.4 | 9.6 |
| 1460591_at | Esr1 | estrogen receptor 1 (alpha) | 1.1 | 9.5 | 8.8 |
| 1455901_at | Chpt1 | choline phosphotransferase 1 | 3.6 | 16.4 | 4.5 |
| 1416749_at | Htra1 | HtrA serine peptidase 1 | 11.8 | 44.6 | 3.8 |
| 1448724_at | Cish | cytokine inducible SH2-containing protein | 1.9 | 5.6 | 3.0 |
| 1446141_at | Tgfb2 | Transforming growth factor, beta 2 | 1.1 | 2.9 | 2.6 |
| 1452114_s_at | Igfbp5 | insulin-like growth factor binding protein 5 | 113.3 | 283.6 | 2.5 |

Genes belonging to the GOTERM_BP_5, “REGULATION OF CELL SIZE” that are upregulated in α-MHC^+^ cardiomyocytes (intersection of upregulation in α-MHC^+^ cardiomyocytes (2-fold, t-test p-value < 0.01) compared to control cells in the 15-days old EBs (d15) and to undifferentiated α-MHC ES cells (d0)).

Fold changes (fc) are given for pairwise comparisons between undifferentiated α-MHC ES cells (d0) and day 15 control EBs (d15), between undifferentiated α-MHC ES cells (d0) and 15 day old α-MHC^+^ cardiomyocytes (α-MHC^+^) as well as between day 15 control EBs (d15) and 15 day old α-MHC^+^ cardiomyocytes (α-MHC^+^).
