# Supplementary material for: Global transcriptome analysis of murine embryonic stem cell-derived cardiomyocytes
Source: Genome Biol. 2007 Apr 11;8(4):R56. doi: 10.1186/gb-2007-8-4-r56 (PMC1896009; doi:10.1186/gb-2007-8-4-r56)
Supplement: Additional data file 8 — Provided are lists of probe sets for the subclusters A, B, and C, as identified in the hierarchical clustering of probe sets downregulated in α-MHC+ cells (Figure 6). Probe sets are listed with the corresponding gene symbol and gene title. [file gb-2007-8-4-r56-S8.doc]

**Additional data file 8**

#### Subcluster A

| **Probe sets** | **symbol** | **Titel** | **fc d0**  **vs. d15** | **fc d0**  **vs. αMHC^+^** | **fc d15**  **vs. αMHC^+^** |
| --- | --- | --- | --- | --- | --- |
| 1424046_at | Bub1 | budding uninhibited by benzimidazoles 1 homolog (S. cerevisiae) | -2.6 | -51.0 | -19.7 |
| 1454159_a_at | Igfbp2 | insulin-like growth factor binding protein 2 | -1.6 | -23.9 | -14.8 |
| 1437752_at | Lin28 | lin-28 homolog (C. elegans) | -2.4 | -34.1 | -14.4 |
| 1417019_a_at | Cdc6 | cell division cycle 6 homolog (S. cerevisiae) | -2.6 | -35.8 | -13.9 |
| 1451128_s_at | Kif22 | kinesin family member 22 | -3.0 | -40.4 | -13.5 |
| 1424629_at | Brca1 | breast cancer 1 | -3.7 | -50.0 | -13.4 |
| 1437033_a_at | Skp2 | S-phase kinase-associated protein 2 (p45) | -1.3 | -17.7 | -13.1 |
| 1435306_a_at | Kif11 | kinesin family member 11 | -2.7 | -34.2 | -12.9 |
| 1459211_at | Gli2 | GLI-Kruppel family member GLI2 | -2.3 | -29.2 | -12.5 |
| 1451064_a_at | Psat1 | phosphoserine aminotransferase 1 | -3.3 | -40.6 | -12.2 |
| 1416309_at | Nusap1 | nucleolar and spindle associated protein 1 | -1.7 | -20.7 | -12.1 |
| 1439510_at | Sgol1 | shugoshin-like 1 (S. pombe) | -3.5 | -40.0 | -11.4 |
| 1425926_a_at | Otx2 | orthodenticle homolog 2 (Drosophila) | -2.0 | -22.2 | -11.3 |
| 1454694_a_at | Top2a | topoisomerase (DNA) II alpha | -1.7 | -19.1 | -11.3 |
| 1449171_at | Ttk | Ttk protein kinase | -2.4 | -27.3 | -11.2 |
| 1428104_at | Tpx2 | TPX2, microtubule-associated protein homolog (Xenopus laevis) | -1.6 | -18.3 | -11.1 |
| 1424991_s_at | Tyms; Tyms-ps | thymidylate synthase; thymidylate synthase, pseudogene | -1.5 | -16.5 | -10.9 |
| 1421346_a_at | Slc6a6 | solute carrier family 6 (neurotransmitter transporter, taurine), member 6 | -1.9 | -19.7 | -10.4 |
| 1448627_s_at | Pbk | PDZ binding kinase | -1.3 | -12.8 | -9.8 |
| 1436000_a_at | Skp2 | S-phase kinase-associated protein 2 (p45) | -1.3 | -12.3 | -9.8 |
| 1419838_s_at | Plk4 | polo-like kinase 4 (Drosophila) | -1.4 | -13.3 | -9.6 |
| 1426652_at | Mcm3 | minichromosome maintenance deficient 3 (S. cerevisiae) | -3.0 | -27.0 | -9.2 |
| 1426246_at | Pros1 | protein S (alpha) | -1.6 | -14.4 | -9.1 |
| 1417400_at | Rai14 | retinoic acid induced 14 | -1.6 | -14.6 | -9.1 |
| 1460247_a_at | Skp2 | S-phase kinase-associated protein 2 (p45) | -1.2 | -10.3 | -8.9 |
| 1434695_at | Dtl | denticleless homolog (Drosophila) | -2.1 | -19.0 | -8.9 |
| 1426002_a_at | Cdc7 | cell division cycle 7 (S. cerevisiae) | -1.2 | -10.4 | -8.8 |
| 1434789_at | Depdc1b | DEP domain containing 1B | -2.5 | -21.7 | -8.7 |
| 1420028_s_at | Mcm3 | minichromosome maintenance deficient 3 (S. cerevisiae) | -2.5 | -20.6 | -8.4 |
| 1424278_a_at | Birc5 | baculoviral IAP repeat-containing 5 | -1.8 | -15.2 | -8.3 |
| 1428142_at | Etv5 | ets variant gene 5 | -6.9 | -57.3 | -8.3 |
| 1417450_a_at | Tacc3 | transforming, acidic coiled-coil containing protein 3 | -1.7 | -13.7 | -8.3 |
| 1428069_at | Cdca7 | cell division cycle associated 7 | -2.5 | -20.4 | -8.1 |
| 1428480_at | Cdca8 | cell division cycle associated 8 | -1.7 | -13.6 | -8.1 |
| 1421546_a_at | Racgap1 | Rac GTPase-activating protein 1 | -1.4 | -11.2 | -7.7 |
| 1422814_at | Aspm | asp (abnormal spindle)-like, microcephaly associated (Drosophila) | -2.3 | -17.5 | -7.7 |
| 1449207_a_at | Kif20a | kinesin family member 20A | -1.6 | -12.5 | -7.6 |
| 1416251_at | Mcm6 | minichromosome maintenance deficient 6 (MIS5 homolog, S. pombe) | -2.4 | -18.1 | -7.4 |
| 1429658_a_at | Smc2l1 | SMC2 structural maintenance of chromosomes 2-like 1 (yeast) | -2.2 | -16.0 | -7.4 |
| 1438009_at | Hist1h2ad | Histone 1, H2ae | -1.8 | -13.1 | -7.4 |
| 1422430_at | Fignl1 | fidgetin-like 1 | -2.3 | -17.1 | -7.3 |
| 1428105_at | Tpx2 | TPX2, microtubule-associated protein homolog (Xenopus laevis) | -3.5 | -25.3 | -7.3 |
| 1439208_at | Chek1 | checkpoint kinase 1 homolog (S. pombe) | -1.9 | -13.8 | -7.2 |
| 1424556_at | Pycr1 | pyrroline-5-carboxylate reductase 1 | -2.2 | -15.8 | -7.2 |
| 1433885_at | Iqgap2 | IQ motif containing GTPase activating protein 2 | -1.4 | -10.0 | -7.1 |
| 1428304_at | Esco2 | establishment of cohesion 1 homolog 2 (S. cerevisiae) | -9.4 | -66.8 | -7.1 |
| 1452954_at | Ube2c | ubiquitin-conjugating enzyme E2C | -1.8 | -12.6 | -7.1 |
| 1456280_at | Clspn | claspin homolog (Xenopus laevis) | -1.5 | -10.4 | -7.0 |
| 1422513_at | Ccnf | cyclin F | -2.7 | -18.6 | -7.0 |
| 1448191_at | Plk1 | polo-like kinase 1 (Drosophila) | -1.9 | -13.4 | -7.0 |
| 1455795_at | Sart2 | squamous cell carcinoma antigen recognized by T cells 2 | -1.8 | -12.6 | -7.0 |
| 1423775_s_at | Prc1 | protein regulator of cytokinesis 1 | -1.8 | -12.7 | -7.0 |
| 1448126_at | MGI:1929091 | teratocarcinoma expressed, serine rich | -3.0 | -20.6 | -6.9 |
| 1451015_at | Tkt | transketolase | -1.8 | -12.6 | -6.9 |
| 1422016_a_at | Cenph | centromere autoantigen H | -2.9 | -19.4 | -6.7 |
| 1437580_s_at | Nek2 | NIMA (never in mitosis gene a)-related expressed kinase 2 | -2.6 | -17.3 | -6.6 |
| 1433543_at | Anln | anillin, actin binding protein (scraps homolog, Drosophila) | -2.4 | -15.6 | -6.6 |
| 1426243_at | Cth | cystathionase (cystathionine gamma-lyase) | -30.7 | -200.1 | -6.5 |
| 1416579_a_at | Tacstd1 | tumor-associated calcium signal transducer 1 | -1.6 | -10.5 | -6.5 |
| 1416757_at | Zwilch | Zwilch, kinetochore associated, homolog (Drosophila) | -4.4 | -28.2 | -6.4 |
| 1416961_at | Bub1b | budding uninhibited by benzimidazoles 1 homolog, beta (S. cerevisiae) | -2.8 | -18.1 | -6.4 |
| 1429294_at | Trip13 | thyroid hormone receptor interactor 13 | -2.0 | -13.0 | -6.4 |
| 1450842_a_at | Cenpa | centromere autoantigen A | -1.7 | -10.8 | -6.3 |
| 1450995_at | Folr1 | folate receptor 1 (adult) | -2.4 | -15.5 | -6.3 |
| 1417586_at | Timeless | timeless homolog (Drosophila) | -2.6 | -16.1 | -6.3 |
| 1423920_at | Brrn1 | barren homolog (Drosophila) | -1.8 | -11.4 | -6.3 |
| 1448226_at | Rrm2 | ribonucleotide reductase M2 | -2.3 | -14.3 | -6.3 |
| 1417963_at | Pltp | phospholipid transfer protein | -1.8 | -11.0 | -6.2 |
| 1456140_at | Zic5 | Opr | -1.7 | -10.6 | -6.2 |
| 1460726_at | Adss | adenylosuccinate synthetase, non muscle | -1.6 | -10.1 | -6.1 |
| 1418351_a_at | Dnmt3b | DNA methyltransferase 3B | -2.5 | -15.3 | -6.1 |
| 1430700_a_at | Pla2g7 | phospholipase A2, group VII (platelet-activating factor acetylhydrolase, plasma) | -4.4 | -26.7 | -6.1 |
| 1434528_at | Aard | alanine and arginine rich domain containing protein | -10.8 | -64.6 | -6.0 |
| 1448414_at | Rad1 | RAD1 homolog (S. pombe) | -3.0 | -18.2 | -6.0 |
| 1434767_at | C79407 | expressed sequence C79407 | -3.1 | -18.5 | -6.0 |
| 1433862_at | Espl1 | extra spindle poles-like 1 (S. cerevisiae) | -3.0 | -18.1 | -6.0 |
| 1448899_s_at | Rad51ap1 | RAD51 associated protein 1 | -1.8 | -10.6 | -5.9 |
| 1436174_at | Atad2 | ATPase family, AAA domain containing 2 | -2.4 | -14.2 | -5.9 |
| 1435005_at | Cenpe | centromere protein E | -1.8 | -10.4 | -5.9 |
| 1434911_s_at | Arhgap19 | Rho GTPase activating protein 19 | -1.7 | -9.9 | -5.9 |
| 1451526_at | Arhgap12 | Rho GTPase activating protein 12 | -1.8 | -10.7 | -5.8 |
| 1448635_at | Smc2l1 | SMC2 structural maintenance of chromosomes 2-like 1 (yeast) | -1.8 | -10.5 | -5.8 |
| 1418036_at | Prim2 | DNA primase, p58 subunit | -2.4 | -13.9 | -5.8 |
| 1448466_at | Cdca5 | cell division cycle associated 5 | -3.6 | -20.6 | -5.8 |
| 1437218_at | Fn1 | fibronectin 1 | -2.2 | -12.6 | -5.8 |
| 1460180_at | Hexb | hexosaminidase B | -2.4 | -13.7 | -5.7 |
| 1423156_at | Gnpnat1 | glucosamine-phosphate N-acetyltransferase 1 | -3.2 | -18.4 | -5.7 |
| 1452314_at | Kif11 | kinesin family member 11 | -2.9 | -16.2 | -5.7 |
| 1424105_a_at | Pttg1 | pituitary tumor-transforming 1 | -1.4 | -8.0 | -5.6 |
| 1417460_at | Ifitm2 | interferon induced transmembrane protein 2 | -1.7 | -9.8 | -5.6 |
| 1449060_at | Kif2c | kinesin family member 2C | -3.0 | -16.8 | -5.6 |
| 1426353_at | Stat6 | signal transducer and activator of transcription 6 | -2.6 | -14.9 | -5.6 |
| 1424107_at | Kif18a | kinesin family member 18A | -3.1 | -17.4 | -5.6 |
| 1416641_at | Lig1 | ligase I, DNA, ATP-dependent | -2.3 | -12.9 | -5.6 |
| 1455195_at | Rps24 | ribosomal protein S24 | -3.4 | -18.8 | -5.6 |
| 1448205_at | Ccnb1-rs1; Ccnb1 | cyclin B1, related sequence 1; cyclin B1 | -2.3 | -12.9 | -5.5 |
| 1450920_at | Ccnb2 | cyclin B2 | -2.5 | -13.8 | -5.5 |
| 1418369_at | Prim1 | DNA primase, p49 subunit | -2.5 | -13.6 | -5.4 |
| 1453283_at | Pgm1 | phosphoglucomutase 1 | -1.2 | -6.3 | -5.3 |
| 1417587_at | Timeless | timeless homolog (Drosophila) | -2.9 | -15.2 | -5.3 |
| 1451358_a_at | Racgap1 | Rac GTPase-activating protein 1 | -1.5 | -7.9 | -5.3 |
| 1422460_at | Mad2l1 | MAD2 (mitotic arrest deficient, homolog)-like 1 (yeast) | -1.7 | -9.0 | -5.3 |
| 1416258_at | Tk1 | thymidine kinase 1 | -1.6 | -8.4 | -5.3 |
| 1416664_at | Cdc20 | cell division cycle 20 homolog (S. cerevisiae) | -2.0 | -10.6 | -5.3 |
| 1447363_s_at | Bub1b | budding uninhibited by benzimidazoles 1 homolog, beta (S. cerevisiae) | -2.8 | -15.0 | -5.3 |
| 1424144_at | Ris2 | retroviral integration site 2 | -2.9 | -15.1 | -5.2 |
| 1450044_at | Fzd7 | frizzled homolog 7 (Drosophila) | -1.5 | -7.7 | -5.2 |
| 1427094_at | Pole2 | polymerase (DNA directed), epsilon 2 (p59 subunit) | -3.4 | -17.7 | -5.2 |
| 1423774_a_at | Prc1 | protein regulator of cytokinesis 1 | -1.7 | -8.8 | -5.1 |
| 1452534_a_at | Hmgb2 | high mobility group box 2 | -1.5 | -7.5 | -5.1 |
| 1435386_at | Vwf | Von Willebrand factor homolog | -2.7 | -13.8 | -5.1 |
| 1428481_s_at | Cdca8 | cell division cycle associated 8 | -1.3 | -6.6 | -5.0 |
| 1452458_s_at | Ppil5 | peptidylprolyl isomerase (cyclophilin) like 5 | -3.0 | -14.6 | -4.8 |
| 1417559_at | Sfxn1 | sideroflexin 1 | -1.2 | -5.9 | -4.8 |
| 1420425_at | Prdm1 | PR domain containing 1, with ZNF domain | -4.0 | -19.3 | -4.8 |
| 1450677_at | Chek1 | checkpoint kinase 1 homolog (S. pombe) | -3.5 | -16.6 | -4.8 |
| 1452583_s_at | Galm | galactose mutarotase | -2.2 | -10.3 | -4.8 |
| 1452004_at | Calca | calcitonin/calcitonin-related polypeptide, alpha | -10.8 | -51.2 | -4.7 |
| 1427302_at | Enpp3 | ectonucleotide pyrophosphatase/phosphodiesterase 3 | -20.6 | -97.1 | -4.7 |
| 1453600_at | Ccdc18 | coiled-coil domain containing 18 | -3.7 | -17.4 | -4.7 |
| 1423969_at | Nup37 | nucleoporin 37 | -2.4 | -11.2 | -4.7 |
| 1436723_at | Fshprh1 | FSH primary response 1 | -2.4 | -11.0 | -4.7 |
| 1434734_at | Rad54b | RAD54 homolog B (S. cerevisiae) | -3.8 | -17.6 | -4.7 |
| 1417392_a_at | Slc7a7 | solute carrier family 7 (cationic amino acid transporter, y+ system), member 7 | -4.3 | -20.3 | -4.7 |
| 1452226_at | Rcc2 | regulator of chromosome condensation 2 | -2.7 | -12.5 | -4.7 |
| 1416214_at | Mcm4 | minichromosome maintenance deficient 4 homolog (S. cerevisiae) | -1.7 | -8.1 | -4.6 |
| 1438434_at | Arhgap11a | Rho GTPase activating protein 11A | -1.7 | -7.6 | -4.6 |
| 1417823_at | Gcat | glycine C-acetyltransferase (2-amino-3-ketobutyrate-coenzyme A ligase) | -5.3 | -24.6 | -4.6 |
| 1415829_at | Lbr | lamin B receptor | -2.0 | -9.3 | -4.6 |
| 1434949_at | Armc8 | armadillo repeat containing 8 | -1.8 | -8.0 | -4.6 |
| 1434073_at | Gprasp2 | G protein-coupled receptor associated sorting protein 2 | -1.2 | -5.4 | -4.5 |
| 1435465_at | Kbtbd11 | kelch repeat and BTB (POZ) domain containing 11 | -8.3 | -37.5 | -4.5 |
| 1451461_a_at | Aldoc | aldolase 3, C isoform | -1.4 | -6.4 | -4.5 |
| 1421344_a_at | Jub | ajuba | -1.8 | -7.9 | -4.5 |
| 1456433_at | Rcbtb1 | regulator of chromosome condensation (RCC1) and BTB (POZ) domain containing protein 1 | -1.3 | -6.0 | -4.5 |
| 1426653_at | Mcm3 | minichromosome maintenance deficient 3 (S. cerevisiae) | -3.7 | -16.4 | -4.5 |
| 1426580_at | Plk4 | polo-like kinase 4 (Drosophila) | -1.8 | -8.2 | -4.5 |
| 1427707_a_at | Sil | Tal1 interrupting locus | -1.3 | -5.6 | -4.4 |
| 1418380_at | Terf1 | telomeric repeat binding factor 1 | -3.0 | -13.3 | -4.4 |
| 1450090_at | Zfp101 | zinc finger protein 101 | -1.4 | -6.3 | -4.4 |
| 1417022_at | Slc7a3 | solute carrier family 7 (cationic amino acid transporter, y+ system), member 3 | -15.2 | -66.4 | -4.4 |
| 1455007_s_at | Gpt2 | glutamic pyruvate transaminase (alanine aminotransferase) 2 | -5.0 | -21.8 | -4.3 |
| 1417910_at | Ccna2 | cyclin A2 | -1.7 | -7.5 | -4.3 |
| 1417445_at | Kntc2 | kinetochore associated 2 | -2.7 | -11.6 | -4.3 |
| 1424321_at | Rfc4 | replication factor C (activator 1) 4 | -1.9 | -8.1 | -4.3 |
| 1418530_at | Nup160 | nucleoporin 160 | -2.4 | -10.5 | -4.3 |
| 1417911_at | Ccna2 | cyclin A2 | -1.9 | -8.2 | -4.3 |
| 1417777_at | Ltb4dh | leukotriene B4 12-hydroxydehydrogenase | -1.2 | -5.2 | -4.3 |
| 1450886_at | Gsg2 | germ cell-specific gene 2 | -3.6 | -15.2 | -4.3 |
| 1425179_at | Shmt1 | serine hydroxymethyl transferase 1 (soluble) | -3.5 | -14.6 | -4.2 |
| 1423316_at | Tmem39a | transmembrane protein 39a | -2.4 | -10.3 | -4.2 |
| 1423123_at | Rad54l | RAD54 like (S. cerevisiae) | -3.3 | -13.8 | -4.2 |
| 1422946_a_at | Dnmt1 | DNA methyltransferase (cytosine-5) 1 | -1.9 | -7.9 | -4.2 |
| 1426649_at | Tmeff1 | transmembrane protein with EGF-like and two follistatin-like domains 1 | -1.8 | -7.5 | -4.2 |
| 1454731_at | Myo10 | myosin X | -3.7 | -15.4 | -4.2 |
| 1455990_at | Kif23 | kinesin family member 23 | -2.1 | -8.9 | -4.2 |
| 1415811_at | Uhrf1 | ubiquitin-like, containing PHD and RING finger domains, 1 | -3.7 | -15.4 | -4.1 |
| 1421924_at | Slc2a3 | solute carrier family 2 (facilitated glucose transporter), member 3 | -1.4 | -5.6 | -4.1 |
| 1429660_s_at | Smc2l1 | SMC2 structural maintenance of chromosomes 2-like 1 (yeast) | -2.4 | -9.8 | -4.1 |
| 1450692_at | Kif4 | kinesin family member 4 | -1.4 | -5.5 | -4.1 |
| 1425974_a_at | Trim25 | tripartite motif protein 25 | -2.4 | -9.5 | -4.0 |
| 1451717_s_at | Senp2 | SUMO/sentrin specific peptidase 2 | -2.2 | -9.0 | -4.0 |
| 1436808_x_at | Mcm5 | minichromosome maintenance deficient 5, cell division cycle 46 (S. cerevisiae) | -2.1 | -8.5 | -4.0 |
| 1417093_a_at | Gtf2h4 | general transcription factor II H, polypeptide 4 | -2.2 | -8.9 | -4.0 |
| 1425349_a_at | Myef2 | myelin basic protein expression factor 2, repressor | -1.3 | -5.1 | -4.0 |
| 1452050_at | Camk1d | calcium/calmodulin-dependent protein kinase ID | -4.8 | -18.9 | -4.0 |
| 1437213_at | Nudt21 | nudix (nucleoside diphosphate linked moiety X)-type motif 21 | -1.5 | -5.9 | -4.0 |
| 1426423_at | Shmt2 | serine hydroxymethyl transferase 2 (mitochondrial) | -5.0 | -19.7 | -4.0 |
| 1430811_a_at | Cdca1 | cell division cycle associated 1 | -1.5 | -5.7 | -3.9 |
| 1422482_at | Ruvbl2 | RuvB-like protein 2 | -1.9 | -7.4 | -3.9 |
| 1434719_at | A2m | alpha-2-macroglobulin | -2.4 | -9.3 | -3.9 |
| 1417883_at | Gstt2 | glutathione S-transferase, theta 2 | -2.9 | -11.1 | -3.9 |
| 1434282_at | Ibtk | inhibitor of Bruton agammaglobulinemia tyrosine kinase | -1.9 | -7.5 | -3.9 |
| 1455983_at | Cdca2 | cell division cycle associated 2 | -2.8 | -10.7 | -3.9 |
| 1417045_at | Bid | BH3 interacting domain death agonist | -3.3 | -12.6 | -3.8 |
| 1452459_at | Aspm | asp (abnormal spindle)-like, microcephaly associated (Drosophila) | -2.5 | -9.3 | -3.8 |
| 1455333_at | Tns3 | tensin 3 | -1.9 | -7.2 | -3.8 |
| 1423813_at | Kif22 | kinesin family member 22 | -3.2 | -12.1 | -3.8 |
| 1425006_a_at | Vrk1 | vaccinia related kinase 1 | -2.3 | -8.8 | -3.8 |
| 1419270_a_at | Dut | deoxyuridine triphosphatase | -1.4 | -5.2 | -3.8 |
| 1416730_at | Rcl1 | RNA terminal phosphate cyclase-like 1 | -2.3 | -8.5 | -3.8 |
| 1434437_x_at | Rrm2 | ribonucleotide reductase M2 | -2.0 | -7.5 | -3.8 |
| 1452210_at | Dna2l | DNA2 DNA replication helicase 2-like (yeast) | -2.2 | -8.2 | -3.8 |
| 1416031_s_at | Mcm7 | minichromosome maintenance deficient 7 (S. cerevisiae) | -2.7 | -10.1 | -3.7 |
| 1424511_at | Aurka | aurora kinase A | -2.2 | -8.0 | -3.7 |
| 1416988_at | Msh2 | mutS homolog 2 (E. coli) | -3.5 | -13.0 | -3.7 |
| 1416544_at | Ezh2 | enhancer of zeste homolog 2 (Drosophila) | -1.5 | -5.5 | -3.6 |
| 1416746_at | H2afx | H2A histone family, member X | -1.9 | -7.0 | -3.6 |
| 1425844_a_at | Rngtt | RNA guanylyltransferase and 5'-phosphatase | -2.0 | -7.1 | -3.6 |
| 1435114_at | Wdhd1 | WD repeat and HMG-box DNA binding protein 1 | -1.9 | -6.8 | -3.6 |
| 1434630_at | Ankrd28 | ankyrin repeat domain 28 | -1.4 | -5.2 | -3.6 |
| 1450743_s_at | Syncrip | synaptotagmin binding, cytoplasmic RNA interacting protein | -1.5 | -5.5 | -3.6 |
| 1417027_at | Trim2 | tripartite motif protein 2 | -4.5 | -16.3 | -3.6 |
| 1451095_at | Asns | asparagine synthetase | -4.2 | -15.1 | -3.6 |
| 1423543_at | MGI:1298390 | SWA-70 protein | -1.5 | -5.5 | -3.6 |
| 1452917_at | Rfc5 | replication factor C (activator 1) 5 | -4.6 | -16.5 | -3.6 |
| 1422286_a_at | Tgif | TG interacting factor | -4.3 | -15.3 | -3.5 |
| 1460229_at | Stag3 | stromal antigen 3 | -6.7 | -23.6 | -3.5 |
| 1454891_at | Cds2 | CDP-diacylglycerol synthase (phosphatidate cytidylyltransferase) 2 | -4.4 | -15.4 | -3.5 |
| 1450070_s_at | Pak1 | p21 (CDKN1A)-activated kinase 1 | -1.9 | -6.6 | -3.5 |
| 1430617_at | Oip5 | Opa interacting protein 5 | -2.4 | -8.5 | -3.5 |
| 1424156_at | Rbl1 | retinoblastoma-like 1 (p107) | -1.4 | -5.0 | -3.5 |
| 1428859_at | Paox | polyamine oxidase (exo-N4-amino) | -1.5 | -5.1 | -3.5 |
| 1423877_at | Chaf1b | chromatin assembly factor 1, subunit B (p60) | -2.2 | -7.7 | -3.5 |
| 1460253_at | Cklfsf7 | chemokine-like factor super family 7 | -2.3 | -8.1 | -3.5 |
| 1456653_a_at | Mthfd1l | methylenetetrahydrofolate dehydrogenase (NADP+ dependent) 1-like | -2.2 | -7.6 | -3.5 |
| 1460419_a_at | Prkcb1 | protein kinase C, beta 1 | -1.6 | -5.5 | -3.5 |
| 1417560_at | Sfxn1 | sideroflexin 1 | -1.2 | -4.0 | -3.4 |
| 1455277_at | Hhip | Hedgehog-interacting protein | -1.1 | -3.8 | -3.4 |
| 1421260_a_at | Srm | spermidine synthase | -2.2 | -7.4 | -3.4 |
| 1434554_at | Trim37 | tripartite motif protein 37 | -1.7 | -5.9 | -3.4 |
| 1416411_at | Gstm2 | glutathione S-transferase, mu 2 | -2.0 | -6.9 | -3.4 |
| 1450887_at | Rqcd1 | rcd1 (required for cell differentiation) homolog 1 (S. pombe) | -1.7 | -5.7 | -3.4 |
| 1428228_at | Pgm3 | phosphoglucomutase 3 | -1.9 | -6.6 | -3.4 |
| 1435737_a_at | Nde1 | nuclear distribution gene E homolog 1 (A nidulans) | -2.2 | -7.5 | -3.4 |
| 1427240_at | Dock6 | dedicator of cytokinesis 6 | -2.4 | -8.2 | -3.4 |
| 1416915_at | Msh6 | mutS homolog 6 (E. coli) | -4.6 | -15.7 | -3.4 |
| 1448720_at | Lrrc40 | leucine rich repeat containing 40 | -1.3 | -4.4 | -3.4 |
| 1452237_at | Hrb | HIV-1 Rev binding protein | -1.8 | -5.9 | -3.4 |
| 1417506_at | Gmnn | geminin | -2.2 | -7.5 | -3.4 |
| 1435113_x_at | Stmn3 | stathmin-like 3 | -5.0 | -16.5 | -3.3 |
| 1426712_at | Slc6a15 | solute carrier family 6 (neurotransmitter transporter), member 15 | -3.8 | -12.6 | -3.3 |
| 1416237_at | Eva1 | epithelial V-like antigen 1 | -1.6 | -5.3 | -3.3 |
| 1417297_at | Itpr3 | inositol 1,4,5-triphosphate receptor 3 | -1.6 | -5.3 | -3.3 |
| 1428949_at | Xpot | exportin, tRNA (nuclear export receptor for tRNAs) | -1.5 | -4.9 | -3.3 |
| 1452241_at | Topbp1 | topoisomerase (DNA) II beta binding protein | -1.9 | -6.2 | -3.3 |
| 1416301_a_at | Ebf1 | early B-cell factor 1 | -3.3 | -10.8 | -3.3 |
| 1426612_at | MGI:1921571 | timeless interacting protein | -2.4 | -7.6 | -3.2 |
| 1417404_at | Elovl6 | ELOVL family member 6, elongation of long chain fatty acids (yeast) | -3.1 | -10.2 | -3.2 |
| 1426897_at | Rcc2 | regulator of chromosome condensation 2 | -2.9 | -9.4 | -3.2 |
| 1436266_x_at | Cbx1 | chromobox homolog 1 (Drosophila HP1 beta) | -1.9 | -6.2 | -3.2 |
| 1451722_s_at | Smyd5 | SET and MYND domain containing 5 | -2.0 | -6.4 | -3.2 |
| 1451672_at | Gprk6 | G protein-coupled receptor kinase 6 | -1.8 | -5.8 | -3.2 |
| 1452384_at | Enpp3 | ectonucleotide pyrophosphatase/phosphodiesterase 3 | -14.7 | -46.8 | -3.2 |
| 1455832_a_at | Umps | uridine monophosphate synthetase | -2.4 | -7.6 | -3.2 |
| 1451080_at | Usp1 | ubiquitin specific peptdiase 1 | -2.2 | -7.0 | -3.2 |
| 1452115_a_at | Plk4 | polo-like kinase 4 (Drosophila) | -1.9 | -6.0 | -3.2 |
| 1433892_at | Spag5 | sperm associated antigen 5 | -2.6 | -8.1 | -3.2 |
| 1417352_s_at | Snrpa1 | small nuclear ribonucleoprotein polypeptide A' | -1.4 | -4.3 | -3.2 |
| 1418656_at | Lsm5 | LSM5 homolog, U6 small nuclear RNA associated (S. cerevisiae) | -1.6 | -5.1 | -3.1 |
| 1415888_at | Hdgf | hepatoma-derived growth factor | -1.6 | -4.9 | -3.1 |
| 1430574_at | Cdkn3 | cyclin-dependent kinase inhibitor 3 | -1.4 | -4.2 | -3.1 |
| 1415917_at | Mthfd1 | methylenetetrahydrofolate dehydrogenase (NADP+ dependent), methenyltetrahydrofolate cyclohydrolase, formyltetrahydrofolate synthase | -3.6 | -11.1 | -3.1 |
| 1451374_x_at | Cklf | chemokine-like factor | -1.3 | -3.9 | -3.1 |
| 1428326_s_at | Hrsp12 | heat-responsive protein 12 | -1.3 | -3.9 | -3.1 |
| 1435275_at | Cox6b2 | cytochrome c oxidase subunit VIb polypeptide 2 | -2.0 | -6.1 | -3.1 |
| 1429557_at | Mcm8 | minichromosome maintenance deficient 8 (S. cerevisiae) | -3.1 | -9.5 | -3.0 |
| 1425166_at | Rbl1 | retinoblastoma-like 1 (p107) | -1.4 | -4.4 | -3.0 |
| 1455966_s_at | Nudt21 | nudix (nucleoside diphosphate linked moiety X)-type motif 21 | -1.4 | -4.3 | -3.0 |
| 1449853_at | Sfxn2 | sideroflexin 2 | -1.7 | -5.3 | -3.0 |
| 1418086_at | Ppp1r14a | protein phosphatase 1, regulatory (inhibitor) subunit 14A | -1.6 | -4.7 | -3.0 |
| 1423078_a_at | Sc4mol | sterol-C4-methyl oxidase-like | -1.9 | -5.7 | -3.0 |
| 1431087_at | Spbc24 | spindle pole body component 24 homolog (S. cerevisiae) | -2.9 | -8.8 | -3.0 |
| 1415773_at | Ncl | nucleolin | -2.0 | -6.1 | -3.0 |
| 1417948_s_at | Ilf2 | interleukin enhancer binding factor 2 | -1.8 | -5.4 | -3.0 |
| 1441317_x_at | MGI:1923321 | gamma-aminobutyric acid (GABA-B) receptor binding protein | -10.7 | -31.9 | -3.0 |
| 1457027_at | Dhtkd1 | dehydrogenase E1 and transketolase domain containing 1 | -4.1 | -12.3 | -3.0 |
| 1453722_s_at | Sfrs1 | splicing factor, arginine/serine-rich 1 (ASF/SF2) | -1.2 | -3.7 | -3.0 |
| 1417166_at | Psip1 | PC4 and SFRS1 interacting protein 1 | -2.1 | -6.1 | -3.0 |
| 1417167_at | Exosc5 | exosome component 5 | -4.5 | -13.4 | -3.0 |
| 1448187_at | Pold1 | polymerase (DNA directed), delta 1, catalytic subunit | -3.5 | -10.3 | -3.0 |
| 1423848_at | Mphosph6 | M phase phosphoprotein 6 (Mphosph6), mRNA | -1.3 | -3.9 | -2.9 |
| 1426555_at | Scpep1 | serine carboxypeptidase 1 | -1.8 | -5.3 | -2.9 |
| 1436056_at | Kif13b | kinesin family member 13B | -2.1 | -6.3 | -2.9 |
| 1422622_at | Nos3 | nitric oxide synthase 3, endothelial cell | -1.3 | -3.9 | -2.9 |
| 1440924_at | Mphosph1 | M-phase phosphoprotein 1 | -3.6 | -10.6 | -2.9 |
| 1428100_at | Sfrs1 | splicing factor, arginine/serine-rich 1 (ASF/SF2) | -1.8 | -5.2 | -2.9 |
| 1450417_a_at | Rps20 | ribosomal protein S20 | -2.3 | -6.8 | -2.9 |
| 1418761_at | Igf2bp1 | insulin-like growth factor 2, binding protein 1 | -1.6 | -4.6 | -2.9 |
| 1418217_at | Nme7 | non-metastatic cells 7, protein expressed in | -3.0 | -8.7 | -2.9 |
| 1417353_x_at | Snrpa1 | small nuclear ribonucleoprotein polypeptide A' | -1.4 | -3.9 | -2.9 |
| 1422612_at | Hk2 | hexokinase 2 | -1.7 | -4.9 | -2.9 |
| 1426946_at | Ranbp5 | RAN binding protein 5 | -1.6 | -4.6 | -2.9 |
| 1454846_at | Utp15 | UTP15, U3 small nucleolar ribonucleoprotein, homolog (yeast) | -1.5 | -4.3 | -2.9 |
| 1451884_a_at | Lsm2 | LSM2 homolog, U6 small nuclear RNA associated (S. cerevisiae) | -1.9 | -5.4 | -2.9 |
| 1452787_a_at | Hrmt1l2 | heterogeneous nuclear ribonucleoproteins methyltransferase-like 2 (S. cerevisiae) | -1.3 | -3.6 | -2.9 |
| 1423361_at | Yme1l1 | YME1-like 1 (S. cerevisiae) | -1.3 | -3.8 | -2.9 |
| 1419741_at | Supt16h | suppressor of Ty 16 homolog (S. cerevisiae) | -1.6 | -4.5 | -2.8 |
| 1422252_a_at | Cdc25c | cell division cycle 25 homolog C (S. cerevisiae) | -1.5 | -4.3 | -2.8 |
| 1441520_at | Aspm | asp (abnormal spindle)-like, microcephaly associated (Drosophila) | -3.2 | -9.1 | -2.8 |
| 1451968_at | Xrcc5 | X-ray repair complementing defective repair in Chinese hamster cells 5 | -5.0 | -14.1 | -2.8 |
| 1451199_at | Qtrtd1 | queuine tRNA-ribosyltransferase domain containing 1 | -1.9 | -5.4 | -2.8 |
| 1416184_s_at | Hmga1 | high mobility group AT-hook 1 | -1.6 | -4.6 | -2.8 |
| 1436851_at | Pkn1 | protein kinase N1 | -1.8 | -5.1 | -2.8 |
| 1416897_at | Parp9 | poly (ADP-ribose) polymerase family, member 9 | -1.3 | -3.6 | -2.8 |
| 1423819_s_at | Arl6ip1 | ADP-ribosylation factor-like 6 interacting protein 1 | -1.2 | -3.3 | -2.8 |
| 1453290_at | Hmgb2l1 | high mobility group box 2-like 1 | -1.5 | -4.1 | -2.8 |
| 1426447_at | Nup35 | nucleoporin 35 | -2.5 | -7.2 | -2.8 |
| 1416575_at | Cdc45l | cell division cycle 45 homolog (S. cerevisiae)-like | -2.6 | -7.4 | -2.8 |
| 1454743_at | Nup205 | nucleoporin 205 | -1.9 | -5.2 | -2.8 |
| 1449278_at | Eif2ak3 | eukaryotic translation initiation factor 2 alpha kinase 3 | -1.3 | -3.5 | -2.8 |
| 1416748_a_at | Mre11a | meiotic recombination 11 homolog A (S. cerevisiae) | -1.7 | -4.6 | -2.8 |
| 1436222_at | Gas5 | growth arrest specific 5 | -1.7 | -4.7 | -2.8 |
| 1460168_at | Slbp | stem-loop binding protein | -1.2 | -3.2 | -2.8 |
| 1428374_at | Glce | glucuronyl C5-epimerase | -1.4 | -4.0 | -2.8 |
| 1417057_a_at | Ppid; Lamp3 | peptidylprolyl isomerase D (cyclophilin D); lysosomal-associated membrane protein 3 | -1.2 | -3.4 | -2.8 |
| 1425831_at | Zfp101 | zinc finger protein 101 | -1.1 | -3.0 | -2.8 |
| 1435753_a_at | Nucks1 | nuclear casein kinase and cyclin-dependent kinase substrate 1 | -2.3 | -6.3 | -2.8 |
| 1448646_at | Wdr12 | WD repeat domain 12 | -1.9 | -5.1 | -2.7 |
| 1415836_at | Aldh18a1 | aldehyde dehydrogenase 18 family, member A1 | -2.1 | -5.9 | -2.7 |
| 1415860_at | Kpna2 | karyopherin (importin) alpha 2 | -1.7 | -4.5 | -2.7 |
| 1423700_at | Rfc3 | replication factor C (activator 1) 3 | -1.5 | -4.2 | -2.7 |
| 1449490_at | Mbd4 | methyl-CpG binding domain protein 4 | -1.7 | -4.6 | -2.7 |
| 1425248_a_at | Tyro3 | TYRO3 protein tyrosine kinase 3 | -3.0 | -8.1 | -2.7 |
| 1426945_at | Ranbp5 | RAN binding protein 5 | -1.5 | -4.1 | -2.7 |
| 1434000_at | Kras | v-Ki-ras2 Kirsten rat sarcoma viral oncogene homolog | -1.3 | -3.4 | -2.7 |
| 1448330_at | Gstm1 | glutathione S-transferase, mu 1 | -1.7 | -4.7 | -2.7 |
| 1418027_at | Exo1 | exonuclease 1 | -2.9 | -7.9 | -2.7 |
| 1419253_at | Mthfd2 | methylenetetrahydrofolate dehydrogenase (NAD+ dependent), methenyltetrahydrofolate cyclohydrolase | -3.4 | -9.2 | -2.7 |
| 1427213_at | Pfkfb1 | 6-phosphofructo-2-kinase/fructose-2,6-biphosphatase 1 | -1.4 | -3.7 | -2.7 |
| 1429399_at | Rnf125 | ring finger protein 125 | -7.9 | -21.3 | -2.7 |
| 1415993_at | Sqle | squalene epoxidase | -1.5 | -3.9 | -2.7 |
| 1425271_at | Psmc3ip | proteasome (prosome, macropain) 26S subunit, ATPase 3, interacting protein | -1.7 | -4.5 | -2.7 |
| 1426953_at | Hmgb2l1 | high mobility group box 2-like 1 | -2.6 | -7.0 | -2.7 |
| 1426838_at | Pold3 | polymerase (DNA-directed), delta 3, accessory subunit | -2.5 | -6.7 | -2.7 |
| 1431506_s_at | Ppih | peptidyl prolyl isomerase H | -2.1 | -5.7 | -2.7 |
| 1421151_a_at | Epha2 | Eph receptor A2 | -3.7 | -9.9 | -2.7 |
| 1425548_a_at | Lst1 | leukocyte specific transcript 1 | -1.6 | -4.3 | -2.7 |
| 1424300_at | Gemin6 | gem (nuclear organelle) associated protein 6 | -2.4 | -6.3 | -2.7 |
| 1431502_a_at | Exosc3 | exosome component 3 | -2.2 | -5.9 | -2.7 |
| 1423525_at | Mastl | microtubule associated serine/threonine kinase-like | -2.4 | -6.3 | -2.7 |
| 1419402_at | Mns1 | meiosis-specific nuclear structural protein 1 | -1.2 | -3.1 | -2.7 |
| 1419722_at | Prss19 | protease, serine, 19 (neuropsin) | -3.0 | -7.9 | -2.6 |
| 1421935_at | Rps20 | ribosomal protein S20 | -2.3 | -6.2 | -2.6 |
| 1450866_a_at | Mrpl17 | mitochondrial ribosomal protein L17 | -1.2 | -3.2 | -2.6 |
| 1433622_at | Gemin4 | gem (nuclear organelle) associated protein 4 | -2.3 | -6.1 | -2.6 |
| 1426572_at | Me2 | malic enzyme 2, NAD(+)-dependent, mitochondrial | -1.3 | -3.5 | -2.6 |
| 1455109_at | Tbl1xr1 | transducin (beta)-like 1X-linked receptor 1 | -1.6 | -4.3 | -2.6 |
| 1422663_at | Orc1l | origin recognition complex, subunit 1-like (S.cereviaiae) | -4.5 | -11.9 | -2.6 |
| 1439788_at | MGC86034 | hypothetical MGC86034 | -2.3 | -6.0 | -2.6 |
| 1449008_at | Tulp3 | tubby-like protein 3 | -1.7 | -4.4 | -2.6 |
| 1418160_at | Mkrn3 | makorin, ring finger protein, 3 | -1.2 | -3.0 | -2.6 |
| 1427305_at | Piga | phosphatidylinositol glycan, class A | -1.7 | -4.4 | -2.6 |
| 1452830_s_at | Cad | carbamoyl-phosphate synthetase 2, aspartate transcarbamylase, and dihydroorotase | -2.0 | -5.2 | -2.6 |
| 1422462_at | Ube2t | ubiquitin-conjugating enzyme E2T (putative) | -3.2 | -8.4 | -2.6 |
| 1433536_at | Lrp11 | low density lipoprotein receptor-related protein 11 | -2.8 | -7.2 | -2.6 |
| 1432188_s_at | Nup43 | nucleoporin 43 | -3.0 | -7.7 | -2.6 |
| 1448938_at | Rpa3 | replication protein A3 | -1.9 | -4.9 | -2.6 |
| 1425396_a_at | Lck | lymphocyte protein tyrosine kinase | -2.2 | -5.8 | -2.6 |
| 1434225_at | MGI:1298390 | SWA-70 protein | -1.4 | -3.6 | -2.6 |
| 1417785_at | Pla1a | phospholipase A1 member A | -6.9 | -17.8 | -2.6 |
| 1438076_at | Rpl30 | ribosomal protein L30 | -1.7 | -4.4 | -2.6 |
| 1439966_x_at | Sfxn2 | Sideroflexin 2 | -1.7 | -4.4 | -2.6 |
| 1435054_at | Eme1 | essential meiotic endonuclease 1 homolog 1 (S. pombe) | -2.7 | -6.8 | -2.6 |
| 1416449_x_at | Stxbp2 | syntaxin binding protein 2 | -1.1 | -2.9 | -2.6 |
| 1443750_s_at | Rpp40 | ribonuclease P 40 subunit (human) | -3.0 | -7.7 | -2.6 |
| 1417724_at | Thoc4 | THO complex 4 | -1.7 | -4.4 | -2.6 |
| 1437658_a_at | Rnu22 | RNA, U22 small nucleolar | -2.0 | -5.0 | -2.5 |
| 1416045_a_at | Smarcb1 | SWI/SNF related, matrix associated, actin dependent regulator of chromatin, subfamily b, member 1 | -1.4 | -3.7 | -2.5 |
| 1438516_at | Rif1 | Rap1-interacting factor 1 (Rif1) | -3.8 | -9.7 | -2.5 |
| 1426538_a_at | Trp53 | transformation related protein 53 | -2.8 | -7.0 | -2.5 |
| 1433718_a_at | Cbx1 | chromobox homolog 1 (Drosophila HP1 beta) | -2.0 | -5.0 | -2.5 |
| 1439007_at | Alg6 | asparagine-linked glycosylation 6 homolog (yeast, alpha-1,3,-glucosyltransferase) | -1.4 | -3.5 | -2.5 |
| 1437108_at | Lsm6 | LSM6 homolog, U6 small nuclear RNA associated (S. cerevisiae) | -1.2 | -2.9 | -2.5 |
| 1424033_at | Sfrs7 | splicing factor, arginine/serine-rich 7 | -1.4 | -3.6 | -2.5 |
| 1423796_at | Sfpq | splicing factor proline/glutamine rich (polypyrimidine tract binding protein associated) | -1.4 | -3.5 | -2.5 |
| 1454142_a_at | Pwp1 | PWP1 homolog (S. cerevisiae) | -1.3 | -3.2 | -2.5 |
| 1452829_at | Cad | carbamoyl-phosphate synthetase 2, aspartate transcarbamylase, and dihydroorotase | -2.0 | -4.9 | -2.5 |
| 1418326_at | Slc7a5 | solute carrier family 7 (cationic amino acid transporter, y+ system), member 5 | -4.1 | -10.4 | -2.5 |
| 1416416_x_at | Gstm1 | glutathione S-transferase, mu 1 | -1.5 | -3.9 | -2.5 |
| 1448354_at | G6pdx | glucose-6-phosphate dehydrogenase X-linked | -1.3 | -3.3 | -2.5 |
| 1423524_at | Mastl | microtubule associated serine/threonine kinase-like | -2.3 | -5.9 | -2.5 |
| 1423675_at | Usp1 | ubiquitin specific peptdiase 1 | -2.6 | -6.5 | -2.5 |
| 1441890_x_at | Tmeff1 | transmembrane protein with EGF-like and two follistatin-like domains 1 | -2.0 | -5.0 | -2.5 |
| 1448638_at | Mtbp | Mdm2, transformed 3T3 cell double minute p53 binding protein | -2.7 | -6.6 | -2.5 |
| 1415849_s_at | Stmn1 | stathmin 1 | -1.5 | -3.7 | -2.5 |
| 1417132_at | Cdc25a | cell division cycle 25 homolog A (S. cerevisiae) | -1.4 | -3.6 | -2.5 |
| 1452197_at | Smc4l1 | SMC4 structural maintenance of chromosomes 4-like 1 (yeast) | -1.5 | -3.8 | -2.5 |
| 1455704_at | Xkr8 | X Kell blood group precursor related family member 8 homolog | -1.1 | -2.6 | -2.5 |
| 1417832_at | Smc1l1 | SMC (structural maintenance of chromosomes 1)-like 1 (S. cerevisiae) | -1.6 | -3.9 | -2.5 |
| 1424207_at | Smarca5 | SWI/SNF related, matrix associated, actin dependent regulator of chromatin, subfamily a, member 5 | -1.4 | -3.4 | -2.5 |
| 1449346_s_at | Riok1 | RIO kinase 1 (yeast) | -1.6 | -3.9 | -2.4 |
| 1452098_at | Chtf18 | CTF18, chromosome transmission fidelity factor 18 homolog (S. cerevisiae) | -5.4 | -13.2 | -2.4 |
| 1433547_s_at | Nudcd1 | NudC domain containing 1 | -1.4 | -3.5 | -2.4 |
| 1416152_a_at | Sfrs3 | splicing factor, arginine/serine-rich 3 (SRp20) | -1.7 | -4.1 | -2.4 |
| 1423703_at | Ppan | peter pan homolog (Drosophila) | -2.7 | -6.6 | -2.4 |
| 1421430_at | Rad51l1 | RAD51-like 1 (S. cerevisiae) | -1.6 | -3.8 | -2.4 |
| 1425538_x_at | Ceacam1 | CEA-related cell adhesion molecule 1 | -5.5 | -13.4 | -2.4 |
| 1422547_at | Ranbp1 | RAN binding protein 1 | -1.6 | -3.9 | -2.4 |
| 1426257_a_at | Sars1 | seryl-aminoacyl-tRNA synthetase 1 | -1.8 | -4.4 | -2.4 |
| 1418828_at | Thex1 | three prime histone mRNA exonuclease 1 | -1.7 | -4.2 | -2.4 |
| 1436171_at | Arhgap30 | Rho GTPase activating protein 30 | -1.2 | -2.9 | -2.4 |
| 1427058_at | Eif4a1 | eukaryotic translation initiation factor 4A1 | -1.6 | -3.9 | -2.4 |
| 1419086_at | Fgfbp1 | fibroblast growth factor binding protein 1 | -1.9 | -4.6 | -2.4 |
| 1446086_s_at | Gli2 | GLI-Kruppel family member GLI2 | -2.9 | -6.8 | -2.4 |
| 1450650_at | Myo10 | myosin X | -3.2 | -7.7 | -2.4 |
| 1448953_at | Blm | Bloom syndrome homolog (human) | -2.7 | -6.5 | -2.4 |
| 1437436_s_at | Gprk6 | G protein-coupled receptor kinase 6 | -1.9 | -4.4 | -2.4 |
| 1417094_at | Acot7 | acyl-CoA thioesterase 7 | -1.3 | -3.1 | -2.4 |
| 1416042_s_at | Nasp | nuclear autoantigenic sperm protein (histone-binding) | -2.3 | -5.4 | -2.4 |
| 1448130_at | Fdft1 | farnesyl diphosphate farnesyl transferase 1 | -1.9 | -4.4 | -2.4 |
| 1423787_at | Nup133 | nucleoporin 133 | -2.1 | -4.9 | -2.4 |
| 1450853_at | Tle4 | transducin-like enhancer of split 4, homolog of Drosophila E(spl) | -3.7 | -8.8 | -2.4 |
| 1423369_at | Fmr1 | fragile X mental retardation syndrome 1 homolog | -1.2 | -2.7 | -2.4 |
| 1439394_x_at | Cdc20 | cell division cycle 20 homolog (S. cerevisiae) | -2.2 | -5.1 | -2.4 |
| 1448234_at | Dnajb6 | DnaJ (Hsp40) homolog, subfamily B, member 6 | -1.1 | -2.6 | -2.4 |
| 1438385_s_at | Gpt2 | glutamic pyruvate transaminase (alanine aminotransferase) 2 | -4.0 | -9.4 | -2.4 |
| 1418736_at | B3galt3 | UDP-Gal:betaGlcNAc beta 1,3-galactosyltransferase, polypeptide 3 | -1.4 | -3.2 | -2.3 |
| 1427739_a_at | Trp53 | transformation related protein 53 | -2.9 | -6.8 | -2.3 |
| 1417681_at | Nudt21 | nudix (nucleoside diphosphate linked moiety X)-type motif 21 | -1.2 | -2.8 | -2.3 |
| 1451114_at | Cklfsf6 | chemokine-like factor super family 6 | -1.7 | -3.9 | -2.3 |
| 1452246_at | Ostf1 | osteoclast stimulating factor 1 | -1.2 | -2.8 | -2.3 |
| 1424569_at | Ddx46 | DEAD (Asp-Glu-Ala-Asp) box polypeptide 46 | -1.4 | -3.3 | -2.3 |
| 1454993_a_at | Sfrs3 | splicing factor, arginine/serine-rich 3 (SRp20) | -1.4 | -3.2 | -2.3 |
| 1428629_at | Zfp518 | zinc finger protein 518 | -2.1 | -4.8 | -2.3 |
| 1435781_at | Cand1 | cullin associated and neddylation disassociated 1 | -1.3 | -3.0 | -2.3 |
| 1422532_at | Xpc | xeroderma pigmentosum, complementation group C | -1.1 | -2.6 | -2.3 |
| 1429655_at | Nudcd1 | NudC domain containing 1 | -1.5 | -3.4 | -2.3 |
| 1420636_a_at | Dusp12 | dual specificity phosphatase 12 | -1.3 | -3.0 | -2.3 |
| 1441997_at | Zfp184 | zinc finger protein 184 (Kruppel-like) | -1.3 | -2.9 | -2.3 |
| 1416280_at | Uble1b | ubiquitin-like 1 (sentrin) activating enzyme E1B | -2.0 | -4.5 | -2.3 |
| 1439155_at | Mettl1 | methyltransferase-like 1 | -1.3 | -3.1 | -2.3 |
| 1435669_at | Zfp532 | zinc finger protein 532 | -1.7 | -4.0 | -2.3 |
| 1436227_at | Lefty2 | Left-right determination factor 2 | -31.9 | -72.8 | -2.3 |
| 1438339_at | Fancd2 | Fanconi anemia, complementation group D2 | -3.5 | -8.0 | -2.3 |
| 1422842_at | Xrn2 | 5'-3' exoribonuclease 2 | -1.9 | -4.3 | -2.3 |
| 1415978_at | Tubb3 | tubulin, beta 3 | -3.0 | -6.8 | -2.3 |
| 1452394_at | Cars | cysteinyl-tRNA synthetase | -4.8 | -10.9 | -2.3 |
| 1452811_at | Atic | 5-aminoimidazole-4-carboxamide ribonucleotide formyltransferase/IMP cyclohydrolase | -2.6 | -5.9 | -2.3 |
| 1418836_at | Qprt | quinolinate phosphoribosyltransferase | -3.7 | -8.4 | -2.3 |
| 1452000_s_at | Sars1 | seryl-aminoacyl-tRNA synthetase 1 | -1.9 | -4.4 | -2.3 |
| 1441910_x_at | Ccne1 | cyclin E1 | -3.4 | -7.7 | -2.3 |
| 1420401_a_at | Ramp3 | receptor (calcitonin) activity modifying protein 3 | -1.2 | -2.8 | -2.3 |
| 1428772_at | Xpot | exportin, tRNA (nuclear export receptor for tRNAs) | -2.2 | -4.9 | -2.3 |
| 1434079_s_at | Mcm2 | minichromosome maintenance deficient 2 mitotin (S. cerevisiae) | -2.3 | -5.2 | -2.2 |
| 1416530_a_at | Pnp | purine-nucleoside phosphorylase | -1.8 | -4.0 | -2.2 |
| 1423852_at | Tmem46 | transmembrane protein 46 | -2.6 | -5.9 | -2.2 |
| 1451516_at | Rhebl1 | Ras homolog enriched in brain like 1 | -3.0 | -6.7 | -2.2 |
| 1435618_at | Pnma2 | paraneoplastic antigen MA2 | -4.7 | -10.6 | -2.2 |
| 1448289_at | Crmp1 | collapsin response mediator protein 1 | -1.8 | -4.0 | -2.2 |
| 1456652_at | Dtl | denticleless homolog (Drosophila) | -1.8 | -4.0 | -2.2 |
| 1418394_a_at | Cd97 | CD97 antigen | -2.7 | -5.9 | -2.2 |
| 1422433_s_at | Idh1 | isocitrate dehydrogenase 1 (NADP+), soluble | -1.2 | -2.6 | -2.2 |
| 1435630_s_at | Acat2 | acetyl-Coenzyme A acetyltransferase 2 | -1.5 | -3.3 | -2.2 |
| 1436802_at | Ilf3 | interleukin enhancer binding factor 3 | -1.7 | -3.7 | -2.2 |
| 1454852_at | Sp1 | trans-acting transcription factor 1 | -1.5 | -3.2 | -2.2 |
| 1421287_a_at | Pecam1 | platelet/endothelial cell adhesion molecule 1 | -1.2 | -2.6 | -2.2 |
| 1417401_at | Rai14 | retinoic acid induced 14 | -1.4 | -3.0 | -2.2 |
| 1417983_a_at | Ube2v2 | ubiquitin-conjugating enzyme E2 variant 2 | -1.1 | -2.4 | -2.2 |
| 1456748_a_at | Nipsnap1 | 4-nitrophenylphosphatase domain and non-neuronal SNAP25-like protein homolog 1 (C. elegans) | -3.0 | -6.5 | -2.2 |
| 1427630_x_at | Ceacam1 | CEA-related cell adhesion molecule 1 | -4.5 | -9.8 | -2.2 |
| 1423964_at | Cpsf3l | cleavage and polyadenylation specific factor 3-like | -1.8 | -3.9 | -2.2 |
| 1420919_at | Sgk3 | serum/glucocorticoid regulated kinase 3 | -1.8 | -4.0 | -2.2 |
| 1417939_at | Rad51ap1 | RAD51 associated protein 1 | -1.8 | -3.9 | -2.2 |
| 1426378_at | Eif4b | eukaryotic translation initiation factor 4B | -1.2 | -2.6 | -2.2 |
| 1416715_at | Gjb3 | gap junction membrane channel protein beta 3 | -9.7 | -21.1 | -2.2 |
| 1449115_at | Mtf2 | metal response element binding transcription factor 2 | -5.1 | -11.1 | -2.2 |
| 1417548_at | Sart3 | squamous cell carcinoma antigen recognized by T-cells 3 | -2.0 | -4.3 | -2.2 |
| 1448652_at | Ttc10 | tetratricopeptide repeat domain 10 | -1.5 | -3.3 | -2.2 |
| 1451163_at | Tinf2 | Terf1 (TRF1)-interacting nuclear factor 2 | -1.9 | -4.1 | -2.2 |
| 1415765_at | Hnrpul2 | heterogeneous nuclear ribonucleoprotein U-like 2 | -1.5 | -3.2 | -2.2 |
| 1448369_at | Pola2 | polymerase (DNA directed), alpha 2 | -2.4 | -5.2 | -2.2 |
| 1448633_at | Prpf31 | PRP31 pre-mRNA processing factor 31 homolog (yeast) | -1.4 | -3.1 | -2.2 |
| 1443954_at | Rad18 | RAD18 homolog (S. cerevisiae) | -2.5 | -5.4 | -2.2 |
| 1416586_at | Zfp239 | zinc finger protein 239 | -2.0 | -4.3 | -2.2 |
| 1454064_a_at | Rnf138 | ring finger protein 138 | -2.9 | -6.1 | -2.1 |
| 1428291_at | Exosc8 | exosome component 8 | -1.9 | -4.1 | -2.1 |
| 1449044_at | Eef1e1 | eukaryotic translation elongation factor 1 epsilon 1 | -2.3 | -4.9 | -2.1 |
| 1416461_at | Gpiap1 | GPI-anchored membrane protein 1 | -1.3 | -2.8 | -2.1 |
| 1416968_a_at | Hsd3b7 | hydroxy-delta-5-steroid dehydrogenase, 3 beta- and steroid delta-isomerase 7 | -1.1 | -2.4 | -2.1 |
| 1422021_at | Spry4 | sprouty homolog 4 (Drosophila) | -1.4 | -3.0 | -2.1 |
| 1425769_x_at | Cklf | chemokine-like factor | -1.2 | -2.6 | -2.1 |
| 1436311_at | Gemin5 | gem (nuclear organelle) associated protein 5 | -2.3 | -4.9 | -2.1 |
| 1416059_at | Sec23b | SEC23B (S. cerevisiae) | -1.2 | -2.6 | -2.1 |
| 1437179_at | Rif1 | Rap1 interacting factor 1 homolog (yeast) | -3.9 | -8.3 | -2.1 |
| 1428869_at | Nolc1 | nucleolar and coiled-body phosphoprotein 1 | -2.1 | -4.4 | -2.1 |
| 1437398_a_at | Aldh9a1 | aldehyde dehydrogenase 9, subfamily A1 | -1.5 | -3.1 | -2.1 |
| 1425326_at | Acly | ATP citrate lyase | -3.4 | -7.2 | -2.1 |
| 1420441_at | Cenpc1 | centromere autoantigen C1 | -1.8 | -3.8 | -2.1 |
| 1450705_at | Rdbp | RD RNA-binding protein | -1.4 | -2.9 | -2.1 |
| 1421334_x_at | Mynn | myoneurin | -1.7 | -3.6 | -2.1 |
| 1426629_at | Dhx8 | DEAH (Asp-Glu-Ala-His) box polypeptide 8 | -1.4 | -3.0 | -2.1 |
| 1426789_s_at | Ssrp1 | structure specific recognition protein 1 | -1.1 | -2.2 | -2.1 |
| 1436883_at | Mbtps2 | membrane-bound transcription factor peptidase, site 2 | -1.2 | -2.6 | -2.1 |
| 1448315_a_at | Pycr2 | pyrroline-5-carboxylate reductase family, member 2 | -2.3 | -4.8 | -2.1 |
| 1433471_at | Tcf7 | transcription factor 7, T-cell specific | -1.7 | -3.6 | -2.1 |
| 1452712_at | Hnrpa3 | heterogeneous nuclear ribonucleoprotein A3 | -1.2 | -2.5 | -2.1 |
| 1429911_at | Mcph1 | microcephaly, primary autosomal recessive 1 | -3.1 | -6.6 | -2.1 |
| 1415867_at | Cct4 | chaperonin subunit 4 (delta) | -1.3 | -2.7 | -2.1 |
| 1417719_at | Sap30 | sin3 associated polypeptide | -1.6 | -3.4 | -2.1 |
| 1426798_a_at | Ppp1r15b | protein phosphatase 1, regulatory (inhibitor) subunit 15b | -2.1 | -4.4 | -2.1 |
| 1417299_at | Nek2 | NIMA (never in mitosis gene a)-related expressed kinase 2 | -2.6 | -5.4 | -2.1 |
| 1456674_at | Rad54b | RAD54 homolog B (S. cerevisiae) | -1.3 | -2.8 | -2.1 |
| 1418359_at | Wbscr27 | Williams Beuren syndrome chromosome region 27 (human) | -1.7 | -3.4 | -2.1 |
| 1425706_a_at | Ddb2 | damage specific DNA binding protein 2 | -1.5 | -3.2 | -2.1 |
| 1434972_x_at | Sfrs1 | splicing factor, arginine/serine-rich 1 (ASF/SF2) | -1.1 | -2.3 | -2.1 |
| 1416962_at | Rcc1 | regulator of chromosome condensation 1 | -2.1 | -4.4 | -2.1 |
| 1448834_at | Foxm1 | forkhead box M1 | -1.4 | -3.0 | -2.1 |
| 1416735_at | Asah1 | N-acylsphingosine amidohydrolase 1 | -1.3 | -2.8 | -2.1 |
| 1426751_s_at | Nup107 | nucleoporin 107 | -1.9 | -4.0 | -2.1 |
| 1433519_at | Nucks1 | nuclear casein kinase and cyclin-dependent kinase substrate 1 | -1.3 | -2.6 | -2.1 |
| 1429943_at | Ctbs | chitobiase, di-N-acetyl- | -1.4 | -2.9 | -2.1 |
| 1424205_at | Smarca5 | SWI/SNF related, matrix associated, actin dependent regulator of chromatin, subfamily a, member 5 | -1.4 | -2.8 | -2.1 |
| 1428061_at | Hat1 | histone aminotransferase 1 | -2.5 | -5.2 | -2.1 |
| 1423517_at | Cct6a | chaperonin subunit 6a (zeta) | -1.4 | -2.8 | -2.1 |
| 1417454_at | Cul4b | cullin 4B | -1.6 | -3.2 | -2.1 |
| 1425473_at | Crsp6 | cofactor required for Sp1 transcriptional activation, subunit 6 | -1.6 | -3.4 | -2.0 |
| 1418681_at | Glt28d1 | glycosyltransferase 28 domain containing 1 | -2.7 | -5.6 | -2.0 |
| 1427770_a_at | Slc2a3 | solute carrier family 2 (facilitated glucose transporter), member 3 | -1.4 | -2.8 | -2.0 |
| 1427047_at | Nup188 | nucleoporin 188 | -2.2 | -4.5 | -2.0 |
| 1421985_a_at | Eif4e2 | eukaryotic translation initiation factor 4E member 2 | -1.3 | -2.5 | -2.0 |
| 1453019_at | Nvl | nuclear VCP-like | -1.8 | -3.7 | -2.0 |
| 1439091_at | Fancd2 | Fanconi anemia, complementation group D2 | -3.0 | -6.0 | -2.0 |
| 1452207_at | Cited2 | Cbp/p300-interacting transactivator, with Glu/Asp-rich carboxy-terminal domain, 2 | -1.5 | -3.0 | -2.0 |
| 1416015_s_at | Abce1 | ATP-binding cassette, sub-family E (OABP), member 1 | -1.5 | -3.0 | -2.0 |
| 1423735_a_at | Wdr36 | WD repeat domain 36 | -1.8 | -3.6 | -2.0 |
| 1416073_a_at | Pcnt1 | pericentrin 1 | -2.4 | -4.8 | -2.0 |
| 1442135_at | Gm237 | Gene model 237, (NCBI) (Gm237), mRNA | -1.4 | -2.8 | -2.0 |
| 1449913_at | Zfp2 | zinc finger protein 2 | -1.3 | -2.5 | -2.0 |
| 1437942_x_at | Tube1 | epsilon-tubulin 1 | -1.1 | -2.3 | -2.0 |

**Subcluster B**

| **Probe sets** | Symbol | **Titel** | **fc d0**  **vs. d15** | **fc d0**  **vs. αMHC^+^** | **fc d15**  **vs. αMHC^+^** |
| --- | --- | --- | --- | --- | --- |
| 1438200_at | Sulf1 | sulfatase 1 | 3.1 | -14.6 | -44.6 |
| 1422587_at | Tmem45a | transmembrane protein 45a | 6.4 | -4.5 | -29.2 |
| 1417868_a_at | Ctsz | cathepsin Z | 2.4 | -11.4 | -27.4 |
| 1448326_a_at | Crabp1 | cellular retinoic acid binding protein I | 12.6 | -2.2 | -27.1 |
| 1448201_at | Sfrp2 | secreted frizzled-related sequence protein 2 | 11.1 | -2.4 | -26.7 |
| 1450699_at | Selenbp1 | selenium binding protein 1 | 6.9 | -3.7 | -25.8 |
| 1436319_at | Sulf1 | sulfatase 1 | 2.7 | -9.3 | -25.5 |
| 1416414_at | Emilin1 | elastin microfibril interfacer 1 | 11.1 | -2.2 | -24.2 |
| 1415871_at | Tgfbi | transforming growth factor, beta induced | 2.8 | -6.3 | -17.6 |
| 1417870_x_at | Ctsz | cathepsin Z | 2.5 | -6.4 | -15.8 |
| 1453304_s_at | Ly6e | lymphocyte antigen 6 complex, locus E | 2.3 | -6.8 | -15.5 |
| 1435477_s_at | Fcgr2b | Fc receptor, IgG, low affinity IIb | 5.4 | -2.4 | -12.7 |
| 1433977_at | Hs3st3b1 | heparan sulfate (glucosamine) 3-O-sulfotransferase 3B1 | 4.7 | -2.7 | -12.6 |
| 1417845_at | Cldn6 | claudin 6 | 3.5 | -3.5 | -12.3 |
| 1422824_s_at | Eps8 | epidermal growth factor receptor pathway substrate 8 | 2.0 | -5.8 | -11.6 |
| 1422823_at | Eps8 | epidermal growth factor receptor pathway substrate 8 | 2.2 | -5.1 | -11.5 |
| 1416978_at | Fcgrt | Fc receptor, IgG, alpha chain transporter | 3.9 | -2.9 | -11.4 |
| 1428579_at | Fmnl2 | formin-like 2 | 1.9 | -5.5 | -10.6 |
| 1417697_at | Soat1 | sterol O-acyltransferase 1 | 4.5 | -2.0 | -9.2 |
| 1434809_at | Arhgap28 | Rho GTPase activating protein 28 | 3.0 | -3.0 | -9.1 |
| 1454268_a_at | Cyba | cytochrome b-245, alpha polypeptide | 2.6 | -3.5 | -9.0 |
| 1420903_at | St6galnac3 | ST6 (alpha-N-acetyl-neuraminyl-2,3-beta-galactosyl-1,3)-N-acetylgalactosaminide alpha-2,6-sialyltransferase 3 | 1.9 | -4.1 | -7.5 |
| 1428306_at | Ddit4 | DNA-damage-inducible transcript 4 | 2.9 | -2.5 | -7.3 |
| 1418788_at | Tek | endothelial-specific receptor tyrosine kinase | 1.8 | -4.0 | -7.1 |
| 1455840_at | Rapgef5 | Rap guanine nucleotide exchange factor (GEF) 5 | 1.8 | -3.8 | -7.0 |
| 1429239_a_at | Stard4 | StAR-related lipid transfer (START) domain containing 4 | 2.4 | -2.8 | -6.7 |
| 1422438_at | Ephx1 | epoxide hydrolase 1, microsomal | 2.4 | -2.3 | -5.6 |
| 1449125_at | Tnfaip8l1 | tumor necrosis factor, alpha-induced protein 8-like 1 | 2.4 | -2.3 | -5.6 |
| 1423089_at | Tmod3 | tropomodulin 3 | 1.7 | -3.2 | -5.4 |
| 1429028_at | Dock11 | dedicator of cytokinesis 11 | 1.9 | -2.6 | -5.0 |
| 1424613_at | Gprc5b | G protein-coupled receptor, family C, group 5, member B | 1.6 | -2.9 | -4.7 |
| 1424265_at | Npl | N-acetylneuraminate pyruvate lyase | 1.7 | -2.8 | -4.6 |
| 1417388_at | Bex2 | brain expressed X-linked 2 | 1.7 | -2.5 | -4.2 |
| 1438214_at | Trps1 | trichorhinophalangeal syndrome I (human) | 1.6 | -2.7 | -4.2 |
| 1423924_s_at | Tspan14 | tetraspanin 14 | 1.6 | -2.6 | -4.2 |
| 1451346_at | Mtap | methylthioadenosine phosphorylase | 1.9 | -2.2 | -4.1 |
| 1422610_s_at | Igf2bp3 | insulin-like growth factor 2, binding protein 3 | 1.5 | -2.7 | -4.1 |
| 1418634_at | Notch1 | Notch gene homolog 1 (Drosophila) | 1.5 | -2.7 | -4.0 |
| 1416267_at | Scoc | short coiled-coil protein | 1.9 | -2.1 | -4.0 |
| 1423174_a_at | Pard6b | par-6 (partitioning defective 6) homolog beta (C. elegans) | 1.9 | -2.1 | -4.0 |
| 1428645_at | Gnai3 | guanine nucleotide binding protein, alpha inhibiting 3 | 1.8 | -2.2 | -3.9 |
| 1455137_at | Rapgef5 | Rap guanine nucleotide exchange factor (GEF) 5 | 1.6 | -2.5 | -3.9 |
| 1416206_at | Sipa1 | signal-induced proliferation associated gene 1 | 1.4 | -2.6 | -3.7 |
| 1424445_at | Tm4sf5 | transmembrane 4 superfamily member 5 | 1.7 | -2.2 | -3.7 |
| 1434010_at | Als2cr13 | amyotrophic lateral sclerosis 2 (juvenile) chromosome region, candidate 13 (human) | 1.8 | -2.1 | -3.7 |
| 1416511_a_at | Cdc42ep4 | CDC42 effector protein (Rho GTPase binding) 4 | 1.6 | -2.3 | -3.6 |
| 1426915_at | Dapk1 | death associated protein kinase 1 | 1.5 | -2.3 | -3.6 |
| 1435349_at | Nrp2 | neuropilin 2 | 1.5 | -2.3 | -3.5 |
| 1439427_at | Cldn9 | claudin 9 | 1.4 | -2.5 | -3.5 |
| 1435823_x_at | Egfl7 | EGF-like domain 7 | 1.6 | -2.1 | -3.3 |
| 1434777_at | Lmyc1 | lung carcinoma myc related oncogene 1 | 1.4 | -2.3 | -3.3 |
| 1434028_at | Arnt2 | aryl hydrocarbon receptor nuclear translocator 2 | 1.6 | -2.0 | -3.2 |
| 1439518_at | Mmrn2 | multimerin 2 | 1.5 | -2.2 | -3.2 |
| 1417365_a_at | Calm1 | calmodulin 1 | 1.5 | -2.1 | -3.2 |
| 1418294_at | Epb4.1l4b | erythrocyte protein band 4.1-like 4b | 1.5 | -2.1 | -3.2 |
| 1428896_at | Pdgfrl | platelet-derived growth factor receptor-like | 1.4 | -2.2 | -3.0 |
| 1455011_at | Stard4 | StAR-related lipid transfer (START) domain containing 4 | 1.4 | -2.2 | -3.0 |
| 1416675_s_at | Plcd1 | phospholipase C, delta 1 | 1.5 | -2.0 | -3.0 |
| 1417366_s_at | Calm1 | calmodulin 1 | 1.4 | -2.1 | -2.9 |
| 1418538_at | Kdelr3 | KDEL (Lys-Asp-Glu-Leu) endoplasmic reticulum protein retention receptor 3 | 1.3 | -2.1 | -2.9 |

**Subcluster C**

| **Probe sets** | Symbol | **Title** | **fc d0**  **vs. d15** | **fc d0**  **vs. αMHC^+^** | **fc d15**  **vs. αMHC^+^** |
| --- | --- | --- | --- | --- | --- |
| 1415856_at | Emb | embigin | 1.5 | -21.8 | -33.6 |
| 1459679_s_at | Myo1b | myosin IB | 1.1 | -16.4 | -17.4 |
| 1415857_at | Emb | embigin | 1.7 | -9.6 | -16.8 |
| 1423088_at | Tmod3 | tropomodulin 3 | 1.4 | -10.6 | -14.9 |
| 1418969_at | Skp2 | S-phase kinase-associated protein 2 (p45) | -1.1 | -16.0 | -14.0 |
| 1452514_a_at | Kit | kit oncogene | -1.1 | -11.9 | -10.6 |
| 1424128_x_at | Aurkb | aurora kinase B | -1.2 | -12.1 | -10.3 |
| 1449164_at | Cd68 | CD68 antigen | 1.1 | -8.8 | -10.1 |
| 1451246_s_at | Aurkb | aurora kinase B | -1.4 | -13.3 | -9.8 |
| 1448314_at | Cdc2a | cell division cycle 2 homolog A (S. pombe) | -1.3 | -12.8 | -9.7 |
| 1448647_at | Man2a1 | mannosidase 2, alpha 1 | 1.5 | -6.1 | -9.3 |
| 1417926_at | Luzp5 | leucine zipper protein 5 | -1.4 | -12.3 | -9.0 |
| 1428834_at | Dusp4 | dual specificity phosphatase 4 | 1.4 | -6.5 | -9.0 |
| 1460578_at | Fgd5 | FYVE, RhoGEF and PH domain containing 5 | 1.5 | -5.5 | -8.5 |
| 1455521_at | Klf12 | Kruppel-like factor 12 | 1.3 | -6.4 | -8.0 |
| 1429759_at | Rps6ka6 | ribosomal protein S6 kinase polypeptide 6 | 1.0 | -7.9 | -8.0 |
| 1437123_at | Mmrn2 | multimerin 2 | 1.6 | -5.0 | -7.9 |
| 1451740_at | Paip1 | polyadenylate binding protein-interacting protein 1 | -1.1 | -8.6 | -7.8 |
| 1434628_a_at | Rhpn2 | rhophilin, Rho GTPase binding protein 2 | -1.0 | -7.9 | -7.6 |
| 1448990_a_at | Myo1b | myosin IB | -1.1 | -8.3 | -7.4 |
| 1454659_at | Dctd | dCMP deaminase | -1.1 | -8.1 | -7.4 |
| 1415874_at | Spry1 | sprouty homolog 1 (Drosophila) | 1.6 | -4.7 | -7.4 |
| 1449065_at | Acot1 | acyl-CoA thioesterase 1 | 1.0 | -7.2 | -7.3 |
| 1448989_a_at | Myo1b | myosin IB | -1.1 | -7.3 | -6.9 |
| 1450687_at | Igf2bp3 | insulin-like growth factor 2, binding protein 3 | 1.3 | -5.2 | -6.8 |
| 1431873_a_at | Tube1 | epsilon-tubulin 1 | -1.2 | -8.2 | -6.6 |
| 1429234_s_at | 11Sep | septin 11 | 1.5 | -4.2 | -6.2 |
| 1427450_x_at | Myo1b | myosin IB | -1.0 | -6.2 | -6.1 |
| 1428853_at | Ptch1 | patched homolog 1 | -1.2 | -7.4 | -6.1 |
| 1449530_at | Trps1 | trichorhinophalangeal syndrome I (human) | 1.4 | -4.5 | -6.1 |
| 1416762_at | S100a10 | S100 calcium binding protein A10 (calpactin) | 1.2 | -5.2 | -6.0 |
| 1455695_at | St8sia1 | ST8 alpha-N-acetyl-neuraminide alpha-2,8-sialyltransferase 1 | -1.3 | -8.0 | -5.9 |
| 1433942_at | Myo6 | myosin VI | 1.5 | -3.8 | -5.8 |
| 1426817_at | Mki67 | antigen identified by monoclonal antibody Ki 67 | -1.0 | -6.0 | -5.8 |
| 1448441_at | Cks1b | CDC28 protein kinase 1b | -1.1 | -5.9 | -5.5 |
| 1431805_a_at | Rhpn2 | rhophilin, Rho GTPase binding protein 2 | -1.2 | -6.3 | -5.5 |
| 1420502_at | Sat1 | spermidine/spermine N1-acetyl transferase 1 | 1.3 | -4.1 | -5.4 |
| 1437208_at | 10Sep | septin 10 | 1.3 | -4.0 | -5.3 |
| 1419456_at | Dcxr | dicarbonyl L-xylulose reductase | 1.3 | -4.2 | -5.3 |
| 1434149_at | Tcf4 | transcription factor 4 | 1.5 | -3.5 | -5.1 |
| 1424495_a_at | Cklf | chemokine-like factor | -1.1 | -5.6 | -5.1 |
| 1426529_a_at | Tagln2 | transgelin 2 | 1.2 | -4.3 | -5.0 |
| 1424874_a_at | Ptbp1 | polypyrimidine tract binding protein 1 | -1.3 | -6.3 | -5.0 |
| 1450731_s_at | Tnfrsf21 | tumor necrosis factor receptor superfamily, member 21 | -1.2 | -5.9 | -5.0 |
| 1447788_s_at | Tspyl3 | TSPY-like 3 | -1.0 | -5.0 | -4.9 |
| 1450931_at | Dock9 | dedicator of cytokinesis 9 | 1.1 | -4.5 | -4.9 |
| 1428190_at | Slc25a1 | solute carrier family 25 (mitochondrial carrier, citrate transporter), member 1 | -1.3 | -6.4 | -4.9 |
| 1424713_at | Calml4 | calmodulin-like 4 | 1.3 | -3.8 | -4.8 |
| 1423161_s_at | Spred1 | sprouty protein with EVH-1 domain 1, related sequence | 1.1 | -4.3 | -4.8 |
| 1450932_s_at | Dock9 | dedicator of cytokinesis 9 | 1.1 | -4.4 | -4.8 |
| 1424292_at | Depdc1a | DEP domain containing 1a | 1.0 | -4.6 | -4.8 |
| 1450841_at | Itm1 | intergral membrane protein 1 | 1.2 | -3.9 | -4.7 |
| 1460173_at | Lasp1 | LIM and SH3 protein 1 | -1.3 | -5.9 | -4.6 |
| 1423319_at | Hhex | hematopoietically expressed homeobox | 1.2 | -3.7 | -4.4 |
| 1451428_x_at | Egfl7 | EGF-like domain 7 | 1.4 | -3.1 | -4.4 |
| 1420518_a_at | Igsf9 | immunoglobulin superfamily, member 9 | 1.4 | -3.1 | -4.4 |
| 1424895_at | Gpsm2 | G-protein signalling modulator 2 (AGS3-like, C. elegans) | 1.5 | -3.0 | -4.4 |
| 1459894_at | Iqgap2 | IQ motif containing GTPase activating protein 2 | -1.2 | -5.2 | -4.3 |
| 1426955_at | Col18a1 | procollagen, type XVIII, alpha 1 | -1.2 | -5.2 | -4.3 |
| 1423531_a_at | Hnrpa1 | heterogeneous nuclear ribonucleoprotein A1 | 1.2 | -3.6 | -4.3 |
| 1457424_at | Eya1 | eyes absent 1 homolog (Drosophila) | 1.1 | -3.7 | -4.2 |
| 1452857_at | MGI:2675296 | HCF-binding transcription factor Zhangfei | 1.3 | -3.2 | -4.2 |
| 1455834_x_at | Tacc3 | transforming, acidic coiled-coil containing protein 3 | 1.1 | -3.8 | -4.1 |
| 1455719_at | Tubb5 | tubulin, beta 5 | -1.1 | -4.6 | -4.1 |
| 1421963_a_at | Cdc25b | cell division cycle 25 homolog B (S. cerevisiae) | -1.2 | -4.7 | -4.1 |
| 1433490_s_at | Epb4.1l2 | erythrocyte protein band 4.1-like 2 | 1.2 | -3.4 | -4.1 |
| 1454625_at | Phf6 | PHD finger protein 6 | 1.3 | -3.0 | -4.0 |
| 1426903_at | Fndc3 | Fibronectin type III domain containing 3a, mRNA (cDNA clone IMAGE:5101040) | 1.4 | -2.8 | -4.0 |
| 1434406_at | Srgap2 | SLIT-ROBO Rho GTPase activating protein 2 | 1.1 | -3.5 | -4.0 |
| 1417125_at | Ahcy | S-adenosylhomocysteine hydrolase | -1.1 | -4.3 | -4.0 |
| 1435155_at | Cgn | cingulin | 1.1 | -3.6 | -3.9 |
| 1419123_a_at | Pdgfc | platelet-derived growth factor, C polypeptide | 1.2 | -3.2 | -3.8 |
| 1449351_s_at | Pdgfc | platelet-derived growth factor, C polypeptide | 1.2 | -3.2 | -3.8 |
| 1451427_a_at | Egfl7 | EGF-like domain 7 | 1.3 | -2.8 | -3.8 |
| 1429612_at | Eml4 | echinoderm microtubule associated protein like 4 | 1.2 | -3.1 | -3.8 |
| 1423683_at | Cdca4 | cell division cycle associated 4 | -1.1 | -4.1 | -3.7 |
| 1439847_s_at | Klf12 | Kruppel-like factor 12 | 1.2 | -3.1 | -3.7 |
| 1455819_at | Rod1 | ROD1 regulator of differentiation 1 (S. pombe) | 1.3 | -2.8 | -3.6 |
| 1454927_at | Zfp41 | zinc finger protein 41 | -1.0 | -3.6 | -3.5 |
| 1436071_at | Ankrd26 | ankyrin repeat domain 26 | -1.4 | -4.9 | -3.5 |
| 1419417_at | Vegfc | vascular endothelial growth factor C | 1.1 | -3.2 | -3.5 |
| 1451255_at | MGI:1927471 | liver-specific bHLH-Zip transcription factor | 1.4 | -2.5 | -3.5 |
| 1427229_at | Hmgcr | 3-hydroxy-3-methylglutaryl-Coenzyme A reductase | -1.1 | -3.8 | -3.5 |
| 1418260_at | Hunk | hormonally upregulated Neu-associated kinase | -1.0 | -3.6 | -3.5 |
| 1447112_s_at | Cryl1 | crystallin, lamda 1 | 1.2 | -2.9 | -3.4 |
| 1416795_at | Cryl1 | crystallin, lamda 1 | 1.1 | -3.1 | -3.4 |
| 1428136_at | Sfrp1 | secreted frizzled-related sequence protein 1 | 1.3 | -2.6 | -3.4 |
| 1460346_at | Arsa | arylsulfatase A | 1.0 | -3.3 | -3.4 |
| 1434224_at | Tbl2 | transducin (beta)-like 2 | 1.2 | -2.9 | -3.4 |
| 1428825_at | Nr6a1 | nuclear receptor subfamily 6, group A, member 1 | -1.0 | -3.4 | -3.4 |
| 1453159_at | Efhc1 | EF-hand domain (C-terminal) containing 1 | 1.0 | -3.3 | -3.3 |
| 1443924_at | Wnk3 | WNK lysine deficient protein kinase 3 | -1.1 | -3.6 | -3.3 |
| 1429233_at | 11Sep | septin 11 | 1.3 | -2.5 | -3.2 |
| 1423160_at | Spred1 | sprouty protein with EVH-1 domain 1, related sequence | 1.1 | -3.0 | -3.2 |
| 1420907_at | Cd2ap | CD2-associated protein | 1.3 | -2.6 | -3.2 |
| 1416886_at | MGI:1927354 | nuclear DNA binding protein | -1.1 | -3.5 | -3.2 |
| 1415878_at | Rrm1 | ribonucleotide reductase M1 | 1.0 | -3.2 | -3.2 |
| 1436425_at | Ankrd38 | ankyrin repeat domain 38 | 1.3 | -2.4 | -3.2 |
| 1438666_at | Ldlrad3 | low density lipoprotein receptor class A domain containing 3 | 1.0 | -3.1 | -3.2 |
| 1438559_x_at | Slc44a2 | solute carrier family 44, member 2 | 1.3 | -2.4 | -3.2 |
| 1448401_at | Smarcd2 | SWI/SNF related, matrix associated, actin dependent regulator of chromatin, subfamily d, member 2 | -1.1 | -3.6 | -3.2 |
| 1437627_at | Rkhd1 | ring finger (C3HC4 type) and KH domain containing 1 | -1.2 | -3.7 | -3.1 |
| 1436890_at | Uap1l1 | UDP-N-acteylglucosamine pyrophosphorylase 1-like 1 | -1.1 | -3.4 | -3.1 |
| 1423818_a_at | Arl6ip1 | ADP-ribosylation factor-like 6 interacting protein 1 | -1.1 | -3.3 | -3.0 |
| 1452648_at | Tbrg1 | transforming growth factor beta regulated gene 1 | 1.3 | -2.3 | -3.0 |
| 1437643_at | Cenpj | centromere protein J | -1.2 | -3.5 | -3.0 |
| 1423322_at | Lin7c | lin 7 homolog c (C. elegans) | -1.1 | -3.2 | -3.0 |
| 1417741_at | Pygl | liver glycogen phosphorylase | 1.1 | -2.6 | -2.9 |
| 1424041_s_at | C1s | complement component 1, s subcomponent | -1.0 | -3.0 | -2.9 |
| 1421934_at | Cbx5 | chromobox homolog 5 (Drosophila HP1a) | 1.1 | -2.6 | -2.9 |
| 1420478_at | Nap1l1 | nucleosome assembly protein 1-like 1 | 1.2 | -2.4 | -2.9 |
| 1434027_at | Dscr1l2 | Down syndrome critical region gene 1-like 2 | 1.3 | -2.3 | -2.8 |
| 1433946_at | Zik1 | zinc finger protein interacting with K protein 1 | -1.0 | -2.9 | -2.8 |
| 1434559_at | Stx3 | syntaxin 3 | -1.1 | -3.2 | -2.8 |
| 1419749_at | Dnmt2 | DNA methyltransferase 2 | 1.0 | -2.7 | -2.8 |
| 1448791_at | Snx5 | sorting nexin 5 | -1.1 | -3.0 | -2.8 |
| 1430388_a_at | Sulf2 | sulfatase 2 | 1.1 | -2.5 | -2.7 |
| 1451254_at | Ikbkap | inhibitor of kappa light polypeptide enhancer in B-cells, kinase complex-associated protein | 1.0 | -2.6 | -2.7 |
| 1458218_s_at | Pde7a | phosphodiesterase 7A | 1.1 | -2.6 | -2.7 |
| 1455049_at | Igsf3 | immunoglobulin superfamily, member 3 | 1.2 | -2.4 | -2.7 |
| 1435093_at | Zfyve20 | zinc finger, FYVE domain containing 20 | 1.1 | -2.4 | -2.7 |
| 1448432_at | Plcd1 | phospholipase C, delta 1 | 1.2 | -2.3 | -2.7 |
| 1419645_at | Cstf2 | cleavage stimulation factor, 3' pre-RNA subunit 2 | 1.1 | -2.4 | -2.7 |
| 1455048_at | Igsf3 | immunoglobulin superfamily, member 3 | 1.2 | -2.2 | -2.7 |
| 1433835_at | Ppp3cb | protein phosphatase 3, catalytic subunit, beta isoform | 1.1 | -2.5 | -2.7 |
| 1449146_at | Notch4 | Notch gene homolog 4 (Drosophila) | 1.0 | -2.6 | -2.7 |
| 1416925_at | Kpnb1 | karyopherin (importin) beta 1 | -1.0 | -2.7 | -2.7 |
| 1440739_at | Vegfc | vascular endothelial growth factor C | 1.0 | -2.6 | -2.7 |
| 1429776_a_at | Dnajb6 | DnaJ (Hsp40) homolog, subfamily B, member 6 | -1.2 | -3.2 | -2.6 |
| 1423241_a_at | Tfdp1 | transcription factor Dp 1 | 1.1 | -2.3 | -2.6 |
| 1452036_a_at | Tmpo | thymopoietin | 1.0 | -2.5 | -2.6 |
| 1428492_at | Glipr2 | GLI pathogenesis-related 2 | -1.1 | -2.9 | -2.6 |
| 1437972_s_at | Sf3b5 | splicing factor 3b, subunit 5 | 1.1 | -2.4 | -2.6 |
| 1452190_at | Prcp | prolylcarboxypeptidase (angiotensinase C) | 1.3 | -2.0 | -2.6 |
| 1428546_at | Syncrip | synaptotagmin binding, cytoplasmic RNA interacting protein | 1.0 | -2.6 | -2.6 |
| 1423325_at | Pnn | pinin | -1.1 | -2.7 | -2.6 |
| 1452490_a_at | Ap2a2 | adaptor protein complex AP-2, alpha 2 subunit | 1.1 | -2.4 | -2.6 |
| 1427567_a_at | Tpm3 | tropomyosin 3, gamma | -1.1 | -2.7 | -2.6 |
| 1455727_at | U2af1-rs2 | U2 small nuclear ribonucleoprotein auxiliary factor (U2AF) 1, related sequence 2 | 1.1 | -2.2 | -2.6 |
| 1418844_at | Dibd1 | disrupted in bipolar disorder 1 homolog (human) | 1.2 | -2.1 | -2.6 |
| 1460633_at | Prp19 | PRP19/PSO4 homolog (S. cerevisiae) | -1.0 | -2.6 | -2.6 |
| 1451185_at | Sf3b5 | splicing factor 3b, subunit 5 | 1.1 | -2.4 | -2.6 |
| 1449093_at | Ctf1 | cardiotrophin 1 | 1.2 | -2.1 | -2.6 |
| 1452220_at | Dock1 | dedicator of cyto-kinesis 1 | 1.2 | -2.1 | -2.5 |
| 1433491_at | Epb4.1l2 | erythrocyte protein band 4.1-like 2 | 1.0 | -2.4 | -2.5 |
| 1418545_at | Wasf1 | WASP family 1 | -1.2 | -3.0 | -2.5 |
| 1427764_a_at | Tcfe2a | transcription factor E2a | -1.1 | -2.7 | -2.5 |
| 1428911_at | Ttll4 | tubulin tyrosine ligase-like family, member 4 | -1.1 | -2.7 | -2.5 |
| 1428157_at | Gng2 | guanine nucleotide binding protein (G protein), gamma 2 subunit | -1.0 | -2.6 | -2.5 |
| 1429227_x_at | Nap1l1 | nucleosome assembly protein 1-like 1 | 1.0 | -2.4 | -2.5 |
| 1437921_x_at | Zfp516 | zinc finger protein 516 | -1.1 | -2.8 | -2.5 |
| 1428446_at | MGI:1913996 | dynein 2 light intermediate chain | -1.1 | -2.6 | -2.5 |
| 1424210_at | Spfh1 | SPFH domain family, member 1 | 1.1 | -2.3 | -2.5 |
| 1433444_at | Hmgcs1 | 3-hydroxy-3-methylglutaryl-Coenzyme A synthase 1 | -1.0 | -2.5 | -2.4 |
| 1424110_a_at | Nme1 | expressed in non-metastatic cells 1, protein | 1.2 | -2.0 | -2.4 |
| 1450150_a_at | Rpl13 | ribosomal protein L13 | -1.2 | -2.7 | -2.4 |
| 1434530_at | Odz4 | odd Oz/ten-m homolog 4 (Drosophila) | 1.1 | -2.2 | -2.3 |
| 1426907_s_at | Dhx57 | DEAH (Asp-Glu-Ala-Asp/His) box polypeptide 57 | -1.2 | -2.7 | -2.3 |
| 1437471_at | Lrrc45 | leucine rich repeat containing 45 | 1.0 | -2.2 | -2.3 |
| 1433826_at | Tspyl3 | TSPY-like 3 | -1.1 | -2.4 | -2.3 |
| 1435174_at | Rsbn1 | rosbin, round spermatid basic protein 1 | 1.0 | -2.2 | -2.3 |
| 1420829_a_at | Ywhaq | tyrosine 3-monooxygenase/tryptophan 5-monooxygenase activation protein, theta polypeptide | -1.0 | -2.4 | -2.3 |
| 1418377_a_at | MGI:1353606 | Cd27 binding protein (Hindu God of destruction) | -1.1 | -2.5 | -2.3 |
| 1435897_at | Rpl32 | ribosomal protein L32 | -1.2 | -2.7 | -2.3 |
| 1434737_at | Obfc1 | oligonucleotide/oligosaccharide-binding fold containing 1 | -1.0 | -2.3 | -2.3 |
| 1434724_at | Usp31 | ubiquitin specific peptidase 31 | 1.0 | -2.2 | -2.2 |
| 1450011_at | Hsd17b12 | hydroxysteroid (17-beta) dehydrogenase 12 | -1.0 | -2.3 | -2.2 |
| 1433844_a_at | Dusp9 | dual specificity phosphatase 9 | 1.0 | -2.2 | -2.2 |
| 1427120_at | Zfp26 | zinc finger protein 26 | 1.1 | -2.0 | -2.2 |
| 1420479_a_at | Nap1l1 | nucleosome assembly protein 1-like 1 | -1.0 | -2.3 | -2.2 |
| 1426380_at | Eif4b | eukaryotic translation initiation factor 4B | -1.2 | -2.7 | -2.2 |
| 1449202_at | Sema4g | sema domain, immunoglobulin domain (Ig), transmembrane domain (TM) and short cytoplasmic domain, (semaphorin) 4G | 1.1 | -2.0 | -2.2 |
| 1459981_s_at | Rsbn1 | rosbin, round spermatid basic protein 1 | 1.1 | -2.0 | -2.2 |
| 1452350_at | Brd8 | bromodomain containing 8 | -1.0 | -2.2 | -2.2 |
| 1449176_a_at | Dck | deoxycytidine kinase | 1.1 | -2.0 | -2.2 |
| 1452778_x_at | Nap1l1 | nucleosome assembly protein 1-like 1 | 1.0 | -2.1 | -2.2 |
| 1416742_at | Cfdp1 | craniofacial development protein 1 | -1.1 | -2.3 | -2.1 |
| 1452254_at | Mtmr9 | myotubularin related protein 9 | -1.0 | -2.2 | -2.1 |
| 1418442_at | Xpo1 | exportin 1, CRM1 homolog (yeast) | -1.0 | -2.2 | -2.1 |
| 1460238_at | Msln | mesothelin | 1.0 | -2.1 | -2.1 |
| 1454666_at | Klf3 | Kruppel-like factor 3 (basic) | 1.0 | -2.0 | -2.1 |
| 1417364_at | Eef1g | eukaryotic translation elongation factor 1 gamma | -1.1 | -2.3 | -2.1 |
| 1417340_at | Txnl2 | thioredoxin-like 2 | -1.1 | -2.3 | -2.1 |
| 1420865_at | Zfp161 | zinc finger protein 161 | -1.0 | -2.1 | -2.0 |
| 1417126_a_at | Rpl22l1 | ribosomal protein L22 like 1 | -1.0 | -2.1 | -2.0 |
| 1425255_s_at | Hnrpll | heterogeneous nuclear ribonucleoprotein L-like | -1.0 | -2.0 | -2.0 |

Lists of probe sets for the subclusters A, B and C as identified in the hierarchical clustering of probe sets downregulated in α-MHC^+^ cells (Fig. 6). Probe sets are listed with the corresponding gene symbol and gene title. Fold changes (fc) are given for pairwise comparisons between undifferentiated α-MHC ES cells (d0) and day 15 control EBs (d15), between undifferentiated α-MHC ES cells (d0) and 15 day old α-MHC^+^ cardiomyocytes (α-MHC^+^) as well as between day 15 control EBs (d15) and 15 day old α-MHC^+^ cardiomyocytes (α-MHC^+^).
