# Supplementary material for: Global transcriptome analysis of murine embryonic stem cell-derived cardiomyocytes
Source: Genome Biol. 2007 Apr 11;8(4):R56. doi: 10.1186/gb-2007-8-4-r56 (PMC1896009; doi:10.1186/gb-2007-8-4-r56)
Supplement: Additional data file 9 — Part a provides genes belonging to the KEGG pathway 'cell cycle' as well as to the GO BP terms 'M-phase', 'mitotic cell cycle', and 'regulation of cell cycle' that are downregulated in α-MHC+ cardiomyocytes (intersection of downregulation in α-MHC+ cardiomyocytes [twofold, t-test P value < 0.01] compared with control cells in the 15-day-old control EBs and compared with undifferentiated α-MHC ES cells). It also provides a schematic of the KEGG cell cycle pathway, indicating the downregulated genes. Part b provides genes belonging to the BIOCARTA pathways 'G1/S checkpoint' and 'G2/M checkpoint' that are downregulated in α-MHC+ cardiomyocytes (intersection of downregulation in α-MHC+ cardiomyocytes [twofold, t-test P value < 0.01) compared with control cells in the 15-day-old control EBs and compared with undifferentiated α-MHC ES cells). Part c provides genes belonging to the GOTERM_BP_5 categories 'positive regulation of programmed cell death' that are downregulated in α-MHC+ cardiomyocytes (intersection of downregulation in α-MHC+ cardiomyocytes [twofold, t-test P value < 0.01] compared with control cells in the 15-day-old control EBs and compared with undifferentiated α-MHC ES cells). [file gb-2007-8-4-r56-S9.doc]

**Additional Data file 9a**

| **Probe Set** | **Symbol** | **Title** | **fc d0 vs.**  **d15** | **fc d0 vs.**  **αMHC^+^** | **fc d15 vs.**  **αMHC^+^** |
| --- | --- | --- | --- | --- | --- |
| 1424046_at | Bub1 | budding uninhibited by benzimidazoles 1 homolog (S. cerevisiae) | -2.6 | -51.0 | -19.7 |
| 1418969_at | Skp2 | S-phase kinase-associated protein 2 (p45) | -1.1 | -16.0 | -14.0 |
| 1417019_a_at | Cdc6 | cell division cycle 6 homolog (S. cerevisiae) | -2.6 | -35.8 | -13.9 |
| 1424629_at | Brca1 | breast cancer 1 | -3.7 | -50.0 | -13.4 |
| 1435306_a_at | Kif11 | kinesin family member 11 | -2.7 | -34.2 | -12.9 |
| 1416309_at | Nusap1 | nucleolar and spindle associated protein 1 | -1.7 | -20.7 | -12.1 |
| 1448314_at | Cdc2a | cell division cycle 2 homolog A (S. pombe) | -1.3 | -12.8 | -9.7 |
| 1426652_at | Mcm3 | minichromosome maintenance deficient 3 (S. cerevisiae) | -3.0 | -27.0 | -9.2 |
| 1426002_a_at | Cdc7 | cell division cycle 7 (S. cerevisiae) | -1.2 | -10.4 | -8.8 |
| 1417450_a_at | Tacc3 | transforming, acidic coiled-coil containing protein 3 | -1.7 | -13.7 | -8.3 |
| 1422814_at | Aspm | asp (abnormal spindle)-like, microcephaly associated (Drosophila) | -2.3 | -17.5 | -7.7 |
| 1416251_at | Mcm6 | minichromosome maintenance deficient 6 (MIS5 homolog, S. pombe) | -2.4 | -18.1 | -7.4 |
| 1429658_a_at | Smc2l1 | SMC2 structural maintenance of chromosomes 2-like 1 (yeast) | -2.2 | -16.0 | -7.4 |
| 1439208_at | Chek1 | checkpoint kinase 1 homolog (S. pombe) | -1.9 | -13.8 | -7.2 |
| 1452954_at | Ube2c | ubiquitin-conjugating enzyme E2C | -1.8 | -12.6 | -7.1 |
| 1456280_at | Clspn | claspin homolog (Xenopus laevis) | -1.5 | -10.4 | -7.0 |
| 1422513_at | Ccnf | cyclin F | -2.7 | -18.6 | -7.0 |
| 1448191_at | Plk1 | polo-like kinase 1 (Drosophila) | -1.9 | -13.4 | -7.0 |
| 1422016_a_at | Cenph | centromere autoantigen H | -2.9 | -19.4 | -6.7 |
| 1437580_s_at | Nek2 | NIMA (never in mitosis gene a)-related expressed kinase 2 | -2.6 | -17.3 | -6.6 |
| 1431873_a_at | Tube1 | epsilon-tubulin 1 | -1.2 | -8.2 | -6.6 |
| 1416961_at | Bub1b | budding uninhibited by benzimidazoles 1 homolog, beta (S. cerevisiae) | -2.8 | -18.1 | -6.4 |
| 1423920_at | Brrn1 | barren homolog (Drosophila) | -1.8 | -11.4 | -6.3 |
| 1448414_at | Rad1 | RAD1 homolog (S. pombe) | -3.0 | -18.2 | -6.0 |
| 1433862_at | Espl1 | extra spindle poles-like 1 (S. cerevisiae) | -3.0 | -18.1 | -6.0 |
| 1435005_at | Cenpe | centromere protein E | -1.8 | -10.4 | -5.9 |
| 1426817_at | Mki67 | antigen identified by monoclonal antibody Ki 67 | -1.0 | -6.0 | -5.8 |
| 1424105_a_at | Pttg1 | pituitary tumor-transforming 1 | -1.4 | -8.0 | -5.6 |
| 1448205_at | Ccnb1-rs1; Ccnb1 | cyclin B1, related sequence 1; cyclin B1 | -2.3 | -12.9 | -5.5 |
| 1450920_at | Ccnb2 | cyclin B2 | -2.5 | -13.8 | -5.5 |
| 1422460_at | Mad2l1 | MAD2 (mitotic arrest deficient, homolog)-like 1 (yeast) | -1.7 | -9.0 | -5.3 |
| 1416664_at | Cdc20 | cell division cycle 20 homolog (S. cerevisiae) | -2.0 | -10.6 | -5.3 |
| 1424144_at | Ris2 | retroviral integration site 2 | -2.9 | -15.1 | -5.2 |
| 1418334_at | AA545217 | expressed sequence AA545217 | -3.3 | -16.3 | -4.9 |
| 1416214_at | Mcm4 | minichromosome maintenance deficient 4 homolog (S. cerevisiae) | -1.7 | -8.1 | -4.6 |
| 1418380_at | Terf1 | telomeric repeat binding factor 1 | -3.0 | -13.3 | -4.4 |
| 1417910_at | Ccna2 | cyclin A2 | -1.7 | -7.5 | -4.3 |
| 1417445_at | Kntc2 | kinetochore associated 2 | -2.7 | -11.6 | -4.3 |
| 1450886_at | Gsg2 | germ cell-specific gene 2 | -3.6 | -15.2 | -4.3 |
| 1421963_a_at | Cdc25b | cell division cycle 25 homolog B (S. cerevisiae) | -1.2 | -4.7 | -4.1 |
| 1436808_x_at | Mcm5 | minichromosome maintenance deficient 5, cell division cycle 46 | -2.1 | -8.5 | -4.0 |
| 1430811_a_at | Cdca1 | cell division cycle associated 1 | -1.5 | -5.7 | -3.9 |
| 1419123_a_at | Pdgfc | platelet-derived growth factor, C polypeptide | 1.2 | -3.2 | -3.8 |
| 1416031_s_at | Mcm7 | minichromosome maintenance deficient 7 (S. cerevisiae) | -2.7 | -10.1 | -3.7 |
| 1416206_at | Sipa1 | signal-induced proliferation associated gene 1 | 1.4 | -2.6 | -3.7 |
| 1416988_at | Msh2 | mutS homolog 2 (E. coli) | -3.5 | -13.0 | -3.7 |
| 1416746_at | H2afx | H2A histone family, member X | -1.9 | -7.0 | -3.6 |
| 1460229_at | Stag3 | stromal antigen 3 | -6.7 | -23.6 | -3.5 |
| 1419417_at | Vegfc | vascular endothelial growth factor C | 1.1 | -3.2 | -3.5 |
| 1424156_at | Rbl1 | retinoblastoma-like 1 (p107) | -1.4 | -5.0 | -3.5 |
| 1417506_at | Gmnn | geminin | -2.2 | -7.5 | -3.4 |
| 1434777_at | Lmyc1 | lung carcinoma myc related oncogene 1 | 1.4 | -2.3 | -3.3 |
| 1452241_at | Topbp1 | topoisomerase (DNA) II beta binding protein | -1.9 | -6.2 | -3.3 |
| 1433892_at | Spag5 | sperm associated antigen 5 | -2.6 | -8.1 | -3.2 |
| 1430574_at | Cdkn3 | cyclin-dependent kinase inhibitor 3 | -1.4 | -4.2 | -3.1 |
| 1423848_at | Mphosph6 | M phase phosphoprotein 6 (Mphosph6), mRNA | -1.3 | -3.9 | -2.9 |
| 1422252_a_at | Cdc25c | cell division cycle 25 homolog C (S. cerevisiae) | -1.5 | -4.3 | -2.8 |
| 1416575_at | Cdc45l | cell division cycle 45 homolog (S. cerevisiae)-like | -2.6 | -7.4 | -2.8 |
| 1416748_a_at | Mre11a | meiotic recombination 11 homolog A (S. cerevisiae) | -1.7 | -4.6 | -2.8 |
| 1422663_at | Orc1l | origin recognition complex, subunit 1-like (S.cereviaiae) | -4.5 | -11.9 | -2.6 |
| 1423241_a_at | Tfdp1 | transcription factor Dp 1 | 1.1 | -2.3 | -2.6 |
| 1426538_a_at | Trp53 | transformation related protein 53 | -2.8 | -7.0 | -2.5 |
| 1448638_at | Mtbp | Mdm2, transformed 3T3 cell double minute p53 binding protein | -2.7 | -6.6 | -2.5 |
| 1415849_s_at | Stmn1 | stathmin 1 | -1.5 | -3.7 | -2.5 |
| 1417132_at | Cdc25a | cell division cycle 25 homolog A (S. cerevisiae) | -1.4 | -3.6 | -2.5 |
| 1452197_at | Smc4l1 | SMC4 structural maintenance of chromosomes 4-like 1 (yeast) | -1.5 | -3.8 | -2.5 |
| 1417832_at | Smc1l1 | SMC (structural maintenance of chromosomes 1)-like 1 (S. cerevisiae) | -1.6 | -3.9 | -2.5 |
| 1422547_at | Ranbp1 | RAN binding protein 1 | -1.6 | -3.9 | -2.4 |
| 1441910_x_at | Ccne1 | cyclin E1 | -3.4 | -7.7 | -2.3 |
| 1434079_s_at | Mcm2 | minichromosome maintenance deficient 2 mitotin (S. cerevisiae) | -2.3 | -5.2 | -2.2 |
| 1416962_at | Rcc1 | regulator of chromosome condensation 1 | -2.1 | -4.4 | -2.1 |

Genes belonging to the KEGG pathway “Cell Cycle” as well as to the GO terms BP, “M-phase”, “Mitotic Cell Cycle”, and “Regulation of Cell Cycle” that are downregulated in α-MHC^+^ cardiomyocytes (intersection of downregulation in α-MHC^+^ cardiomyocytes (2-fold, t-test p-value < 0.01) compared to control cells in the 15 day control EBs (d15) and to undifferentiated α-MHC ES cells (d0)).

Fold changes (fc) are given for pairwise comparisons between undifferentiated α-MHC ES cells (d0) and day 15 control EBs (d15), between undifferentiated α-MHC ES cells (d0) and 15 day old α-MHC^+^ cardiomyocytes (α-MHC^+^) as well as between day 15 control EBs (d15) and 15 day old α-MHC^+^ cardiomyocytes (α-MHC^+^).

Schematic of the KEGG Cell Cycle pathway indicating the downregulated genes (labelled with red background and white letters).

**Additional Data file 9b**

| Probe Set | **Symbol** | **Title** | **fc d0 *vs.***  **d15** | **fc d0 *vs.* αMHC^+^** | **fc d15 *vs.* αMHC^+^** |
| --- | --- | --- | --- | --- | --- |
| 1418969_at | Skp2 | S-phase kinase-associated protein 2 (p45) | -1.1 | -16.0 | -14.0 |
| 1424629_at | Brca1 | breast cancer 1 | -3.7 | -50.0 | -13.4 |
| 1448314_at | Cdc2a | cell division cycle 2 homolog A (S. pombe) | -1.3 | -12.8 | -9.7 |
| 1439208_at | Chek1 | checkpoint kinase 1 homolog (S. pombe) | -1.9 | -13.8 | -7.2 |
| 1448191_at | Plk1 | polo-like kinase 1 (Drosophila) | -1.9 | -13.4 | -7.0 |
| 1417910_at | Ccna2 | cyclin A2 | -1.7 | -7.5 | -4.3 |
| 1422252_a_at | Cdc25c | cell division cycle 25 homolog C (S. cerevisiae) | -1.5 | -4.3 | -2.8 |
| 1423241_a_at | Tfdp1 | transcription factor Dp 1 | 1.1 | -2.3 | -2.6 |
| 1426538_a_at | Trp53 | transformation related protein 53 | -2.8 | -7.0 | -2.5 |
| 1417132_at | Cdc25a | cell division cycle 25 homolog A (S. cerevisiae) | -1.4 | -3.6 | -2.5 |
| 1420829_a_at | Ywhaq | tyrosine 3-monooxygenase/tryptophan 5-monooxygenase act. protein, theta polypept. | -1.0 | -2.4 | -2.3 |
| 1441910_x_at | Ccne1 | cyclin E1 | -3.4 | -7.7 | -2.3 |

Genes belonging to the Biocarta Pathways “G1/S Checkpoint” and “G2/M Checkpoint” that are downregulated in α-MHC^+^ cardiomyocytes (intersection of downregulation in α-MHC^+^ cardiomyocytes (2-fold, t-test p-value < 0.01) compared to control cells in the 15 day control EBs (d15) and to undifferentiated α-MHC ES cells (d0)).

Fold changes (fc) are given for pairwise comparisons between undifferentiated α-MHC ES cells (d0) and day 15 control EBs (d15), between undifferentiated α-MHC ES cells (d0) and 15 day old α-MHC^+^ cardiomyocytes (α-MHC^+^) as well as between day 15 control EBs (d15) and 15 day old α-MHC^+^ cardiomyocytes (α-MHC^+^).


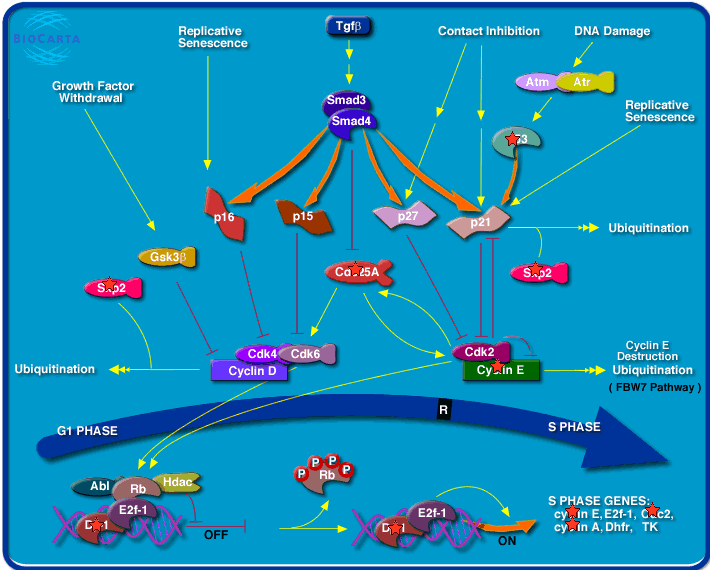

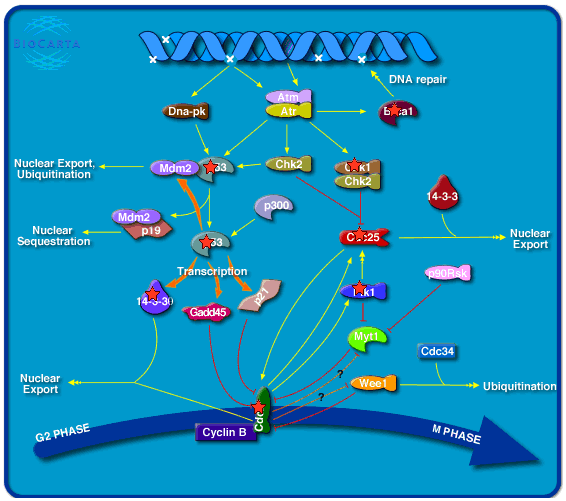


Schematic of the Biocarta pathways “G1/S Checkpoint” and “G2/M Checkpoint” indicating the downregulated genes (labelled with red stars).

**Additional Data file 9**

| **Probe Set** | **Symbol** | **Title** | **fc d0**  ***vs.* d15** | **fc d0 *vs.***  **αMHC^+^** | **fc d15 *vs.***  **αMHC^+^** |
| --- | --- | --- | --- | --- | --- |
| 1424629_at | Brca1 | breast cancer 1 | -3.7 | -50.0 | -13.4 |
| 1426955_at | Col18a1 | procollagen, type XVIII, alpha 1 | -1.2 | -5.2 | -4.3 |
| 1455719_at | Tubb5 | tubulin, beta 5 | -1.1 | -4.6 | -4.1 |
| 1418634_at | Notch1 | Notch gene homolog 1 (Drosophila) | 1.5 | -2.7 | -4.0 |
| 1416206_at | Sipa1 | signal-induced proliferation associated gene 1 | 1.4 | -2.6 | -3.7 |
| 1426915_at | Dapk1 | death associated protein kinase 1 | 1.5 | -2.3 | -3.6 |
| 1416915_at | Msh6 | mutS homolog 6 (E. coli) | -4.6 | -15.7 | -3.4 |
| 1449490_at | Mbd4 | methyl-CpG binding domain protein 4 | -1.7 | -4.6 | -2.7 |
| 1426538_a_at | Trp53 | transformation related protein 53 | -2.8 | -7.0 | -2.5 |
| 1418377_a_at | Siva | Cd27 binding protein (Hindu God of destruction) | -1.1 | -2.5 | -2.3 |

Genes belonging to the GOTERM_BP_5, “POSITIVE REGULATION OF PROGRAMMED CELL DEATH” that are downregulated in α-MHC^+^ cardiomyocytes (intersection of downregulation in α-MHC^+^ cardiomyocytes (2-fold, t-test p-value < 0.01) compared to control cells in the 15 day control EBs (d15) and to undifferentiated α-MHC ES cells (d0)).

Fold changes (fc) are given for pairwise comparisons between undifferentiated α-MHC ES cells (d0) and day 15 control EBs (d15), between undifferentiated α-MHC ES cells (d0) and 15 day old α-MHC^+^ cardiomyocytes (α-MHC^+^) as well as between day 15 control EBs (d15) and 15 day old α-MHC^+^ cardiomyocytes (α-MHC^+^).
