# Supplementary material for: Global transcriptome analysis of murine embryonic stem cell-derived cardiomyocytes
Source: Genome Biol. 2007 Apr 11;8(4):R56. doi: 10.1186/gb-2007-8-4-r56 (PMC1896009; doi:10.1186/gb-2007-8-4-r56)
Supplement: Additional data file 10 — Provided are probe sets both differentially regulated by puromycin treatment in 15-day-old β-actin EBs (twofold, Student's t-test P value < 0.01) and transcripts upregulated in α-MHC+ cells (intersection of upregulation in the α-MHC+ cardiomyocytes [twofold, Student's t-test P value < 0.01] compared with the control cells in the 15-day-old EBs and in the undifferentiated α-MHC ES cells). [file gb-2007-8-4-r56-S10.doc]

## Additional data file 10

| Probe sets | **Symbol** | **fc β-Actin Puro 15d vs. contr. 15d** | **fc control d15 vs. αMHC^+^** |
| --- | --- | --- | --- |
| 1424367_a_at | Homer2 | -2.16 | 8.50 |
| 1457671_at | 9330120H11Rik | -2.23 | 8.21 |
| 1449501_a_at | Gzmm | -2.05 | 5.74 |
| 1434960_at | Taf9l | -3.65 | 5.35 |
| 1455091_at | Msl2 | -3.71 | 4.99 |
| 1428547_at | Nt5e | -5.89 | 4.56 |
| 1455901_at | Chpt1 | -3.84 | 4.52 |
| 1460344_at | 2310033F14Rik | -2.28 | 4.04 |
| 1437724_x_at | Pitpnm1 | -2.09 | 3.59 |
| 1453710_at | C030022K24Rik | -2.23 | 3.44 |
| 1454646_at | Tcp11l2 | -3.05 | 3.33 |
| 1437869_at | 3222402P14Rik | -3.2 | 3.29 |
| 1420833_at | Vamp2 | -2.32 | 2.63 |
| 1452213_at | Tex2 | -2.16 | 2.41 |
| 1436510_a_at | Lrrfip2 | -2.09 | 2.26 |
| 1452357_at | Gp1bb; Sept5 | -5.61 | 2.25 |
| 1450791_at | Nppb | -2.96 | 2.18 |
| 1456482_at | Pik3r3 | -2.5 | 2.14 |
| 1423632_at | Gpr146 | -2.43 | 2.13 |
| 1434683_at | Cutl1 | -2.51 | 2.08 |
| 1452207_at | Cited2 | -2.07 | -2.02 |
| 1427630_x_at | Ceacam1 | -3.09 | -2.20 |
| 1415978_at | Tubb3 | -2.54 | -2.27 |
| 1425538_x_at | Ceacam1 | -4.51 | -2.42 |
| 1416897_at | Parp9 | -2.41 | -2.82 |
| 1424713_at | Calml4 | -2.32 | -4.82 |
| 1430700_a_at | Pla2g7 | -2.82 | -6.07 |
| 1450995_at | Folr1 | -2.85 | -6.34 |
| 1457465_at | D430043L16Rik | -3.13 | -6.99 |
| 1433977_at | Hs3st3b1 | -2.04 | -12.60 |
| 1453304_s_at | Ly6e | -2.58 | -15.50 |

List of probe sets both differentially regulated by puromycin treatment in 15-day old β-actin EBs (2-fold, Student’s t-test p-value < 0.01) and transcripts upregulated in α-MHC^+^ cells (intersection of up-regulation in the α-MHC^+^ cardiomyocytes (2-fold, Student’s t-test p-value < 0.01) compared to the control cells in the 15-day old EBs and in the undifferentiated α-MHC ES cells).

Probe sets are listed with the corresponding gene symbol. Fold changes (fc) are given for the pairwise comparison between control EBs and puromycin-treated EBs at day 15 in the β-actin cell line (third column) and for the pairwise comparison between day 15 control EBs (d15) and 15 day old α-MHC^+^ cardiomyocytes (α-MHC^+^) in the α-MHC^+^ cell line (fourth column). Probe sets are ordered according to the fold change in column 4.
